# Supplementary material for: Modeling the health and economic implications of adopting a 1-dose 9-valent human papillomavirus vaccination program in adolescents in low/middle-income countries: An analysis of Indonesia
Source: PLoS One. 2024 Nov 21;19(11):e0310591. doi: 10.1371/journal.pone.0310591 (PMC11581242; doi:10.1371/journal.pone.0310591)
Supplement: S1 Appendix — (PDF) [file pone.0310591.s001.pdf]

## Contents


Appendix A: Summary of recent health and economic outcomes models in LMIC ..... 2
Appendix B: Estimating 1-dose vaccine model parameters from clinical trial data ..... 5
B.1. Data ..... 5
B.2. Clinical Trial Cohort Model ..... 6
B.2.1. Waning Model Characteristics ..... 7
B.3. Bayesian generation of model parameter value distributions ..... 9
B.3.1. Estimating Force of Infection ..... 10
B.3.2. Generating Vaccine Model Parameter Distributions using only KEN-SHE 18- and 36-month results 10
B.3.3. Modifying the likelihood function to include India IARC efficacy data. ..... 11
B.3.4. Choosing n ..... 11
B.4. Results ..... 12
B.4.1. Base-case distribution ..... 12
B.4.2. Alternative distribution ..... 14
Appendix C: Model parameters ..... 16
C.1. Natural History Parameters ..... 16
C.2. Treatment Patterns ..... 22
C.3. Cancer fatality rate ..... 24
C.4. Vaccine Model Parameters ..... 24
Appendix D: Demographic Parameters ..... 26
D.1. All-Cause Mortality ..... 26
D.2. Fertility ..... 27
D.3. Sexual Activity Parameters ..... 27
D.3.1. Estimating Number of New Sexual Partners per Year ..... 27
Appendix E: Calibration Inputs and Results ..... 33
E.1. Type Attribution ..... 33
E.2. Cervical Pre-cancer Incidence ..... 33
E.3. Cancer Incidence and Deaths ..... 34
E.4. Cancer fatality rate ..... 36
E.5. Genital Warts Incidence and Prevalence ..... 36
E.6. Calibration Methods and Results ..... 37
E.6.1. Cervical Model Calibration ..... 37

E.6.2. Anal Model Calibration ..... 42
E.6.3. Genital Warts Calibration ..... 46
E.6.4. Oropharyngeal Model Calibration ..... 47
E.6.5. Penile Model Calibration ..... 50
E.6.6. Vaginal Model Calibration ..... 52
E.6.7. Vulvar Model Calibration ..... 55
Appendix F: Cost and Health Utility Inputs ..... 57
F.1. Treatment Costs ..... 57
F.2. Vaccine Price ..... 58
F.3. Health State Utilities ..... 58
F.3.1. Baseline utilities ..... 58
F.3.2. Cervical disease ..... 59
F.3.3. Vaginal, Vulvar, Penile ..... 59
F.3.4. Anal ..... 60
F.3.5. Oropharyngeal ..... 60
F.3.6. Genital Warts ..... 60
Appendix G: Additional Results ..... 61
G.1. Cases Avoided ..... 61
G.2. Cost Effectiveness Sensitivity ..... 61
G.2.1. Base Case Distribution ..... 61
G.2.2. Alternative Distribution ..... 72
G.3. Cervical cancer elimination ..... 81
G.3.1. Base Case Distribution ..... 81
G.3.2. Alternative Distribution ..... 85
References 90

# Appendix A: Summary of recent health and economic outcomes models in LMIC

The current goals of WHO's Cervical Cancer Elimination Initiative were informed by selected models.[1] The models identified were HPV-ADVISE, Harvard-HPV, and Policy1-Cervix. Of these three, the first two have been adapted to assess the cost-effectiveness of a single dose in LMICs. For example, versions of the agent-based Harvard-HPV transmission and static

individual-based Harvard cervical carcinogenesis (Harvard-CC) models that reflected sexual mixing patterns in the U.S population were linked together by Burger et al. (2018)[2] to assess the benefits of HPV vaccination in Uganda. They found that while 1-dose vaccination had high up-front costs, the prevention of cervical cancer cases led to long-term cost-effectiveness when compared with no vaccination. They also found that, unless 1-dose vaccination was able to achieve higher coverage and lifelong protection, the 2-dose vaccination schedule is cost-effective.

In a recent study using both HPV-ADVISE and Harvard-HPV, Burger et al. (2022)[3] quantified the health impacts of immediately introducing a single-dose HPV vaccination program in a high-burden setting (e.g., Uganda), compared with delaying HPV vaccine implementation until results from single-dose RCTs (e.g., ESCUDDO) become available (2025-26). Depending on the model used, and the assumptions made for single-dose vaccine efficacy, duration and coverage, the authors found that implementing a single-dose program would avert up to 9.6% additional cases over 2021-2120. The study supports the conclusion that an immediate 1-dose program is better than no program at all for the next 5 years; however, it did not compare the health and economic impacts of a 1-dose program with the immediate introduction of a 2-dose program.

Drolet et al. (2021)[4] conducted a modeling analysis to determine the most efficient and cost-effective HPV vaccination strategies in LMICs (e.g., India, Vietnam, Uganda, and Nigeria). They accounted for sex workers' larger role in HPV transmission in LMICs by adapting HPV-ADVISE to include female sex workers and their clients in the model. Among the 27 strategies explored, which varied the age of routine vaccination, number of age cohorts vaccinated, population targeted (girls versus girls and boys), and interval between doses, they found that the most efficient and cost-effective strategies involved vaccinating girls aged 9-14 with two doses. To optimize the number of doses required in the short-term to prevent cervical cancer with limited resources, the most effective strategies reported were (1) a 2-dose vaccine schedule for girls aged 13 or 14 years, with or without a later switch to routine vaccination at an earlier age, and (2) routine vaccination at age 9 or 10 years with 3-5 years between doses and catch up for girls aged 14 years.

A more recent study published by Man et al. (2022)[5] supported a single-dose schedule in India. The study projected the effect of different doses of the HPV vaccine at a subnational level by state. Overall, the study found that a single dose of the HPV vaccine may be a cost-effective alternative to a 2-dose schedule in low- and middle-income countries like India, conditioned on the single dose having high protection and on being able to achieve high vaccination coverage, which may not be realistic or feasible in some settings. The study projected that both 1-dose and 2-dose schedules in girls aged 10 years with 90% coverage could reduce the lifetime risk of cervical cancer by up to 79%. Additionally, single-dose HPV vaccination was found to be 21-100% more efficient in terms of cases prevented per dose under several scenarios considered for single-dose protection.

Finally, by synthesizing three independent HPV transmission models, including the linked Harvard model and HPV-ADVISE model, Prem et al. (2021)[6] compared the long-term health benefits and cost-effectiveness of no vaccination, 1-dose HPV vaccination, and 2-dose HPV vaccination in 192 countries. They found that with 80% coverage of 10-year-old girls, a single

HPV vaccine dose could avert 64-68 million HPV cases worldwide, and that fewer girls needed to be vaccinated with a 1-dose schedule to prevent one case in low-income countries than in middle and high-income countries. They also reported that a 2-dose schedule would avert an additional 2.1-8.7 million cases.

# Appendix B: Estimating 1-dose vaccine model parameters from clinical trial data

## B.1. Data

The joint distribution for the reduction factor of the degree and the duration of protection from a single dose regimen of HPV vaccine was estimated based on a Bayesian inference approach applied to data from the KEN SHE (ClinicalTrials.gov ID NCT03675256) study including 18-month data from Barnabas et al.[7] and 36-month data presented at IPVC 2023. The estimation analysis used the 18- and 36-month efficacy data from the vaccine and control arm incidence of persistent HPV 16/18/31/33/45/52/58 pooled populations and the HPV 16/18 pooled populations in the 9vHPV vaccine arm of the trial. Table 1 and Table 2 summarize the trial data outcomes for the HPV 16/18/31/33/45/52/58 pooled population and HPV 16/18 pooled populations at the two trial time points reported. These data will be used to infer the joint probability distributions for our base case.

*Table 1: Summary of relevant data from the KEN SHE trial 18- and 36-month results for the HPV 16/18 naive population (mITT)*

| | HPV 16/18 naïve (mITT) (n) | | Incident persistent cervical HPV 16/18 (n) | | Vaccine Efficacy (%) | | 95% CI | |
|---|---|---|---|---|---|---|---|---|
| Time (months) | 18 | 36 | 18 | 36 | 18 | 36 | 18 | 36 |
| Nonavalent HPV Vaccine | 496 | 496 | 1 | 1 | 97.49 | 98.8 | (81.66, 99.66) | (91.3, 99.8) |
| Meningoccoccal Vaccine | 473 | 473 | 36 | 72 | - | | - | - |

*Table 2: Summary of relevant data from the KEN SHE trial 18- and 36-month results for the HPV 16/18/31/33/45/52/58 naive population (mITT)*

| | HPV 16/18/31/33/45 /52/58 naïve (mITT) (n) | | Incident persistent cervical HPV 16/18/31/33/45 /52/58 (n) | | Vaccine Efficacy (%) | | 95% CI | |
|---|---|---|---|---|---|---|---|---|
| Time (months) | 18 | 36 | 18 | 36 | 18 | 36 | 18 | 36 |
| Nonavalent HPV Vaccine | 325 | 325 | 4 | 5 | 88.91 | 95.5 | (68.45, 96.10) | (89.0, 98.2) |
| Meningoccoccal Vaccine | 290 | 290 | 29 | 84 | - | | - | - |

Given the cumulative case counts from the trial populations, we applied a Bayesian analysis to estimate the distribution of model parameters fitting the trial data.  The vaccine models will make a few simplifying assumptions that are reasonable in the trial context.  First, we will assume that the prevailing force of infection during the trial is constant – this is reasonable since the general population is orders of magnitude larger than the trial population, the general population is unvaccinated, and the trial population is (assumed to be) mixing randomly with the general population; therefore, the trial population has negligible impact on the force of infection.  Second, we will model the entire trial population as a single age group without deaths.

In addition, we will also consider an alternative estimate of the distribution parameters where we include the above KEN SHE data results as well as the results from India IARC long-term follow up study.[8] Table 2 shows the efficacy results for HPV16/18 persistent infection that will be used in developing the alternative distribution of vaccine parameters.

*Table 3:  India IARC efficacy of one dose against persistent cervical infection after median follow-up time of nine years.*

| Vaccine | Adjusted vaccine efficacy against HPV 16/18 persistent infection | 95% CI |
|---|---|---|
| Gardasil | 95.4% | (85.0, 99.9) |

## B.2. Clinical Trial Cohort Model

To estimate the force of infection, λ, we will use the placebo group and make the further assumption that vaccine and placebo arms experience the same force of infection.  Figure 1 illustrates the control or placebo arm of the study.  SC(t) represents the control arm population with SC(0)=S0 (study control arm size), and IC(t) is the size of the persistently infected control population, with IC(0)=0.  This diagram represents the following system of differential equations:

$$SC'(t) = -\lambda\, SC(t) \quad SC(0) = S0$$
$$IC'(t) = \lambda\, SC(t) \quad IC(0) = 0$$

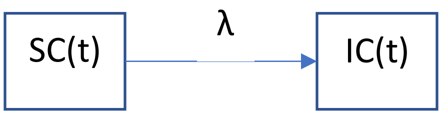


*Figure 1: Study unvaccinated (control) arm model*

For the vaccine arm of the study the model is illustrated in Figure 2, with $V_i(t)$ being the vaccinated population in the $i^{th}$ vaccine compartment (we are modeling waning with Gamma profile with a series of *n* successive compartments), IV(t) the persistently infected vaccinated population, and SV(t) the

population for which vaccine protection has waned (unprotected). In the model, ψ is the "degree of protection", ω is the waning rate, and λ is the force of infection.

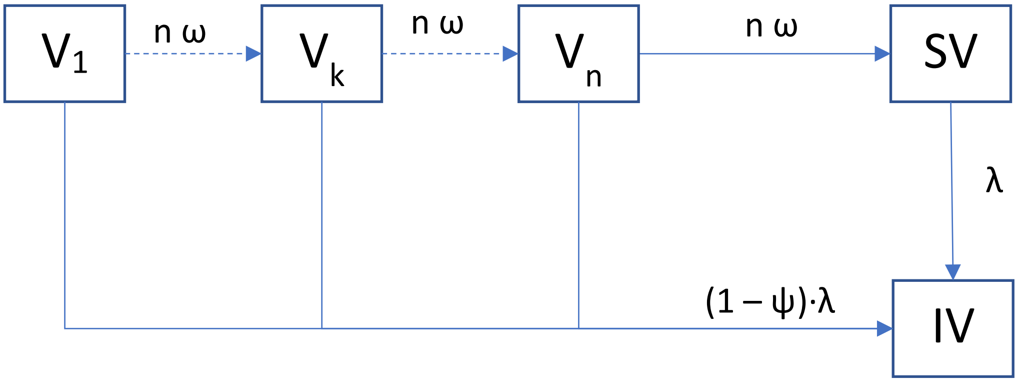


*Figure 2: Leaky multi-vaccinated/protected compartment with waning (Gamma waning profile)*

The differential equations for this system are:

$$\begin{aligned} V_1'(t) &= -(n\,\omega + (1-\psi)\,\lambda)\,V_1(t) \\ V_k'(t) &= n\,\omega\,V_{k-1}(t) - (n\,\omega + (1-\psi)\,\lambda)\,V_k(t) \quad ; \ 2 \le k \le n \\ SV'(t) &= n\,\omega\,V_n(t) - \lambda\,SV(t) \\ IV'(t) &= (1-\psi)\,\lambda \sum_{k=1}^{n} V_k(t) + \lambda\,SV(t) \\ V_1(0) &= V0 \\ V_k(0) &= 0 \quad ; \quad k > 1 \\ SV(0) &= 0 \\ IV(0) &= 0 \end{aligned} \tag{1}$$

The attack rate efficacy is given by:

$$Eff(t) = 1 - \frac{IV(t)}{IC(t)} \tag{2}$$

These equations can be solved explicitly; however, the resulting expressions are cumbersome, and, for large enough *n*, numerical evaluation is simpler, faster, and more stable.

### B.2.1. Waning Model Characteristics

Here we analyze the characteristics of the vaccine protection waning model. Consider only the vaccine compartments in the above model in the absence of exposure (λ=0). We can then solve this for the $V_k(t)$

$$V_k(t) = \frac{e^{-n\,\omega\,t} t^{k-1} (n\,\omega)^{k-1}}{(k-1)!}$$

where we have assumed V1(0)=1. The rate of leaving all vaccinated compartments due to waning alone is given by the rate of exit from the last vaccine compartment (k=n)

$$f(t) = n\,\omega\,V_n(t) = n\,\omega \frac{e^{-n\,\omega\,t} t^{n-1} (n\,\omega)^{n-1}}{(n-1)!}.$$

This corresponds to a probability density function of a random variable, T, of waiting time that is gamma distributed with shape parameter n and scale/rate parameter nω. The mean waiting time in the vaccinated compartments is then,

$$\mu_\tau = \int_0^\infty t\,f(t)dt = \frac{1}{\omega}$$

We associate this quantity $\mu_\tau$ with the "duration of protection" of the vaccinated population. The variance of this mean is

$$\sigma_\tau^2 = \int_0^\infty (\mu_\tau - t)^2\,f(t)dt = \frac{1}{n\,\omega^2}$$

So that the standard deviation of the mean duration of protection is

$$\sigma_\tau = \frac{1}{\sqrt{n}\,\omega} = \frac{\mu_\tau}{\sqrt{n}}$$

The standard deviation is proportional to the mean duration and inversely proportional to the square root of n. Figure 3 shows the total protected population (those in any one of the vaccinated compartments) as a function of time for three different values of n and a mean duration of protection of 20 years, as an example. When n=1 we have the constant waning rate resulting in exponential loss of protection. As n approaches infinity the loss of protection approaches a step function.

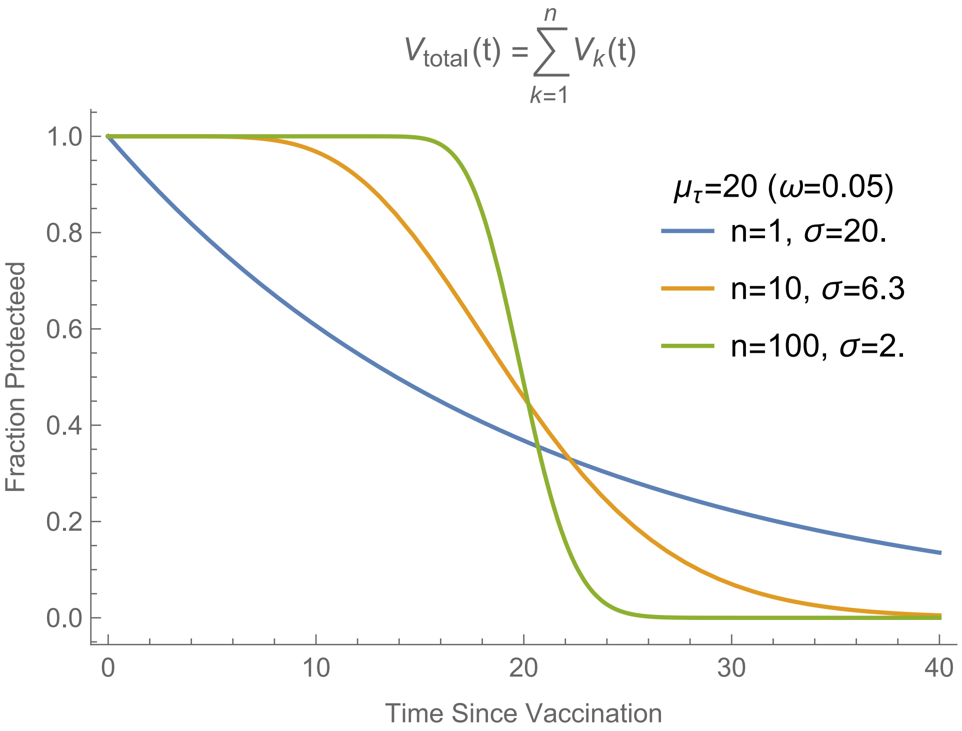


*Figure 3: Example showing the vaccine waning given a duration of protection of 20 years and showing three different values of n.*

## B.3. Bayesian generation of model parameter value distributions

To fit the model to the data and estimate the parameter uncertainty distribution we begin with Bayes theorem:

$$\pi(x|D) \propto p(x)\, L(D|x) \qquad (3$$

where $\pi(x|D)$ is the posterior distribution of the values of x (model parameter vector), given that we have observed D, data value vector. p(x) is the prior distribution and reflects our previous knowledge of possible values of x. L(D|x) is the likelihood that we would observe D given a particular value of x. The values of x will be plugged into our deterministic model (defined above) where the model produces a set of outcomes, $\{M_i(x)\}$, that are used in a statistical model of the observation process (clinical trials, in this case). Given vaccine properties, x, the vaccine model gives the probability of infection over time. The clinical trial data is then assumed to be realizations of Poisson random variables with parameters

given by the number of participants, $N_i$, in the arms of the clinical trial, and probabilities from the vaccine model (see for example[9]). Thus,

$$L(D|x) = \prod_{i=1}^{|D|} PoissonPDF(d_i; N_i, M_i(x))$$

## B.3.1. Estimating Force of Infection

We first estimate the average force of infection in the general population by constructing the likelihood function for the placebo arm of the two study populations (HPV 16/18, and HPV 16/18/31/33/45/52/58). This model has one parameter, λ, so that

$$M_i(x) = M_i(\lambda) = \frac{IC(t_i; \lambda)}{S0}$$

We then simply maximize L(D|λ) with respect to λ to get the estimated average forces of infection shown in Table 54:

*Table 4: Estimated prevailing force of infection for the two different populations:*

| Study Sub-population | Most likely λ |
|---|---|
| HPV 16/18 | 0.054221 |
| HPV 16/18/31/33/45/52/58 | 0.096724 |

## B.3.2. Generating Vaccine Model Parameter Distributions using only KEN-SHE 18- and 36-month results

Here the vaccine arm data outcome is the prevalence of infection in the vaccine arm at the observed time point, so that

$$M_i(x) = M_i(\psi, \omega, n) = \frac{IV(t_i; \psi, \omega, n)}{V0}$$

where $t_i$ is the time at which data value (prevalence of persistent infection in vaccinated population) $d_i$ is observed. We then generate $\pi(x|D)$ using the above definition of L(D|x) and the 18- and 36-month data from KEN-SHE (two time points) in an implementation of the Metropolis-Hastings Markov Chain Monte Carlo (MCMC) algorithm[10]. $N_i$ (size of trial arm population) and $d_i$ (number of positives) are taken from Table 1 (for HPV 6/11/16/18 distribution) and Table 2 (for HPV 31/33/45/52/58 distribution). For these data we have two time points, therefore |D|=2.

Finally, we define the priors p(x) to complete the algorithm. We still assume a "flat" prior over a specified range of values for the degree of protection and the duration of protection. We construct the prior for waning rate (inversely proportional to duration of protection) by assuming the duration is at least as long as the study duration, 3 years, then $p(\omega) = \theta(\frac{1}{3} - \omega)$, where $\omega \geq 0$ and θ(.) is the unit step

function and is 0 when the argument is negative and 1 otherwise. For the degree of protection, we base the prior on the expectation that the degree of protection due to one-dose cannot be greater than the degree of protection from full-series. For persistent cervical infection this is 0.988[a], so $p(\psi) = \theta(0.988 - \psi)$ and $0 \leq \psi \leq 1$. Finally, we assume a flat prior for n (the number of vaccine compartments) with $\{n \in \mathbb{Z} | 1 \leq n \leq 20\}$. This results in the prior

$$p(\omega, \psi, n) = \theta\left(\frac{1}{3} - \omega\right) \theta(0.988 - \psi)\, \theta(20 - n)$$

### B.3.3. Modifying the likelihood function to include India IARC efficacy data.

The India IARC 10-year follow-up trial data provides efficacy estimates at a median follow-up time of about 9 years for the single-dose population.[8] This was a 2-valent HPV (HPV 16/18) vaccine study and reported a pooled HPV 16/18 single-dose efficacy against persistent cervical HPV infection of 95.4% (95% CI 85.0 to 99.9). Since the study population, location, and time period of this study is completely different and the duration is substantially longer than KEN SHE, we cannot assume the same prevailing force of infection for all time; thus, the assumption of an average prevailing force of infection is not reasonable. However, if we assume that the vaccine efficacy is approximately independent of the force of infection, we can use efficacy instead of case-counts by making some assumptions about the distribution of the efficacy result and modifying the likelihood function.

First we assume that the vaccine efficacy at 9 years is distributed normally with a mean of μ=0.954 (efficacy at 9 years) and a standard deviation, $\delta = \frac{\mu - q_{0.025}}{1.95996} = \frac{0.954 - .850}{1.95996} = 0.05306$, which will be assumed to be the same for all model-generated values of efficacy. We now partition the data set D into the union of two sets, $D_{Norm}$, consisting of data values following the normal distribution, and $D_{Poisson}$, consisting of data values following the Poisson distribution.

$$L(D_{Norm} \cup D_{Poisson} | x) = \prod_{i=1}^{|D_{Norm}|} NormalPDF\left(d^{Norm}{}_i; \Sigma_i, M^{Norm}{}_i(x)\right) \prod_{i=1}^{|D_{Poisson}|} PoissonPDF\left(d_i; N_i, M_i(x)\right)$$

Here we have one data point that we're treating as a normally distributed random variable so $|D_{Norm}| = 1$, $d^{Norm}{}_1 = 0.954$ (the efficacy from India IARC at year $t_1 = 9$), $\Sigma_1 \equiv \sigma = 0.0531$, and, from Equation (2,

$$M^{Norm}{}_1(x) = M^{Norm}{}_1(\psi, \omega, n) = 1 - \frac{IV(t_1; \psi, \omega, n)}{IC(t_1)}$$

The remaining terms are as before, with $|D_{Poisson}| = 2$, representing the KEN SHE data. Finally, we will assume that the India IARC data can be applied to all sites and HPV types.

### B.3.4. Choosing n

To keep the size of the model from becoming too large and simplifying the expression of the parameter sets, we prefer to have a fixed value for n. To estimate a reasonable value for n based on the data we

---

[a] From analysis of Merck clinical trial data on file

generate the joint probability distribution for fixed n values ranging from 1 to 20. We then calculate the BIC, and mean and standard deviation of $(\psi, \omega)$ as a function of n. We observed that after n reaches values between 6 and 10 (depending on data set being fit), the BIC, mean, and standard deviations change very little. That is, the data does not inform us about the size of n after about n=6 to 10. Thus, we choose n=10 as large enough to represent the data and small enough that the computational cost is reasonable. We did this exercise for the base distribution (KEN-SHE data only) and the alternative distribution (KEN-SHE data plus India IARC data)

The posterior is the joint probability distribution with fixed n is , $\pi(\psi, \omega|D)$, is generated via Markov chain Monet Carlo (MCMC) method implemented in Mathematica©[11] using 5000 burn-in iterations and 50,000 sampling iterations. The resulting joint posterior distribution is used to draw pairs of vaccine parameters for the PSA.

## B.4. Results

The fractional reduction in degree of protection, $f_{\psi}$, is given by the distribution degree of protection, ψ, divided by 0.988 (the full-series degree of protection for persistent cervical infection). The "duration of protection" is defined as $t_{\mu}$ =1/ω, the mean time for protection to wane.

### B.4.1. Base-case distribution

Figure 4 shows the density plots of the vaccine model parameters resulting from fitting to the KEN SHE pooled HPV 16/18 (left panel) and the HPV 16/18/31/33/45/52/58 (right panel) population data, representing the base-case parameter distribution for HPV 6/11/16/18 and HPV 31/33/45/52/58. Table 5 shows several statistics from the two distributions including the median value of $f_{\psi}$, $t_{\mu}$, and the time it takes for the model efficacy to drop below 90%. The percentiles are estimated for each parameter from the joint distribution separately. The corresponding plots of efficacy as a function of time are shown in *Figure 4*.

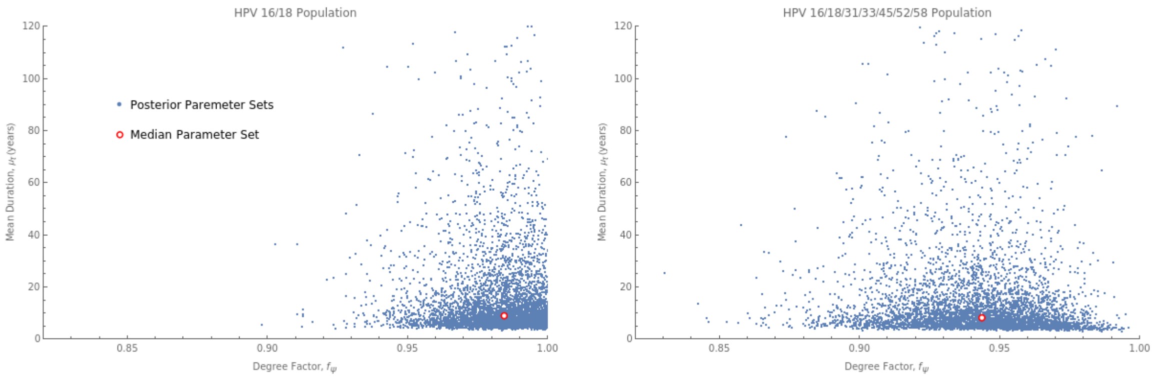

*Figure 4: Density plot of 5,000 pairs of 1-dose vaccine properties representing the base-case posterior distribution resulting from fitting HPV 16/18 and HPV 16/18/31/33/45/52/56 populations of KEN SHE 18 and 36-month data*

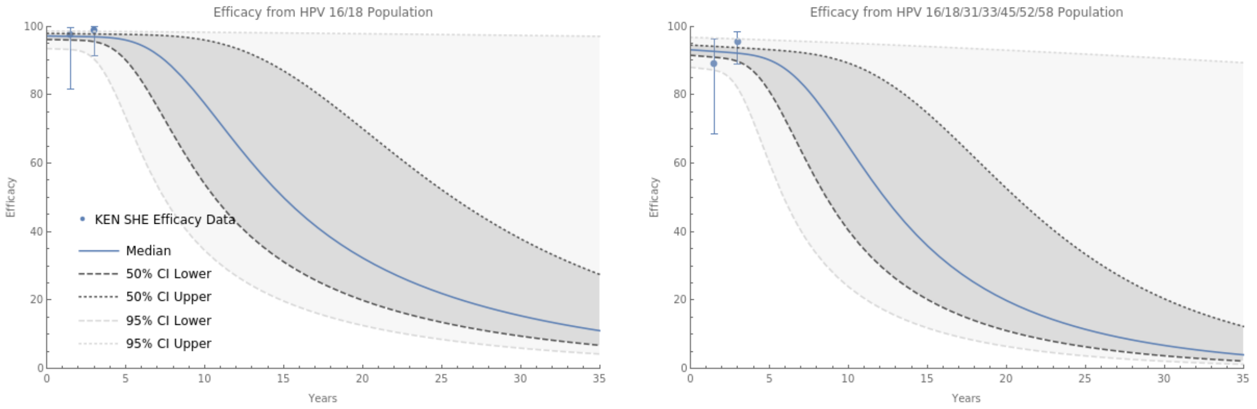


*Figure 5: Efficacy vs time plots resulting from each of the base-case posterior distributions. The blue line is the efficacy assuming the median parameter values (from Table 55). The gray regions represent the uncertainty in the efficacy estimate over time.*

*Table 5: Statistics from the KEN SHE 36 month data only (base-case) joint distribution resulting from fitting HPV 16/18 KEN SHE data (distribution used to represent effectiveness of HPV 6, 11, 16, or 18) and HPV 16/18/31/33/45/52/58 KEN SHE data (distribution representing HPV 31, 33, 45, 52, or 58)*

| | **Median of Degree Factor, $f_\psi$ [95% CI] (%)** | **Median of Duration of Protection, $t_\mu$ [95% CI] (years)** | **Median of Time until efficacy < 90% [95% CI] (years)** |
|---|---|---|---|
| HPV 6, 11, 16, or 18 | 98.44 [94.7, 99.92] | 9.1y [4.3, 121.9] | 7.5 [3.1, 78.4] |
| HPV 31, 33, 45, 52, or 58 | 94.36 [89.17, 98.03] | 8.3y [3.8, 98.7] | 5.1 [0.0, 32.9] |

From the numerical distributions presented (size 5,000) we draw 500 random sets of points for our PSA.

This fitting process results in one-dose vaccine model properties for cervical persistent infection only. In our model, we parameterize the vaccine for each different HPV type, site, and sex. To use the above results, we assume that the fractional reduction in degree of protection for any type, site, and sex combination is the same as the fractional degree of protection for cervical persistent infection. We then multiply this factor by the full-series degree of protection for each site, type, and sex combination. This ensures that the degree of protection provided by one dose is never greater than the degree of protection provided by the full series.

## B.4.2. Alternative distribution

*Figure 6* shows the density plots of the vaccine model parameters resulting from fitting to the KEN SHE pooled HPV 16/18 and the HPV 16/18/31/33/45/52/58 population data, as well as the India IARC 9-year follow-up efficacy data, representing the alternative-case parameter distribution for HPV 6/11/16/18 and HPV 31/33/45/52/58. Table 6 shows several statistics from the two distributions including the median value of $f_\psi$, $\mathbf{t}_\mu$, and the time it takes for the efficacy to drop below 90%. The corresponding plots of efficacy as a function of time are shown in Figure 7.

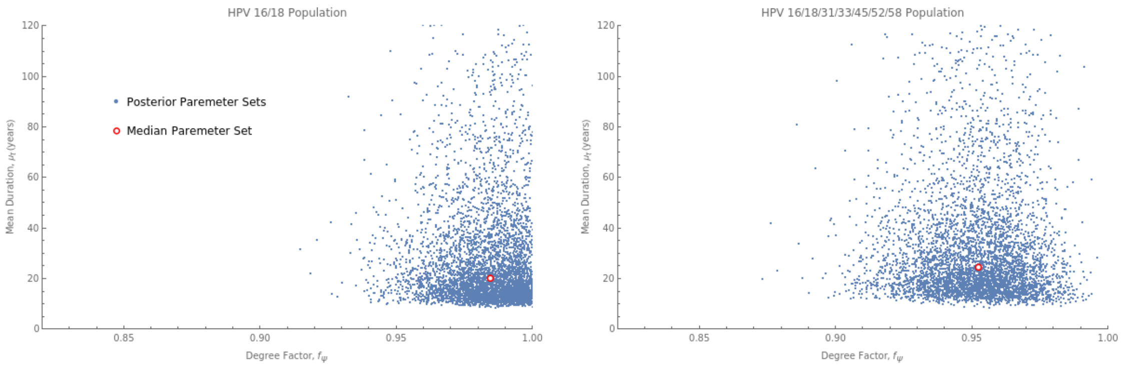


*Figure 6: Density plot of 5,000 pairs of 1-dose vaccine properties representing the alternative-case posterior distribution resulting from fitting HPV 16/18 and HPV 16/18/31/33/45/52/56 populations of KEN SHE 18 and 36-month data and the India-IARC 10-year follow-up efficacy data*

*Table 6: Statistics from the KEN SHE 36-month data plus India IARC (alternative-case) joint distribution resulting from fitting HPV 16/18 KEN SHE data (distribution used to represent effectiveness of HPV 6, 11, 16, or 18) and HPV 16/18/31/33/45/52/58 KEN SHE data (distribution used to represent effectiveness of HPV 31, 33, 45, 52, or 58)*

| | **Median of Degree Factor, $f_\psi$ [95% CI] (%)** | **Median of Duration of Protection, $t_\mu$ [95% CI] (years)** | **Median of Time until efficacy < 90% [95% CI] (years)** |
|---|---|---|---|
| HPV 6, 11, 16, or 18 | 98.44 [95.11, 99.90] | 20.3y [10.3, 301.5] | 15.8 [7.1, 137.8] |
| HPV 31, 33, 45, 52, or 58 | 95.23 [91.43, 98.12] | 24.6y [11.6, 333.3] | 11.7 [0.8, 34.3] |

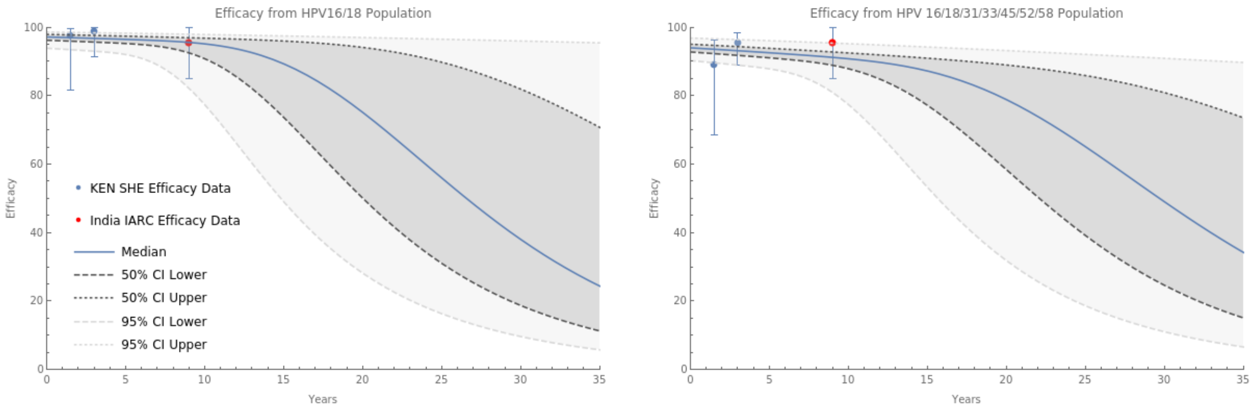


*Figure 7: Efficacy plots resulting from each of the alternative-case posterior distributions (KEN SHE + India IARC). The blue line is the efficacy assuming the median parameter values. The gray regions represent the uncertainty in the efficacy estimate over time.*

When we include the India IARC data we see, as expected, longer estimated median duration of protection of 20 to 25 years, and >95% probability of being longer than about 10 to 11 years compared to about 4 years for the KEN SHE only. The degree of protection factor for HPV 6/11/16/18 distribution remains the same but with a narrower confidence interval. The degree of protection factor for the HPV 31/33/45/52/58 distribution increased by about 1% also with a narrower confidence interval.

The impact of having a longer or shorter duration needs be considered in the context of the age of vaccination and age of peak sexual activity (highest risk of infection). If we are vaccinating 9-14 year-olds, then having a median duration of protection of 9 years implies that about half the vaccinated population will lose protection between ages 18 and 23 years, just as they are reaching their peak sexually active years. On the other hand, if the duration of protection is 20 years, then about half the population is losing protection between ages 29 and 34 years, which is generally after peak sexual activity ages.

# Appendix C: Model parameters

## C.1. Natural History Parameters

Values of these parameters were obtained from the literature, estimated by calibration process, or were assumed.

*Table 7. Rate of cancer progression (all cancers)*

| Progression | Rate |
|---|---|
| Local to regional | 0.1 |
| Regional to distant | 0.3 |

Source: Myers[12] and Sanders[13]

*Table 8. Recurrence rate of treated CIN by stage*

| Stage | Rate |
|---|---|
| CIN 1 | 0.05 |
| CIN 2 | 0.05 |
| CIN 3 | 0.05 |

Source: MSD calibrated model

*Table 9: CIN progression and regression*

| % Progressing from persistently infected with HPV to CIN | |
|---|---|
| CIN 1 | 9.4 |
| CIN 2 | 5.8 |
| CIN 3 | 3.5 |
| % Regressing from CIN to HPV infected | |
| CIN 1 | 32.9 |
| CIN 2 | 21 |
| CIN 3 | 11 |
| % Progressing through CIN stages | |
| CIN 1 to CIN 2 | 13.3 |
| CIN 2 to CIN 3 | 3 |
| CIN 3 to CIS 1 | 3 |
| % Regressing to CIN stages | |
| CIN 2 to CIN 1 | 13.6 |
| CIN 3 to CIN 1 | 14 |
| CIN 3 to CIN 2 | 43 |

Source: Elbasha et al, 2010[14], De Aloysio 1994[15], Insinga 2007[16]. Note: used HPV16 values for all high-risk types
CIN: cervical intraepithelial neoplasia

*Table 10: Cervical transmission coefficients, fraction of persistent infection, seroconversion and natural protection parameters*

| Parameters | HPV16 | HPV18 | HPV31 | HPV33 | HPV45 | HPV52 | HPV58 |
|---|---|---|---|---|---|---|---|
| Transmission coefficients* | | | | | | | |
| To Male | 0.8928 | 0.2161 | 0.4512 | 0.2894 | 0.2004 | 0.3257 | 0.24 |
| To Female | 0.7395 | 0.3714 | 0.8818 | 0.4866 | 0.4079 | 0.3596 | 0.3529 |
| Fraction of persistent cervical HPV infections* | | | | | | | |
| Female | 0.7922 | 0.7998 | 0.1828 | 0.5577 | 0.4915 | 0.745 | 0.5609 |
| Fraction recovering with seroconversion** | | | | | | | |
| Male | 0.0743 | 0.0743 | 0.0743 | 0.0743 | 0.0743 | 0.0743 | 0.0743 |
| Female | 0.7928 | 0.7928 | 0.7928 | 0.7928 | 0.7928 | 0.7928 | 0.7928 |
| Degree of protection with seroconversion** | | | | | | | |
| Male | 0.3295 | 0.3295 | 0.3295 | 0.3295 | 0.3295 | 0.3295 | 0.3295 |
| Female | 0.4998 | 0.4998 | 0.4998 | 0.4998 | 0.4998 | 0.4998 | 0.4998 |
| Degree of protection without seroconversion*** | | | | | | | |
| Male | 0.0000 | 0.0000 | 0.0000 | 0.0000 | 0.0000 | 0.0000 | 0.0000 |
| Female | 0.0000 | 0.0000 | 0.0000 | 0.0000 | 0.0000 | 0.0000 | 0.0000 |

* From model calibration
** From US model calibration[17]
***Assumption

*Table 11: VAIN progression and regression rates*

| | |
|---|---|
| % Progressing from persistently infected with HPV to VAIN | |
| VAIN 1 | 0.8 |
| % Regressing from VAIN to HPV infected | |
| VAIN 1 | 40 |
| VAIN 2/3 | 20 |
| % Progressing through VAIN stages | |
| VAIN 1 to VAIN 2/4 | 37 |
| VAIN 2/3 to CIS 1 | 9.5 |

VAIN: vaginal intraepithelial neoplasia

*Table 12: Vaginal transmission coefficients, fraction of persistent infection, seroconversion and natural protection parameters*

| Parameters | HPV16 | HPV18 | HPV31 | HPV33 | HPV45 | HPV52 | HPV58 |
|---|---|---|---|---|---|---|---|
| Transmission coefficients* | | | | | | | |
| To Male | 0.3432 | 0.2372 | 0.2264 | 0.2811 | 0.1634 | 0.1708 | 0.2784 |
| To Female | 0.5951 | 0.3479 | 0.2222 | 0.238 | 0.3147 | 0.2699 | 0.2625 |
| Fraction of persistent vaginal HPV infections* | | | | | | | |
| Female | 0.1234 | 0.0549 | 0.1766 | 0.0891 | 0.1529 | 0.1898 | 0.0638 |
| Fraction recovering with seroconversion** | | | | | | | |
| Male | 0.0743 | 0.0743 | 0.0743 | 0.0743 | 0.0743 | 0.0743 | 0.0743 |
| Female | 0.7928 | 0.7928 | 0.7928 | 0.7928 | 0.7928 | 0.7928 | 0.7928 |
| Degree of protection with seroconversion** | | | | | | | |
| Male | 0.3295 | 0.3295 | 0.3295 | 0.3295 | 0.3295 | 0.3295 | 0.3295 |
| Female | 0.4998 | 0.4998 | 0.4998 | 0.4998 | 0.4998 | 0.4998 | 0.4998 |
| Degree of protection without seroconversion*** | | | | | | | |
| Male | 0.0000 | 0.0000 | 0.0000 | 0.0000 | 0.0000 | 0.0000 | 0.0000 |
| Female | 0.0000 | 0.0000 | 0.0000 | 0.0000 | 0.0000 | 0.0000 | 0.0000 |

* From model calibration

** From US model calibration[17]

***Assumption

*Table 13: Vulvar disease progression and regression rates*

| | |
|---|---|
| % Progressing from persistently infected with HPV to VIN | |
| VIN 1 | 1.6 |
| % Regressing from VIN to HPV infected | |
| VIN 1 | 40 |
| VIN 2/3 | 20 |
| % Progressing through VIN stages | |
| VIN 1 to VIN 2/4 | 36 |
| VIN 2/3 to CIS 1 | 5 |

VIN: vulvar intraepithelial neoplasia

*Table 14: Vulvar transmission coefficients, fraction of persistent infection, seroconversion and natural protection parameters*

| Parameters | HPV16 | HPV18 | HPV31 | HPV33 | HPV45 | HPV52 | HPV58 |
|---|---|---|---|---|---|---|---|
| Transmission coefficients* | | | | | | | |
| To Male | 0.509 | 0.4387 | 0.2956 | 0.2864 | 0.2407 | 0.1669 | 0.3439 |
| To Female | 0.5675 | 0.492 | 0.2911 | 0.2334 | 0.2861 | 0.3006 | 0.2358 |
| Fraction of persistent vulvar HPV infections* | | | | | | | |
| Female | 0.2338 | 0.0275 | 0.0378 | 0.1838 | 0.1107 | 0.1665 | 0.0388 |

| Fraction recovering with seroconversion** | | | | | | | |
|---|---|---|---|---|---|---|---|
| Male | 0.0743 | 0.0743 | 0.0743 | 0.0743 | 0.0743 | 0.0743 | 0.0743 |
| Female | 0.7928 | 0.7928 | 0.7928 | 0.7928 | 0.7928 | 0.7928 | 0.7928 |
| Degree of protection with seroconversion** | | | | | | | |
| Male | 0.3295 | 0.3295 | 0.3295 | 0.3295 | 0.3295 | 0.3295 | 0.3295 |
| Female | 0.4998 | 0.4998 | 0.4998 | 0.4998 | 0.4998 | 0.4998 | 0.4998 |
| Degree of protection without seroconversion*** | | | | | | | |
| Male | 0.0000 | 0.0000 | 0.0000 | 0.0000 | 0.0000 | 0.0000 | 0.0000 |
| Female | 0.0000 | 0.0000 | 0.0000 | 0.0000 | 0.0000 | 0.0000 | 0.0000 |

* From model calibration
** From US model calibration[17]
***Assumption

*Table 15: Anal HPV progression to AIN and from AIN2/3 to cancer*

| | HPV16 | HPV18 | HPV31 | HPV33 | HPV45 | HPV52 | HPV58 |
|---|---|---|---|---|---|---|---|
| % progressing from persistently infected with HPV to AIN* | | | | | | | |
| Male | 7.0 | 5.4 | 3.0 | 2.0 | 1.0 | 1.3 | 2.8 |
| Female | 1.9 | 0.8 | 1.1 | 0.6 | 1.2 | 0.3 | 1.4 |
| Progression rate from CIS1 to CIS2* | | | | | | | |
| Male | 0.055 | 0.049 | 0.05 | 0.05 | 0.06 | 0.05 | 0.055 |
| Female | 0.061 | 0.046 | 0.048 | 0.046 | 0.047 | 0.047 | 0.061 |
| Progression rate from CIS2 to local cancer* | | | | | | | |
| Male | 0.046 | 0.094 | 0.086 | 0.072 | 0.052 | 0.071 | 0.058 |
| Female | 0.046 | 0.047 | 0.048 | 0.046 | 0.063 | 0.047 | 0.058 |

*from model calibration

*Table 16: AIN progression and regression*

| | Male | Female |
|---|---|---|
| % Regressing from AIN to HPV infected | | |
| AIN 1 | 40 | 40 |
| AIN 2/3 | 20 | 20 |
| % Progressing through AIN stages | | |
| AIN 1 to AIN 2/4 | 18 | 18 |
| AIN 2/3 to CIS 1 | 19.1 | 25.9 |

AIN: anal intraepithelial neoplasia

*Table 17: Anal transmission coefficients, fraction of persistent infection, seroconversion and natural protection parameters*

| Parameters | HPV16 | HPV18 | HPV31 | HPV33 | HPV45 | HPV52 | HPV58 |
|---|---|---|---|---|---|---|---|
| Transmission coefficients* | | | | | | | |
| To Male | 0.2007 | 0.2761 | 0.2493 | 0.2638 | 0.3239 | 0.263 | 0.2722 |
| To Female | 0.249 | 0.254 | 0.258 | 0.2908 | 0.2003 | 0.2611 | 0.2354 |
| Fraction of persistent anal HPV infections* | | | | | | | |
| Male | 0.3000 | 0.0295 | 0.0451 | 0.0449 | 0.0593 | 0.0365 | 0.0478 |
| Female | 0.2394 | 0.0758 | 0.0427 | 0.0615 | 0.0165 | 0.0305 | 0.0364 |
| Fraction recovering with seroconversion** | | | | | | | |
| Male | 0.2500 | 0.2500 | 0.2500 | 0.2500 | 0.2500 | 0.2500 | 0.2500 |
| Female | 0.6000 | 0.6000 | 0.6000 | 0.6000 | 0.6000 | 0.6000 | 0.6000 |
| Degree of protection with seroconversion** | | | | | | | |
| Male | 0.0496 | 0.0496 | 0.0496 | 0.0496 | 0.0496 | 0.0496 | 0.0496 |
| Female | 0.3938 | 0.3938 | 0.3938 | 0.3938 | 0.3938 | 0.3938 | 0.3938 |
| Degree of protection without seroconversion*** | | | | | | | |
| Male | 0.0000 | 0.0000 | 0.0000 | 0.0000 | 0.0000 | 0.0000 | 0.0000 |
| Female | 0.0000 | 0.0000 | 0.0000 | 0.0000 | 0.0000 | 0.0000 | 0.0000 |

* From model calibration

** From US model calibration[17]

***Assumption

*Table 18: oropharyngeal HPV infection progression to OIN and from OIN2/3 to cancer*

| | HPV16 | HPV18 | HPV33 | HPV45 | HPV58 |
|---|---|---|---|---|---|
| % Progressing from persistently infected with HPV to OIN* | | | | | |
| Male | 5.0 | 1.2 | 3.1 | 1.3 | 1.7 |
| Female | 5.5 | 2.5 | 1.5 | 1.0 | 9.9 |
| Progression rate from CIS1 to CIS2* | | | | | |
| Male | 0.0462 | 0.0988 | 0.0516 | 0.099 | 0.061 |
| Female | 0.0462 | 0.0988 | 0.0516 | 0.099 | 0.061 |
| Progression rate from CIS2 to local cancer* | | | | | |
| Male | 0.0462 | 0.0482 | 0.0957 | 0.0875 | 0.0651 |
| Female | 0.099 | 0.0561 | 0.0858 | 0.0488 | 0.0476 |

* from model calibration

OIN: oropharyngeal intraepithelial neoplasia

*Table 19: OIN progression and regression*

| | Male | Female |
|---|---|---|
| % Regressing from OIN to HPV infected | | |
| OIN 1 | 10.5 | 10.5 |

| | | |
|---|---|---|
| OIN 2/3 | 0 | 0 |
| % Progressing through OIN stages | | |
| OIN 1 to OIN 2/4 | 18 | 18 |
| OIN 2/3 to CIS 1 | 17.3 | 17.3 |

OIN: oropharyngeal intraepithelial neoplasia

*Table 20: Oropharyngeal transmission coefficients, fraction of persistent infection, seroconversion and natural protection parameters*

| **Parameters** | **HPV16** | **HPV18** | **HPV33** | **HPV45** | **HPV58** |
|---|---|---|---|---|---|
| Transmission coefficients* | | | | | |
| To Male | 0.3486 | 0.3538 | 0.3136 | 0.3205 | 0.3231 |
| To Female | 0.2698 | 0.2413 | 0.2422 | 0.2538 | 0.251 |
| Fraction of persistent anal HPV infections* | | | | | |
| Male | 0.0186 | 0.0032 | 0.0064 | 0.001 | 0.0015 |
| Female | 0.0108 | 0.0017 | 0.0061 | 0.001 | 0.0007 |
| Fraction recovering with seroconversion** | | | | | |
| Male | 0.1040 | 0.1000 | 0.1000 | 0.1000 | 0.1000 |
| Female | 0.6048 | 0.6000 | 0.6000 | 0.6000 | 0.6000 |
| Degree of protection with seroconversion** | | | | | |
| Male | 0.1159 | 0.0496 | 0.0496 | 0.0496 | 0.0496 |
| Female | 0.2012 | 0.3938 | 0.3938 | 0.3938 | 0.3938 |
| Degree of protection without seroconversion*** | | | | | |
| Male | 0.0000 | 0.0000 | 0.0000 | 0.0000 | 0.0000 |
| Female | 0.0000 | 0.0000 | 0.0000 | 0.0000 | 0.0000 |

* From model calibration
** From US model calibration[17]
***Assumption

*Table 21: PIN progression and regression*

| | |
|---|---|
| % Progressing from persistently infected with HPV to PIN | |
| PIN 1 | 2.1 |
| % Regressing from PIN to HPV infected | |
| PIN 1 | 40 |
| PIN 2/3 | 20 |
| % Progressing through PIN stages | |
| PIN 1 to PIN 2/4 | 18 |
| PIN 2/3 to CIS 1 | 17.3 |

PIN: penile intraepithelial neoplasia

*Table 22: Penile transmission coefficients, fraction of persistent infection, seroconversion and natural protection parameters*

| Parameters | HPV16 | HPV18 | HPV31 | HPV33 | HPV45 | HPV52 | HPV58 |
|---|---|---|---|---|---|---|---|
| Transmission coefficients* | | | | | | | |
| To Male | 0.6876 | 0.1785 | 0.2154 | 0.2097 | 0.4632 | 0.5747 | 0.5747 |
| To Female | 0.6978 | 0.5704 | 0.3915 | 0.4268 | 0.3384 | 0.1935 | 0.1935 |
| Fraction of persistent anal HPV infections* | | | | | | | |
| Male | 0.0976 | 0.0234 | 0.0273 | 0.0431 | 0.0096 | 0.0064 | 0.0064 |
| Fraction recovering with seroconversion** | | | | | | | |
| Male | 0.0743 | 0.0743 | 0.0743 | 0.0743 | 0.0743 | 0.0743 | 0.0743 |
| Female | 0.7928 | 0.7928 | 0.7928 | 0.7928 | 0.7928 | 0.7928 | 0.7928 |
| Degree of protection with seroconversion** | | | | | | | |
| Male | 0.3295 | 0.3295 | 0.3295 | 0.3295 | 0.3295 | 0.3295 | 0.3295 |
| Female | 0.4998 | 0.4998 | 0.4998 | 0.4998 | 0.4998 | 0.4998 | 0.4998 |
| Degree of protection without seroconversion*** | | | | | | | |
| Male | 0.0000 | 0.0000 | 0.0000 | 0.0000 | 0.0000 | 0.0000 | 0.0000 |
| Female | 0.0000 | 0.0000 | 0.0000 | 0.0000 | 0.0000 | 0.0000 | 0.0000 |

* From model calibration
** From US model calibration[17]
***Assumption

## C.2. Treatment Patterns

*Table 23: Cervical disease and cancer % cured and % treated*

| Disease | % Cured | % Treated |
|---|---|---|
| CIN 1 | 96[18] | 0 |
| CIN 2+ | 92[18] | 80 |
| Local Cancer | 92[19] | 100 |
| Regional Cancer | 57[19] | 100 |
| Distant Cancer | 17[19] | 100 |

Sources: Treated CIN from local expert opinion.
Cancer treated is assumed

*Table 24: Cervical disease symptom recognition*

| | HPV16 | HPV18 | HPV31 | HPV33 | HPV45 | HPV52 | HPV58 |
|---|---|---|---|---|---|---|---|

| | | | | | | | | |
|---|---|---|---|---|---|---|---|---|
| % Recognizing symptoms by disease* | | | | | | | | |
| CIN 1 | 3 | 3 | 9 | 25 | 2 | | 26 | 11 |
| CIN 2+ | 11 | 1 | 19 | 16 | 2 | | 6 | 9 |
| Local Cancer | 1 | 1 | 1 | 1 | 1 | | 1 | 1 |
| Regional Cancer | 1 | 1 | 1 | 1 | 1 | | 1 | 1 |
| Distant Cancer | 10 | 10 | 10 | 10 | 10 | | 10 | 10 |

*From calibration

*Table 25: Percent of vaginal cancer patients recognizing symptoms and seeking treatment.*

| | HPV16 | HPV18 | HPV31 | HPV33 | HPV45 | HPV52 | HPV58 |
|---|---|---|---|---|---|---|---|
| % Recognizing symptoms by disease* | | | | | | | |
| Local Cancer | 0 | 0 | 0 | 0 | 0 | 0 | 0 |
| Regional Cancer | 16 | 16 | 16 | 16 | 16 | 16 | 16 |
| Distant Cancer | 50 | 50 | 50 | 50 | 50 | 50 | 50 |

*From calibration

*Table 26: Percent of vulvar cancer patients recognizing symptoms and seeking treatment*

| | HPV16 | HPV18 | HPV31 | HPV33 | HPV45 | HPV52 | HPV58 |
|---|---|---|---|---|---|---|---|
| % Recognizing symptoms by disease* | | | | | | | |
| Local Cancer | 0 | 0 | 0 | 0 | 0 | 0 | 0 |
| Regional Cancer | 20 | 20 | 20 | 20 | 20 | 20 | 20 |
| Distant Cancer | 51 | 51 | 51 | 51 | 51 | 51 | 51 |

*From calibration

*Table 27: Percent of anal cancer patients recognizing symptoms and seeking treatment. Same values for male and female*

| | HPV16 | HPV18 | HPV31 | HPV33 | HPV45 | HPV52 | HPV58 |
|---|---|---|---|---|---|---|---|
| % Recognizing symptoms by disease* | | | | | | | |
| Local Cancer | 0 | 0 | 0 | 0 | 0 | 0 | 0 |
| Regional Cancer | 3 | 3 | 3 | 3 | 3 | 3 | 3 |
| Distant Cancer | 10 | 10 | 10 | 10 | 10 | 10 | 10 |

*From calibration

*Table 28: Percent of oropharyngeal patients recognizing symptoms and seeking treatment.*

| | | HPV16 | HPV18 | HPV31 | HPV33 | HPV45 | HPV52 | HPV58 |
|---|---|---|---|---|---|---|---|---|
| % Recognizing symptoms by disease* | | | | | | | | |
| Male | Local Cancer | 0 | 0 | N/A | 0 | 0 | N/A | 0 |

| | | | | | | | | |
|---|---|---|---|---|---|---|---|---|
| | Regional Cancer | 5 | 5 | N/A | 5 | 5 | N/A | 5 |
| | Distant Cancer | 89 | 49 | N/A | 66 | 51 | N/A | 60 |
| | Local Cancer | 0 | 0 | N/A | 0 | 0 | N/A | 0 |
| | Regional Cancer | 2 | 2 | N/A | 2 | 2 | N/A | 2 |
| Female | Distant Cancer | 20 | 10 | N/A | 33 | 40 | N/A | 9 |

*From calibration

*Table 29: Percent of penile patients recognizing symptoms and seeking treatment*

| | HPV16 | HPV18 | HPV31 | HPV33 | HPV45 | HPV52 | HPV58 |
|---|---|---|---|---|---|---|---|
| % Recognizing symptoms by disease* | | | | | | | |
| Local Cancer | 0 | 0 | 0 | 0 | 0 | 0 | 0 |
| Regional Cancer | 20 | 20 | 20 | 20 | 20 | 20 | 20 |
| Distant Cancer | 50 | 50 | 50 | 50 | 50 | 50 | 50 |

*From calibration

## C.3. Cancer fatality rate

We estimate the annual probability of death due to cancer from the IARC cancer database (https://gco.iarc.fr/today/online-analysis-table?v=2020&mode=cancer&mode_population=countries&population=900&populations=360&key=asr) as [probability of death from cancer] = [crude death rate]/[crude cancer incidence] using all ages. Data and final rates are shown in Table 30. We assume all stages and ages > 15 have the same probability of death.

*Table 30: Cancer fatality rate input data and estimates*

| Cancer | Female | | | Male | | |
|---|---|---|---|---|---|---|
| | Crude Death Rate | Crude Incidence Rate | Probability of Death | Crude Death Rate | Crude Incidence Rate | Probability of Death |
| **Cervical** | 15.5 | 27 | 0.57 | - | - | - |
| **Vaginal** | 0.16 | 0.35 | 0.12 | - | - | - |
| **Vulvar** | 0.33 | 0.98 | 0.47 | - | - | - |
| **Penile** | - | - | - | 0.25 | 0.74 | 0.71 |
| **Anal** | 4.8 | 9.1 | 0.53 | 8.4 | 15.8 | 0.53 |
| **Oropharyngeal** | 0.12 | 1.5 | 0.47 | 1.6 | 2.6 | 0.62 |

## C.4. Vaccine Model Parameters

Table 31 shows the vaccine model parameters used for full-series vaccination. Under the assumption of no waning, we assume that the model degree of protection parameters are equivalent to the study efficacy results. We assume that protection against vaginal and vulvar end-points are the same as for

cervical protection. We assume that protection against head and neck end-points are the same as anal protection. When there is no male data available, we assume the same protection as for females. Similarly, when there is no female data, we assume the same protection as for males.

*Table 31: Vaccine model properties for full-series.*

| Vaccine assumptions | HPV 16 | HPV 18 | HPV 31 | HPV 33 | HPV45 | HPV 52 | HPV 58 |
|---|---|---|---|---|---|---|---|
| *Cervical, vaginal, and vulvar models* | | | | | | | |
| Degree of protection against HPV infections by type | | | | | | | |
| Male | 0.411[20] | 0.621[20] | 0.411[a] | 0.411[a] | 0.621[a] | 0.411[a] | 0.411[a] |
| Female | 0.760[b] | 0.963[b] | 0.760[a] | 0.760[a] | 0..963[a] | 0.760[a] | 0.760[a] |
| Degree of protection of the vaccine against cervical HPV infections becoming persistent by type | | | | | | | |
| Female | 0.988[b] | 0.984[b] | 0.960[20] | 0.987[20] | 0.973[20] | 0.972[20] | 0.953[20] |
| Degree of protection of the vaccine against HPV-related CIN by type | | | | | | | |
| Female | 1.0[21] | 1.0[21] | 0.944[22] | 1.00[22] | 0.904[22] | 1.0[22] | 1.0[22] |
| *Anal model* | | | | | | | |
| Vaccine efficacy for preventing anal HPV infections by type in the anal model | | | | | | | |
| Male | 0.762[23] | 1.0[23] | 0.762[a] | 0.762[a] | 1.0[a] | 0.762[a] | 0.762[a] |
| Female | 0.762[23] | 1.0[23] | 0.762[c] | 0.762[c] | 1.0[c] | 0.762[c] | 0.762[c] |
| Degree of protection of the vaccine against anal HPV infections becoming persistent by type | | | | | | | |
| Male/ Female | 0.938[23] | 1.0[23] | 0.938[c] | 0.938[c] | 1.0[c] | 0.938[c] | 0.938[c] |
| Degree of protection of the vaccine against HPV-related AIN neoplasia by type [c] | | | | | | | |
| Male/Female | 0.0[23] | 0.0[23] | 0.0[w] | 0.0[w] | 0.0[w] | 0.0[w] | 0.0[w] |
| *Penile model* | | | | | | | |
| Vaccine efficacy for preventing HPV infections by type in the penile model | | | | | | | |
| Male/Female | 0.762[23] | 1.0[23] | 0.762[a] | 0.762[a] | 1.0[a] | 0.762[a] | 0.762[a] |
| Degree of protection of the vaccine against penile HPV infections becoming persistent by type | | | | | | | |
| Male | 0.938[24] | 1.0[24] | 0.938[a] | 0.938[a] | 1.0[a] | 0.938[a] | 0.938[a] |
| Degree of protection of the vaccine against HPV-related PIN neoplasia by type | | | | | | | |
| Male | 0.0 | 0.0 | 0.0 | 0.0 | 0.0 | 0.0 | 0.0 |
| *H & N model[d]* | | | | | | | |
| Vaccine efficacy for preventing HPV infections by type in the H&N model | | | | | | | |
| Male/Female | 0.762[23] | 1.0[23] | 0.762[a] | 0.762[a] | 1.0[a] | 0.762[a] | 0.762[a] |
| Degree of protection of the vaccine against H & N HPV infections becoming persistent by type | | | | | | | |
| Male/Female | 0.938[23] | 1.0[23] | 0.938[c] | 0.938[c] | 1.0[c] | 0.938[c] | 0.938[c] |
| Degree of protection of the vaccine against HPV-related H & N neoplasia by type | | | | | | | |
| Male/Female | 0.0[23] | 0.0[23] | 0.0[a] | 0.0[a] | 0.0[a] | 0.0[a] | 0.0[a] |

Preventing male genital infections through male vaccination is assumed to prevent transmission of genital infections to female persons. Preventing female genital infections through vaccination is assumed to prevent transmission of genital infections to male persons.

Abbreviations: AIN, anal intraepithelial neoplasia; CIN, cervical intraepithelial neoplasia; H & N, head and neck; HPV, human papillomavirus; PIN, penile intraepithelial neoplasia; VaIN/VIN, vaginal/vulvar intraepithelial neoplasia
Notes: [a] Data unavailable for HPV 31, 33, 52, and 58 assume HPV 16 efficacy, HPV 45 assume HPV 18 efficacy. Efficacy was assumed to be the lowest value between HPV 16 and 18; [b] Data on file (2019), Merck; [c] Lacking female data assumed same as male. [d] When no oral infection-specific data were available, the model assumed the same efficacies as for anal/penile.

# Appendix D: Demographic Parameters

We used the Indonesia population size for 2019 from the United Nations Population Division's World Population Prospects[25][1] database of 137,717,861 males and 135,805,760 females.

Model population is divided into 21 age groups (shown in Table 32) and 3 sexual activity groups (described in Appendix B, below). The population is assumed to be constant size with a fixed age group distribution of the 100-year time horizon.

*Table 32: Model age group structure*

| Model Age Groups | | | | | | | | | | | |
|---|---|---|---|---|---|---|---|---|---|---|---|
| | 0 | 1-7 | 8 | 9-14 | 15-18 | 19 | 20-24 | 25-26 | 27-29 | 30-34 | |
| | 35-39 | 40-44 | 45-49 | 50-54 | 55-59 | 60-64 | 65-69 | 70-74 | 75-79 | 80-84 | 85+ |

## D.1. All-Cause Mortality

All-cause mortality is derived from the Global health Observatory data repository life table for Indonesia in 2019. We use age-specific death rate between ages x and x+n and convert to our model age group structure. Table 33 shows the all-cause mortality rate by model age groups.

*Table 33: Model all-cause mortality input parameters death rate*

| Model Age Groups | Male | Female |
|---|---|---|
| 0 | 0.02360160 | 0.01881910 |
| 1-7 | 0.00084218 | 0.00071440 |
| 8 | 0.00068793 | 0.00058356 |
| 9-14 | 0.00048204 | 0.00032384 |

| | | |
|---|---|---|
| 15-18 | 0.00105486 | 0.00052861 |
| 19 | 0.00105486 | 0.00052861 |
| 20-24 | 0.00138476 | 0.00066114 |
| 25-26 | 0.00133710 | 0.00081221 |
| 27-29 | 0.00133710 | 0.00081221 |
| 30-34 | 0.00153806 | 0.00108899 |
| 35-39 | 0.00210906 | 0.00163792 |
| 40-44 | 0.00321682 | 0.00257107 |
| 45-49 | 0.00514541 | 0.00407407 |
| 50-54 | 0.00855376 | 0.00628237 |
| 55-59 | 0.01386420 | 0.00950307 |
| 60-64 | 0.02265470 | 0.01480730 |
| 65-69 | 0.03549860 | 0.02353890 |
| 70-74 | 0.05468640 | 0.03922180 |
| 75-79 | 0.08267950 | 0.06492330 |
| 80-84 | 0.12604100 | 0.10660700 |
| 85+ | 0.22432300 | 0.20389300 |

## D.2. Fertility

Fertility data is used in the RRP model for calculation of vertical transmission of HPV6/11 to newborns. We use the UN Population Prospects database medium variant for 2020-2025 from the file: https://population.un.org/wpp/Download/Files/1_Indicators%20(Standard)/EXCEL_FILES/2_Fertility/WPP2019_FERT_F07_AGE_SPECIFIC_FERTILITY.xlsx

| Age Group | Births/1000 Women |
|---|---|
| **15-19** | 45.030 |
| **20-24** | 114.954 |
| **25-29** | 117.801 |
| **30-34** | 92.294 |
| **35-39** | 52.965 |
| **40-44** | 16.382 |
| **45-49** | 4.654 |

## D.3. Sexual Activity Parameters

### D.3.1. Estimating Number of New Sexual Partners per Year

We developed a model to estimate the average annual number of new sexual partners by age groups, for both female and male populations in Indonesia. The modeling process was separated into two steps. In the first step, we used a log-normal distribution to represent the shape of the age-based distributed frequency of new partners. For calibration purposes, we set the minimum age of the first sexual contact was 13 years, and the age with the highest number of sexual contacts was set to 21 years. The model was then fit to sexual activity data from Spain[26] (Table 34) to estimate the mean and the standard

deviation of the log-normal distribution (Figure 8) resulting in a mean of 3.6 for both males and females, where the mean is the number of years past sexual debut, and standard deviation of 1.3 for both males and females .

*Table 34: Sexual partnerships for males and females in Spain showing the scaled fit to the LogNormal distribution of partnerships*

**Table 1. SPAIN**

| Age group (years) | Males (SPAIN) | Females (SPAIN) | Scaled to fit the distribution | |
|---|---|---|---|---|
| | | | Males (SPAIN) | Females (SPAIN) |
| **13-14** | 0.10752 | 0.09072 | 0.00293604 | 0.005297605 |
| **15-17** | 1.344 | 1.134 | 0.036700504 | 0.066220061 |
| **18** | 2.80 | 1.4 | 0.076459383 | 0.081753162 |
| **19** | 2.8 | 1.4 | 0.076459383 | 0.081753162 |
| **20-24** | 2.8 | 1.4 | 0.076459383 | 0.081753162 |
| **25-26** | 2.8 | 1.4 | 0.076459383 | 0.081753162 |
| **27-29** | 2.8 | 1.4 | 0.076459383 | 0.081753162 |
| **30-34** | 2.8 | 1.1 | 0.076459383 | 0.064234627 |
| **35-39** | 2.8 | 1.1 | 0.076459383 | 0.064234627 |
| **40-44** | 2.2 | 1 | 0.060075229 | 0.058395115 |
| **45-49** | 2.2 | 1 | 0.060075229 | 0.058395115 |
| **50-54** | 2.03 | 0.90 | 0.055454058 | 0.052555604 |
| **55-59** | 2.03 | 0.80 | 0.055454058 | 0.046716092 |
| **60-64** | 1.52 | 0.80 | 0.041590543 | 0.046716092 |
| **65-69** | 1.52 | 0.60 | 0.041590543 | 0.035037069 |
| **70-74** | 1.02 | 0.40 | 0.027727029 | 0.023358046 |
| **75-79** | 1.02 | 0.40 | 0.027727029 | 0.023358046 |
| **80-84** | 1.02 | 0.40 | 0.027727029 | 0.023358046 |
| **>85** | 1.02 | 0.40 | 0.027727029 | 0.023358046 |
| | | | **sum=1** | **sum=1** |

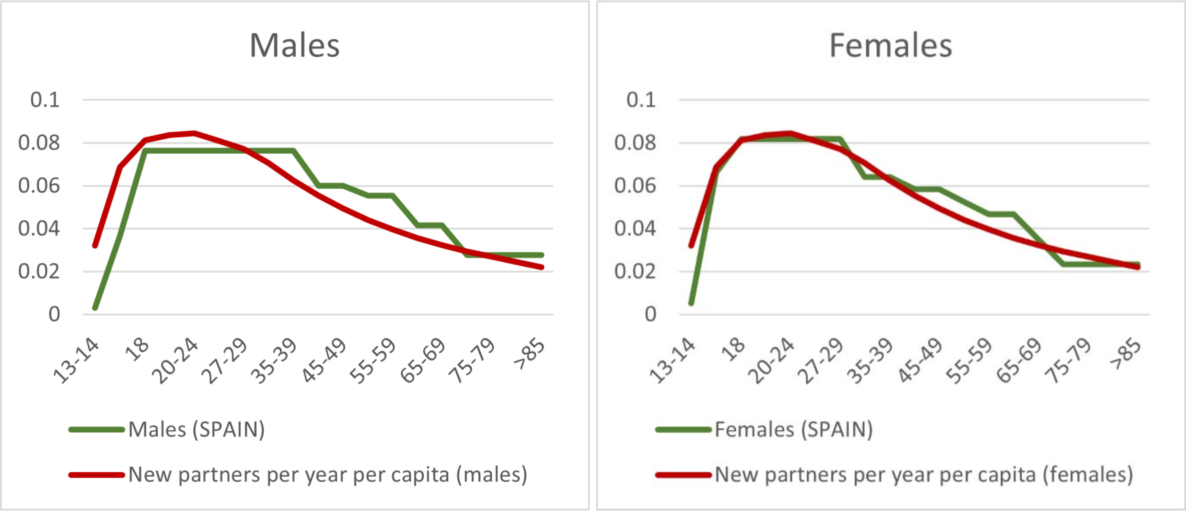


*Figure 8: Fitted LogNormal distribution for male and female sexual partner data in Spain*

In the second step, the established log-normal distribution was further used to estimate and distribute an average number of lifetime sexual partners across the defined age groups. No sexual activity data was available for Indonesia in the literature. Instead, we used data for lifetime sexual partners by age in Thailand as a surrogate[27]. The best fit of the LogNormal distribution was obtained by minimizing the residual of the average (over ages) of lifetime partners and varying by the number of partners that would accumulate by 100 years. The estimated lifetime partners by age group is shown along with the data from Techasrivichien et al, 2016[27] in Figure 9. The final set of parameters for estimating the annual sexual activity by age is presented in Table 35. The resulting age-based distribution of accumulated partners compared to the data is presented in Figure 9. Note that the decreasing number of lifetime partners by age for females is likely the result of cohort effects reflecting changing sexual norms so that younger cohorts will likely have more sexual partners by the time they reach the ages of the current older cohorts. Since we are fitting to an average of over the current cohorts, the estimated sexual activity in females is very likely an underestimate of future sexual activity. Finally, the average number of new sexual partners by model age groups is shown in Table 36.

*Table 35: Sexual activity model parameter inputs*

| | Female | Male |
|---|---|---|
| Age Debut† | 15 | 15 |
| Age Max Activity† | 21 | 21 |
| Mean* | 3.6 | 3.6 |
| STD* | 1.3 | 1.3 |
| Lifetime Partners‡ | 5.9 | 26.6 |

† Estimate for Indonesia
* From fitting Spain data
‡ From minimization

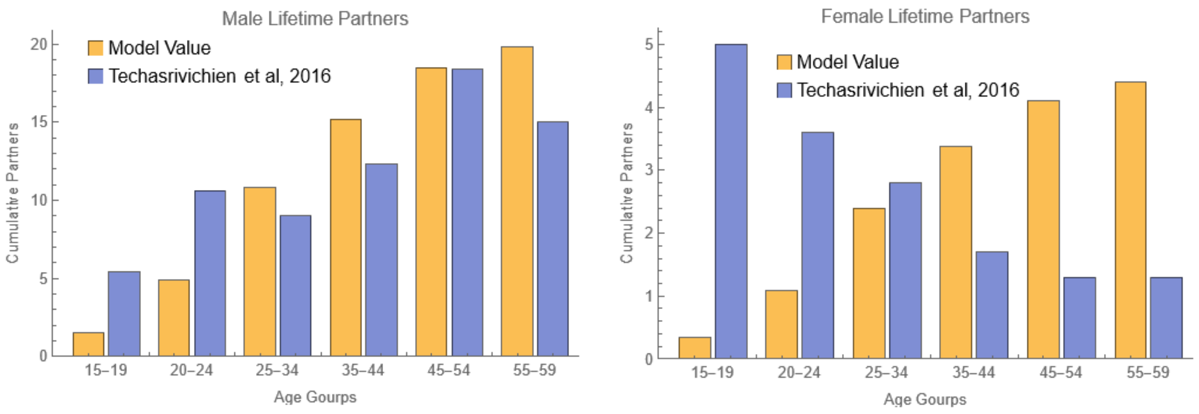


*Figure 9: Fitting to the average number of lifetime sexual partners obtained from Techasrivichien, et al.*[27]

*Table 36: DTM model sexual activity per capita per year derived from sexual activity model*

| Age Group | Male | Female |
|---|---|---|
| 15-18 | 0.379523 | 0.084279 |
| 19 | 0.644922 | 0.143215 |
| 20-24 | 0.676049 | 0.150127 |
| 25-26 | 0.64401 | 0.143012 |
| 27-29 | 0.602309 | 0.133752 |
| 30-34 | 0.534281 | 0.118645 |
| 35-39 | 0.45776 | 0.101652 |
| 40-44 | 0.393787 | 0.087446 |
| 45-49 | 0.341117 | 0.07575 |
| 50-54 | 0.297693 | 0.066107 |
| 55-59 | 0.261655 | 0.058104 |
| 60-64 | 0.231499 | 0.051408 |
| 65-69 | 0.206054 | 0.045757 |
| 70-74 | 0.184409 | 0.040951 |
| 75-79 | 0.165859 | 0.036832 |
| 80-84 | 0.149851 | 0.033277 |

#### D.3.1.1. Other Sexual Activity Parameters

We used data from Thailand[27] for sexual activity since Indonesia specific data was not available. The raw data from Techasrivichien, 2016[27] for lifetime sexual partnership is presented in Table 37. We use this data to partition the population into three sexual activity groups: low activity (0-4 lifetime partners), medium activity (5-9 lifetime partners), and high activity (10+ or more lifetime sexual partners).

*Table 37: Lifetime sexual partnership distribution and percent of population that has ever had sex.*

| Number of lifetime partners | Value Males (percent) | Value Females (percent) |
|---|---|---|
| **1** | 16.60% | 65.80% |
| **2** | 10.90% | 18.70% |
| **3-4** | 21.10% | 10.90% |
| **5-9** | 23.20% | 3.00% |
| **≥10** | 28.20% | 1.60% |
| **Ever had sex** | 84.4% | 78.2% |

The resulting sexual activity group population distribution is shown in Table 38.

*Table 38: Proportion of population in each sexual activity group*

| Activity Group | % of population | |
|---|---|---|
| | Female | Male |
| Low (0-4) | 96 | 57 |
| Medium (5-9) | 2.3 | 20 |
| High (10+) | 1.3 | 24 |

We also use the data from Table 37 to estimate the relative partner factor for each of the sexual activity groups relative to the low activity group. We first estimate mean number of lifetime partners in each group by using a weighted average of the number of partners in each group. For groups spanning multiple numbers of partners (such as 3-4), we assume the population is evenly distributed between the ages in the group. Note our middle group is 5-9, so the weighted average is just the same as the average of the numbers 5 through 9 (7). For the high activity group, we assign a value of 20 since we do not know the upper limit of the range of number of partners. The result is in the last two columns of Table 39.

*Table 39: Mean lifetime partners for each activity group and the derived number of partner scale for activity groups*

| Activity Group | Mean lifetime partners | | Relative partner factor for activity groups | |
|---|---|---|---|---|
| | Female | Male | Female | Male |
| Low (0-4) | 1.2 | 1.7 | 1 | 1 |
| Medium (5-9) | 7 | 7 | 5.8 | 4.1 |
| High (10+) | 20 | 20 | 17 | 12 |

We assume the age of sexual debut is 15 years old.  We also assume that people beyond age 60 are generally not sexually active with people below 60 (referred to as sexual cessation age in earlier descriptions of the model).

The details of how these parameters are used in the calculation of the sexual mixing matrix are described in a previous model publication[14].

# Appendix E: Calibration Inputs and Results

## E.1. Type Attribution

Type attribution is shown in Table 40.

*Table 40: HPV Type attribution for cancers and pre-cancers*

| Disease | HPV16 | HPV18 | HPV31 | HPV33 | HPV45 | HPV52 | HPV58 | Refs/notes |
|---|---|---|---|---|---|---|---|---|
| Cervical Cancer | 60% | 11% | 3% | 3% | 6% | 4% | 4% | de Sanjose[28]* |
| CIN 1 | 9.9% | 2.1% | 3.8% | 9.6% | 0.5%[b] | 18.8% | 3.8% | Zhang[29]† |
| CIN 2 | 30.7% | 0.45%[c] | 8.6% | 12.7 | 0.45%[d] | 14.1% | 3.2% | Zhang[29]† |
| CIN 3 | 43.3% | 0.55% | 10.6% | 3.7% | 0.55% | 11.1% | 4.8% | Zhang[29]† |
| Vaginal Cancer | 59% | 5% | 5% | 5% | 4% | 3% | 4% | Alemany[20]‡ |
| Vulvar Cancer | 68% | 4.6% | 1.3% | 5.9% | 2.9% | 1.8% | 1.1% | Serrano[21]‡ |
| Anal Cancer – Female | 83.4% | 3.6% | 1.8% | 3.1% | 0.7% | 0.3% | 2.0% | Serrano[21]‡ |
| Anal Cancer – Male | 80.7% | 3.6% | 1.9% | 2.7% | 0.9% | 0.7% | 1.8% | Alemany[30] |
| Penile Cancer | 68.7% | 1.5% | 0.8% | 2.4% | 2.7% | 1.2% | 1.3% | Alemany[31]‡ |
| Oropharyngeal Cancer -- Female | 20.7% | 0.45% | 0% | 0.82% | 0.10% | 0% | 0.17% | Castellsague[32]# |
| Oropharyngeal Cancer -- Male | 20.7% | 0.45% | 0% | 0.82% | 0.10% | 0% | 0.17% | Castellsague[32]# |

* HPV type attribution for Asia
† HPV type attribution for eastern China
‡ HPV type attribution for world population
# Separate data for male and female oropharyngeal cancer attribution were not available, we used world-wide data. We used the type distribution over HPV+ oropharyngeal cancers world-wide

## E.2. Cervical Pre-cancer Incidence

The study from Vet et al., 2012[33], provided % positive for CIN 1, 2, or 3 between October 2004 and Jun 2006 (20 months). We combined data from CIN II and CIN III case counts and populations by age to estimate CIN II+. The resulting percentages were then scaled to annual cases per 100,000 by multiplying by 3/5. the final target values are shown in the Table 41.

*Table 41: Cervical CIN per 100,000 women years*

| Age Groups | CIN 1 | CIN 2+ |
|---|---|---|
| 15-19 | 420 | 420 |

---

[b] Mean reported value was 0.0, we used the average of the confidence interval (0.0, 1.0%)
[c] Mean reported value was 0.0, we used the average of the confidence interval (0.0, 0.9%)
[d] Mean reported value was 0.0, we used the average of the confidence interval (0.0, 0.9%)

| | | |
|---|---|---|
| 20-29 | 600 | 658 |
| 30-39 | 960 | 757 |
| 40-49 | 840 | 729 |
| 50-59 | 480 | 392 |
| 60+ | 180 | 1197 |

## E.3. Cancer Incidence and Deaths

Cancer incidence and deaths is from the IARC Global Cancer Observatory for Indonesia (https://gco.iarc.fr/today/online-analysis-table?v=2020&mode=cancer&mode_population=countries&population=900&populations=360&key=asr ) where each age group and sex was queried individually. Data is shown in Table 42 through Table 46. We use larger age groups for the anal cancer data because more granular age groups have sparse data given the small incidence rates (see Table 46).

*Table 42: Female cancer incidence per 100,000 female person years. Oropharyngeal female cancer data uses data for 20-29 age group instead of the two more granular age groups used for the other diseases.*

| | Incidence (per 100,000) | | | |
|---|---|---|---|---|
| **Age Groups** | **Cervical** | **Vaginal** | **Vulvar** | **Oropharyngeal** |
| 15-19 | 0.23 | 0.03 | 0.06 | 0.03 |
| 20-24 | 0.45 | 0.04 | 0.15 | 0.07 |
| 25-29 | 5.1 | 0.08 | 0.17 | 0.08 |
| 30-34 | 12.9 | 0.11 | 0.25 | |
| 35-39 | 25 | 0.12 | 0.5 | 0.25 |
| 40-44 | 39.9 | 0.22 | 0.99 | 0.28 |
| 45-49 | 57.9 | 0.53 | 1.4 | 0.49 |
| 50-54 | 74 | 0.83 | 1.9 | 0.6 |
| 55-59 | 83.7 | 1.2 | 2.7 | 0.93 |
| 60-64 | 88 | 1.6 | 4.2 | 1.4 |
| 65-69 | 83.1 | 1.6 | 5.1 | 1.4 |
| 70+ | 68.2 | 1.3 | 4.6 | 1.1 |

*Table 43: Male cancer incidence per 100,000 male person years*

| **Age Groups** | **Incidence (per 100,000)** | |
|---|---|---|
| | **Penile** | **Oropharyngeal** |
| 15-19 | 0.07 | 0.12 |
| 20-24 | 0.22 | 0.05 |
| 25-29 | 0.14 | 0.14 |
| 30-34 | 0.33 | 0.16 |

| | | |
|---|---|---|
| 35-39 | 0.26 | 0.26 |
| 40-44 | 0.71 | 0.62 |
| 45-49 | 1.10 | 0.93 |
| 50-54 | 1.50 | 1.50 |
| 55-59 | 2.50 | 3.20 |
| 60-64 | 3.20 | 3.20 |
| 65-69 | 4.70 | 5.20 |
| 70+ | 4.70 | 4.60 |

*Table 44: Female cancer death incidence per 100,000 female person years*

| Age Groups | Deaths (per 100,000) | | | |
|---|---|---|---|---|
| | Cervical | Vaginal | Vulvar | Oropharyngeal |
| 15-19 | 0.27 | 0 | 0.04 | 0.05 |
| 20-24 | 0.36 | 0.02 | 0.02 | 0.08 |
| 25-29 | 1.4 | 0.02 | 0.06 | 0.1 |
| 30-34 | 2.4 | 0.02 | 0.11 | 0.12 |
| 35-39 | 7 | 0.08 | 0.14 | 0.25 |
| 40-44 | 15 | 0.14 | 0.34 | 0.6 |
| 45-49 | 25 | 0.27 | 0.38 | 0.81 |
| 50-54 | 37 | 37 | 0.54 | 1.5 |
| 55-59 | 51 | 0.53 | 0.86 | 2.2 |
| 60-64 | 64 | 0.65 | 1.4 | 2.8 |
| 65-69 | 72 | 0.73 | 1.6 | 3.2 |
| 70+ | 70 | 0.72 | 2 | 3.8 |

*Table 45: Male cancer death incidence per 100,000 male person years*

| Age Groups | Incidence (per 100,000) | |
|---|---|---|
| | Penile | Oropharyngeal |
| 15-19 | 0 | 0.03 |
| 20-24 | 0.02 | 0.12 |
| 25-29 | 0.05 | 0.15 |
| 30-34 | 0.09 | 0.17 |
| 35-39 | 0.16 | 0.64 |
| 40-44 | 0.28 | 1.3 |
| 45-49 | 0.29 | 2.2 |
| 50-54 | 0.42 | 3.5 |
| 55-59 | 0.66 | 5.1 |
| 60-64 | 0.93 | 6.8 |
| 65-69 | 1.5 | 8.3 |

| | | |
|---|---|---|
| 70+ | 2 | 10 |

*Table 46: Anal cancer incidence and deaths*

| Age Groups | Incidence (per 100,000) | | Deaths (per 100,000) | |
|---|---|---|---|---|
| | Female | Male | Female | Male |
| 15-19 | 0.0 | 0.0 | 0.0 | 0.0 |
| 20-29 | 0.02 | 0.02 | 0.0 | 0.0 |
| 30-39 | 0.14 | 0.10 | 0.01 | 0.02 |
| 40-49 | 0.18 | 0.33 | 0.06 | 0.13 |
| 40-59 | 0.25 | 0.80 | 0.08 | 0.40 |
| 60-69 | 0.54 | 1.8 | 0.19 | 0.96 |
| 70+ | 2.1 | 3.0 | 1.0 | 2.2 |

## E.4. Cancer fatality rate

We estimate the annual probability of death due to cancer from the IARC cancer database (https://gco.iarc.fr/today/online-analysis-table?v=2020&mode=cancer&mode_population=countries&population=900&populations=360&key=asr) as [probability of death from cancer] = [crude death rate]/[crude cancer incidence] using all ages. Data and final rates are shown in Table 47. We assume all stages and ages > 15 have the same probability of death.

*Table 47: Cancer fatality rate input data and estimates*

| Cancer | Female | | | Male | | |
|---|---|---|---|---|---|---|
| | Crude Death Rate | Crude Incidence Rate | Probability of Death | Crude Death Rate | Crude Incidence Rate | Probability of Death |
| **Cervical** | 15.5 | 27 | 0.57 | - | - | - |
| **Vaginal** | 0.16 | 0.35 | 0.12 | - | - | - |
| **Vulvar** | 0.33 | 0.98 | 0.47 | - | - | - |
| **Penile** | - | - | - | 0.25 | 0.74 | 0.71 |
| **Anal** | 4.8 | 9.1 | 0.53 | 8.4 | 15.8 | 0.53 |
| **Oropharyngeal** | 0.12 | 1.5 | 0.47 | 1.6 | 2.6 | 0.62 |

We then assume this is the unweighted average rate across local, regional, and distant cancers and that local has ½ the rate, and distant has 3/2 this rate

## E.5. Genital Warts Incidence and Prevalence

Incidence data for genital warts is from China population[34] and shown in Table 48.

*Table 48: Genital warts incidence*

| Age Groups | Incidence (per 100,000) | |
|---|---|---|
| | Female | Male |
| 15-19 | 25 | 10 |
| 20-24 | 62 | 39 |
| 25-29 | 55 | 45 |
| 30-34 | 30 | 30 |
| 35-39 | 20 | 22 |
| 40-44 | 15 | 15 |
| 45-49 | 9 | 10 |
| 50-54 | 8 | 10 |
| 55-59 | 5 | 5 |
| 60-64 | 5 | 5 |
| 65-69 | 4 | 6 |
| 70+ | 3 | 5 |

Prevalence is the % positive cases for ages 18-55 from the cases diagnosed at baseline from the prospective study in Wei, et al.[34] and is shown in Table 49

*Table 49: Genital warts prevalence*

| Age Groups | Prevalence (%) | |
|---|---|---|
| | Female | Male |
| 18-55 | 0.21 | 0.09 |

## E.6. Calibration Methods and Results

The HPV model is a collection of models consisting of one model for each of the 7 high risk HPV vaccine types (16, 18, 31, 33, 45,52, and 58) for the cervical, vaginal, vulvar, penile, anal, and oropharyngeal sites and two models for the low-risk HPV types (6, 11) combining genital warts, RRP, and low grade CIN. Each of these 44 models is calibrated separately. The calibration process consists of estimating certain model parameters (depending on the site and type being calibrated) by minimizing an objective function involving the calibration target data and the corresponding model output. For all calibrations we minimize a residual function which is the weighted sum of squared differences between the equilibrium model outcome and the target outcome data. Details for each of the residual function are described in the following sections along with the specific parameters that were estimated from calibration.

### E.6.1. Cervical Model Calibration

The cervical model residual function is given by

$$\sum_a \left(\frac{cancer(a) - cancer(a|\theta)}{Max(cancer(a))}\right)^2 + \sum_a \frac{cin1(a) - cin1(a|\theta)}{Max(cin1(a))}^2 + \sum_a \frac{cin23(a) - cin23(a|\theta)}{Max(cin23(a))}^2 + \sum_a \left(\frac{cancerDeaths(a) - cancerDeaths(a|\theta)}{Max(cancerDeaths(a))}\right)^2$$

were outcome(a) is the outcome incidence per 100,000 for age group "a" from the target data, outcome(a|θ) is the model outcome incidence for age group "a" and parameter set "θ", with "outcome" being cancer, cin1, cin23, or cancer deaths. We divide each difference in outcomes by the maximum for that outcome in the target data to ensure that all data points are the same order of magnitude.

The fitting process allowed the variation of three infection related parameters, female to male transmission probability (βm), male to female transmission probability (βf), and the % of incident infections that will become persistent (prf); two disease progression parameters, rate of progression from CIN1 to CIS2 ($\pi_4$), and rate of progression from CIS2 to local cancer ($\pi_5$). In addition, we varied parameters representing the percentage of CIN1, CIN2+, local, regional, and distant cancer that recognize their symptoms and seek treatment.

*Table 50: Parameter values resulting from fitting the cervical model for each high-risk HPV type*

| **Parameter** | **HPV16** | **HPV18** | **HPV31** | **HPV33** | **HPV45** | **HPV52** | **HPV58** |
|---|---|---|---|---|---|---|---|
| βm | 0.893 | 0.216 | 0.451 | 0.289 | 0.200 | 0.326 | 0.240 |
| βf | 0.740 | 0.371 | 0.882 | 0.487 | 0.408 | 0.360 | 0.353 |
| % of infections that are persistent (prf) | 79.2 | 80.0 | 18.3 | 55.8 | 49.2 | 74.5 | 56.1 |
| Progression from CIS1 to CIS2 ($\pi_4$) | 0.054 | 0.049 | 0.047 | 0.047 | 0.036 | 0.060 | 0.046 |
| Progression from CIS2 to Local Cancer ($\pi_5$) | 0.040 | 0.035 | 0.036 | 0.035 | 0.186 | 0.023 | 0.035 |
| **% cases recognizing symptoms and seeking treatment** | | | | | | | |
| CIN1 | 3 | 3 | 9 | 25 | 2 | 26 | 11 |
| CIN2+ | 11 | 1 | 19 | 16 | 2 | 6 | 9 |
| Local Cancer | 1 | 1 | 1 | 1 | 1 | 1 | 1 |
| Regional Cancer | 1 | 1 | 1 | 1 | 1 | 1 | 1 |
| Distant Cancer | 10 | 10 | 10 | 10 | 10 | 10 | 10 |

Figure 10 through Figure 16 show the resulting cervical model fits to target data for each high-risk HPV type.

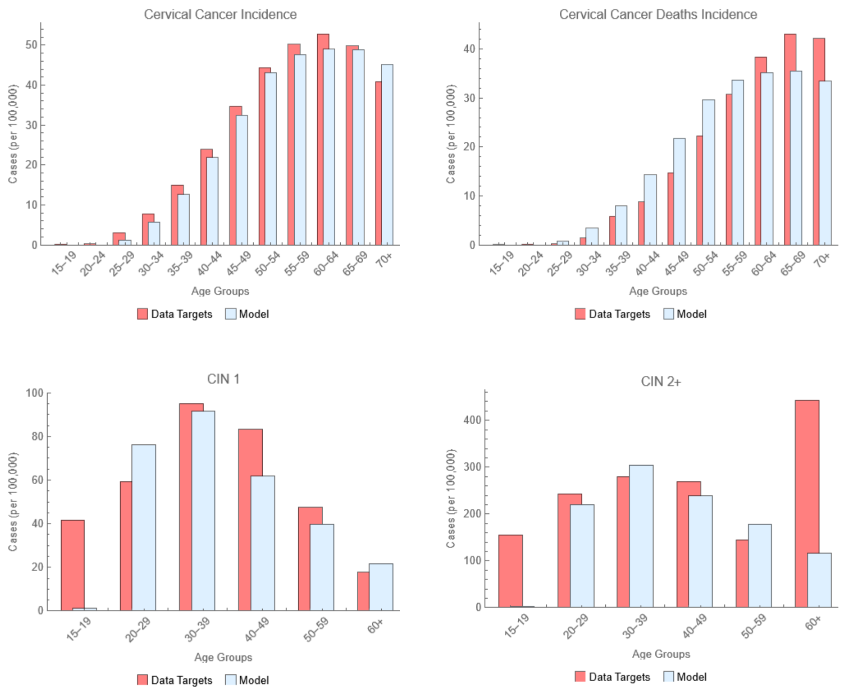


*Figure 10: Cervical HPV 16 model fit to target data*

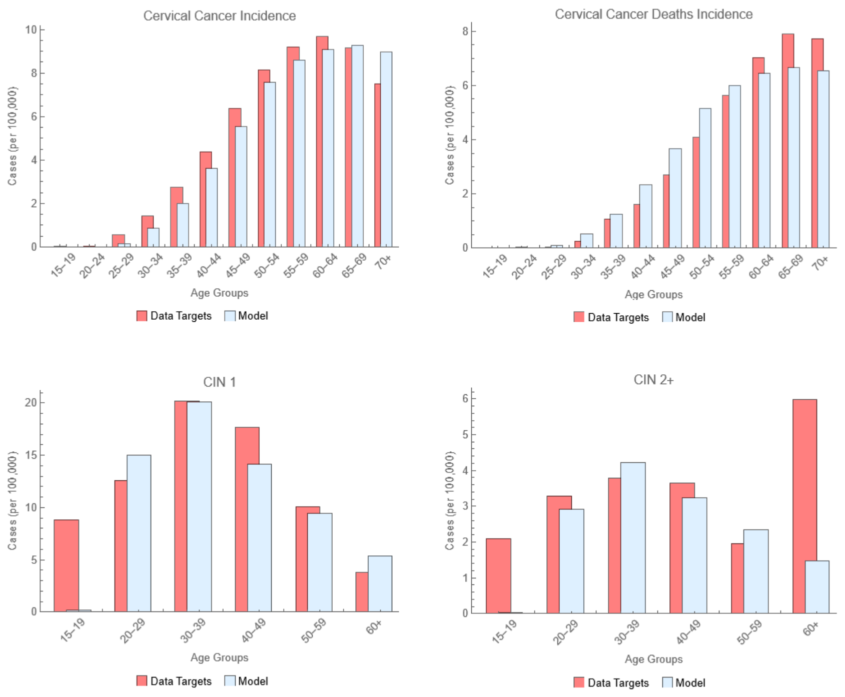


*Figure 11: Cervical HPV 18 model fit to target data*

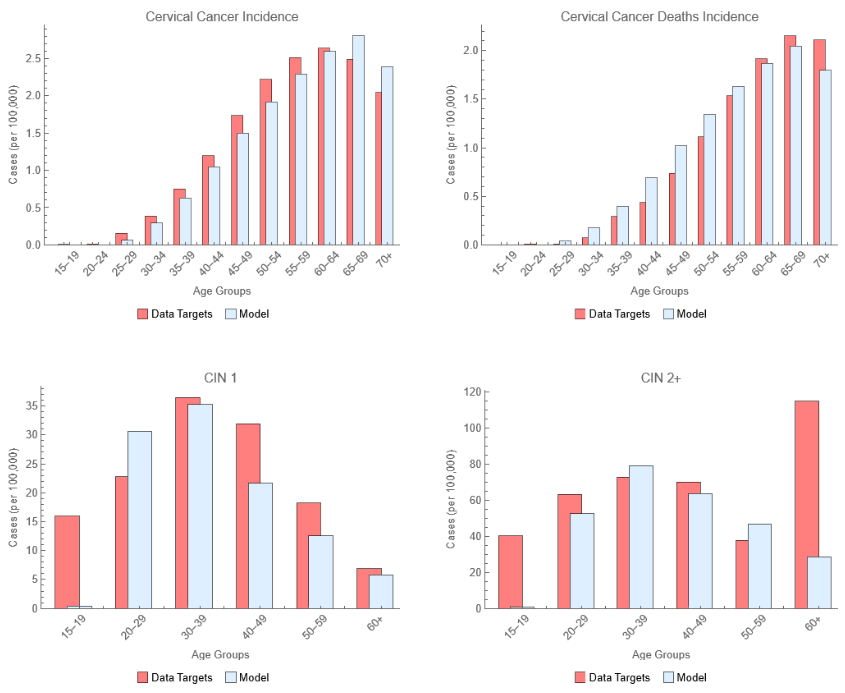


*Figure 12: Cervical HPV 31 model fit to target data*

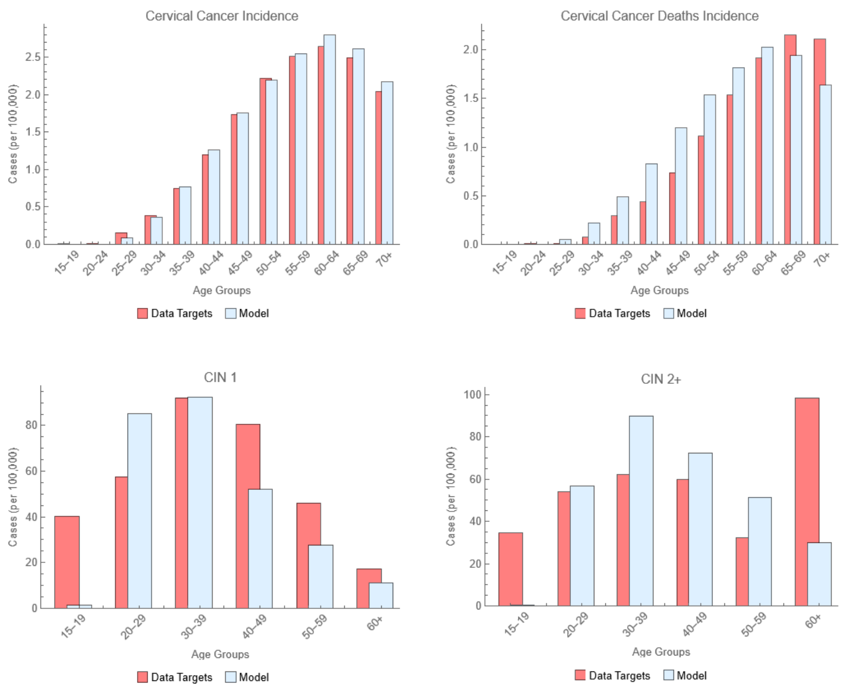


*Figure 13: Cervical HPV 33 model fit to target data*

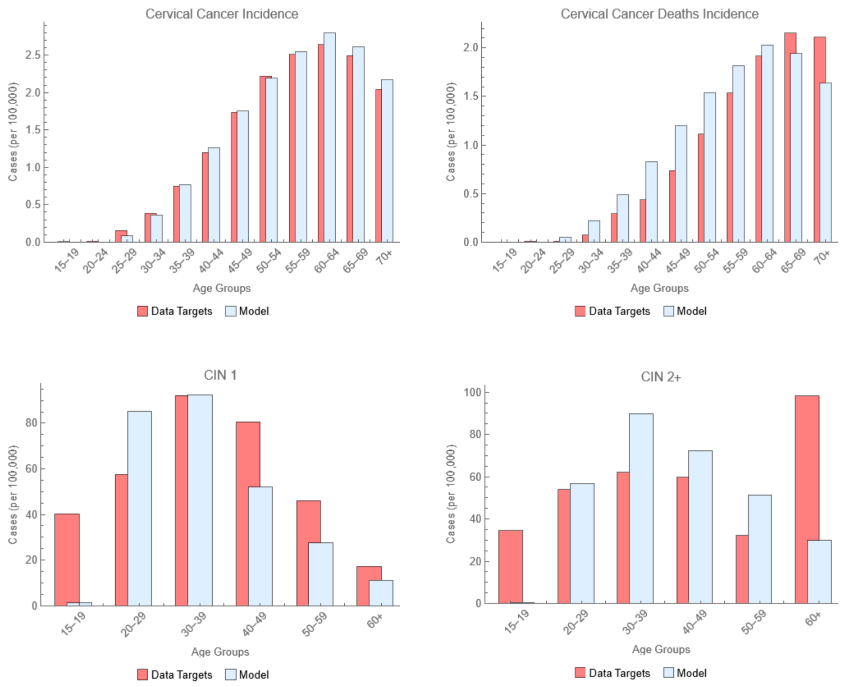


*Figure 14: Cervical HPV 45 model fit to target data*

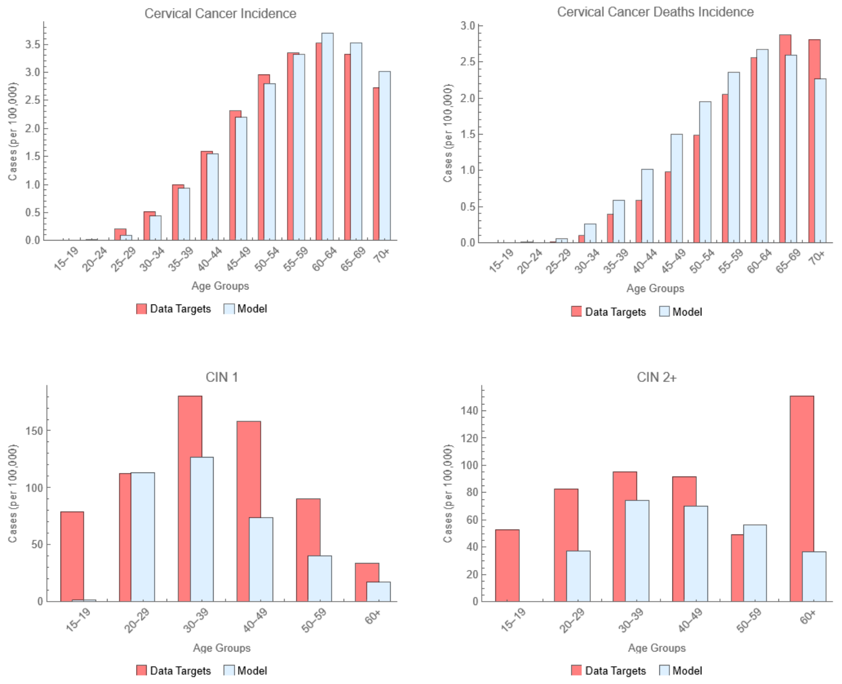


*Figure 15: Cervical HPV 52 model fit to target data*

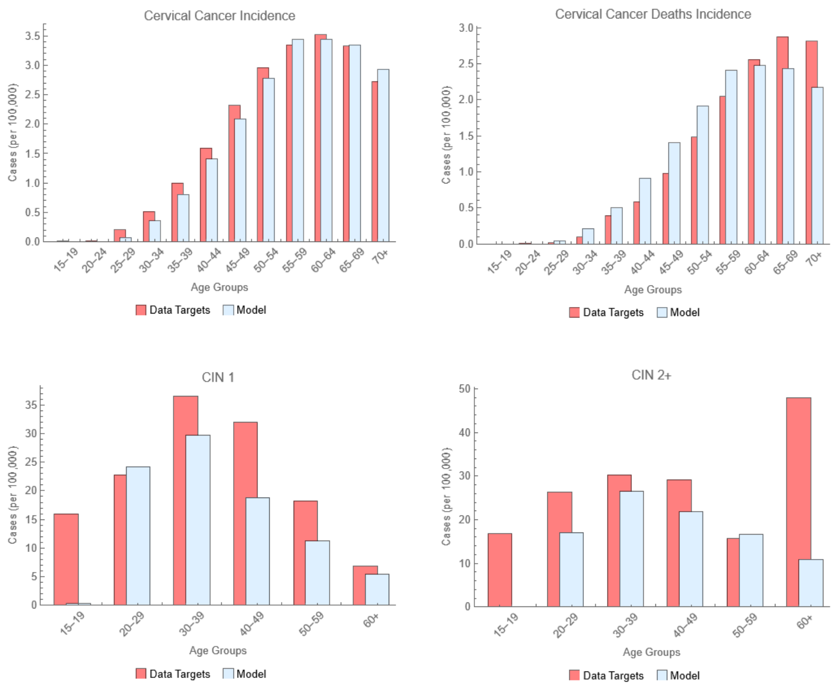


*Figure 16: Cervical HPV 58 model fit to target data*

## E.6.2. Anal Model Calibration

The anal model residual function is

$$\sum_a \left(\frac{cancerMale(a) - cancerMale(a|\theta)}{Max(cancerMale(a))}\right)^2 + \sum_a \left(\frac{cancerFemale(a) - cancerFemale(a|\theta)}{Max(cancerFemale(a))}\right)^2 + \sum_a \left(\frac{cancerDeathsMale(a) - cancerDeathsMale(a|\theta)}{Max(cancerDeathsMale(a))}\right)^2 + \sum_a \left(\frac{cancerDeathsFemale(a) - cancerDeathsFemale(a|\theta)}{Max(cancerDeathsFemale(a))}\right)^2$$

The percent of anal cancers recognizing symptoms and seeking treatment was found from fitting HPV16 and the same values are assumed for each of the other types. Figure 17Figure 23 show the resulting anal model fit to target data for each high-risk HPV type.

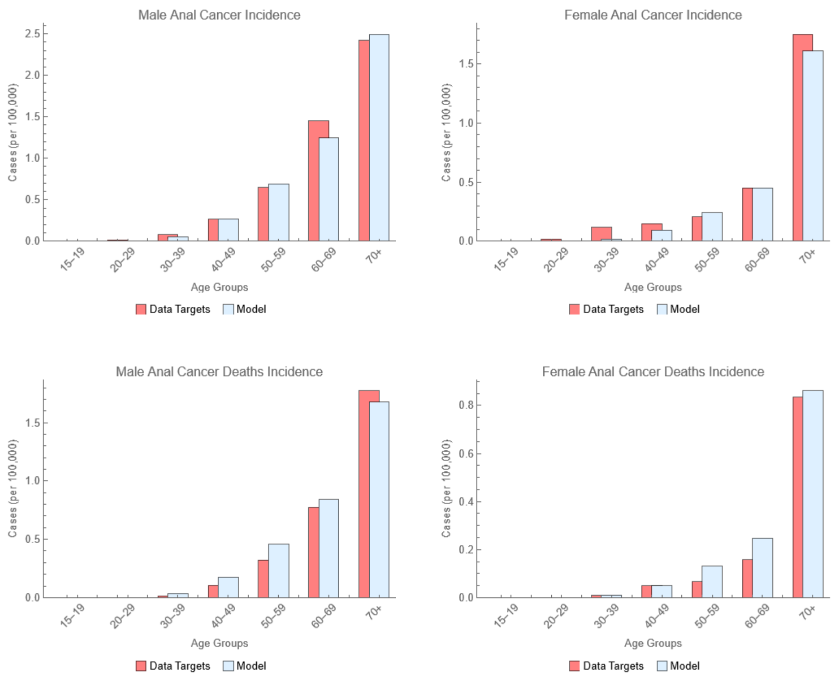


*Figure 17: Anal HPV 16 model fit to target data*

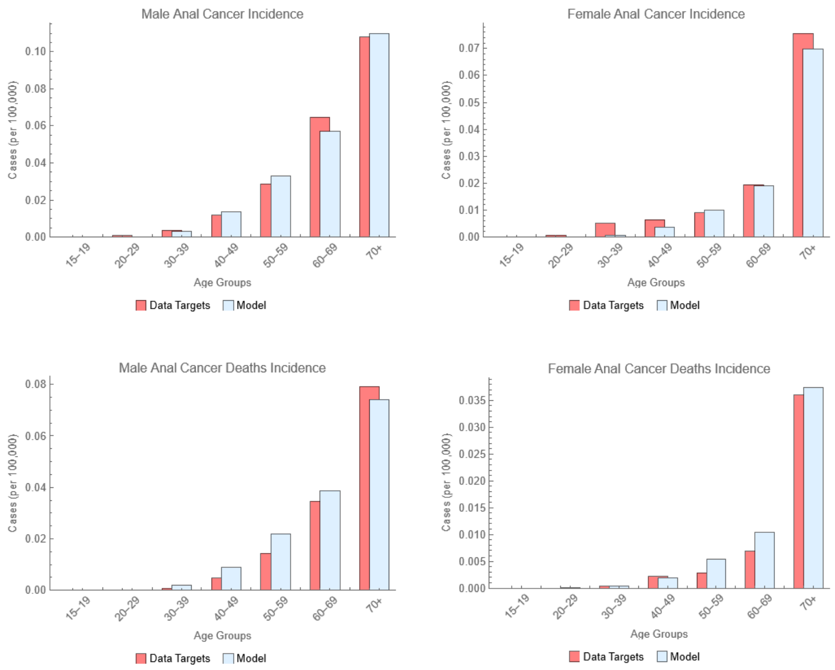


*Figure 18: Anal HPV 18 model fit to target data*

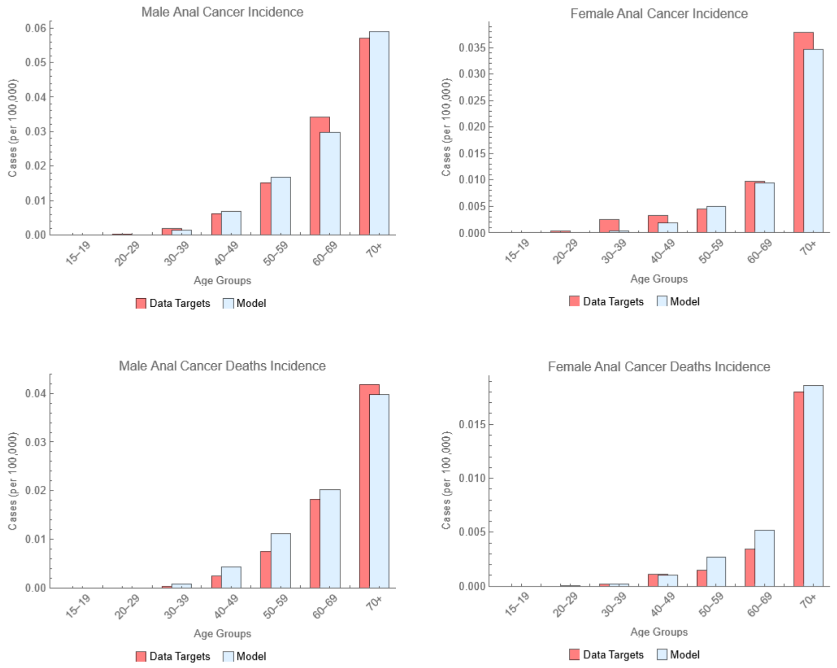


*Figure 19: Anal HPV 31 model fit to target data*

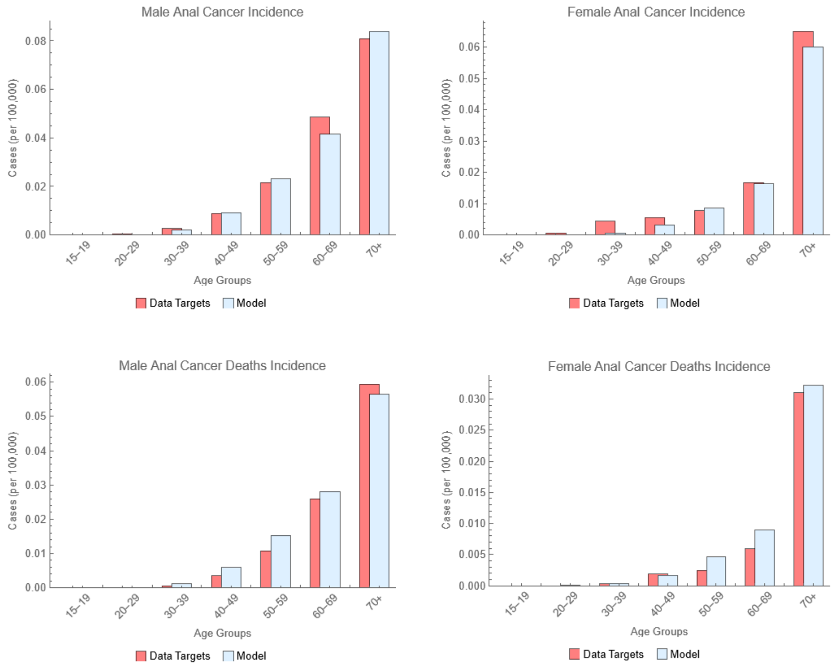


*Figure 20: Anal HPV 33 model fit to target data*

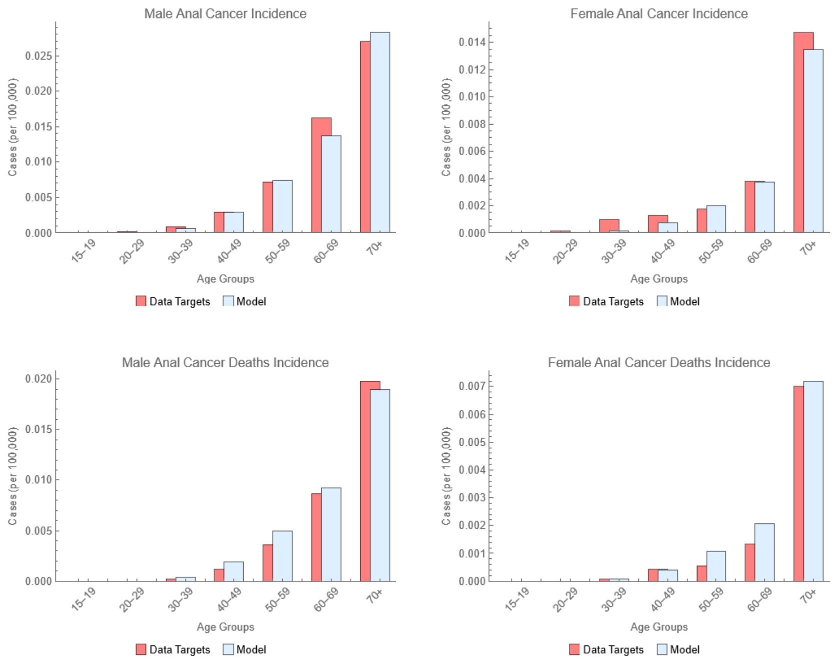


*Figure 21: Anal HPV 45 model fit to target data*

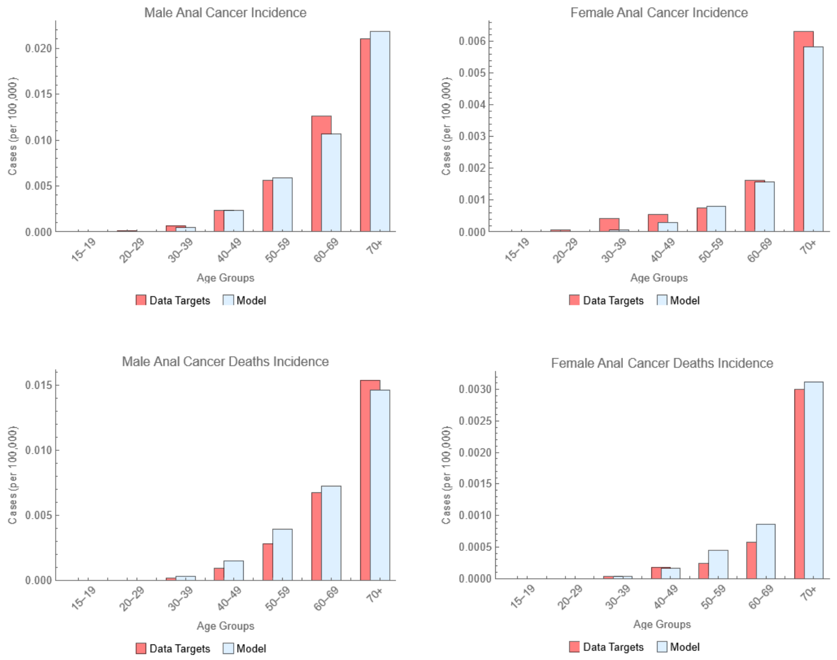


*Figure 22: Anal HPV 52 model fit to target data*

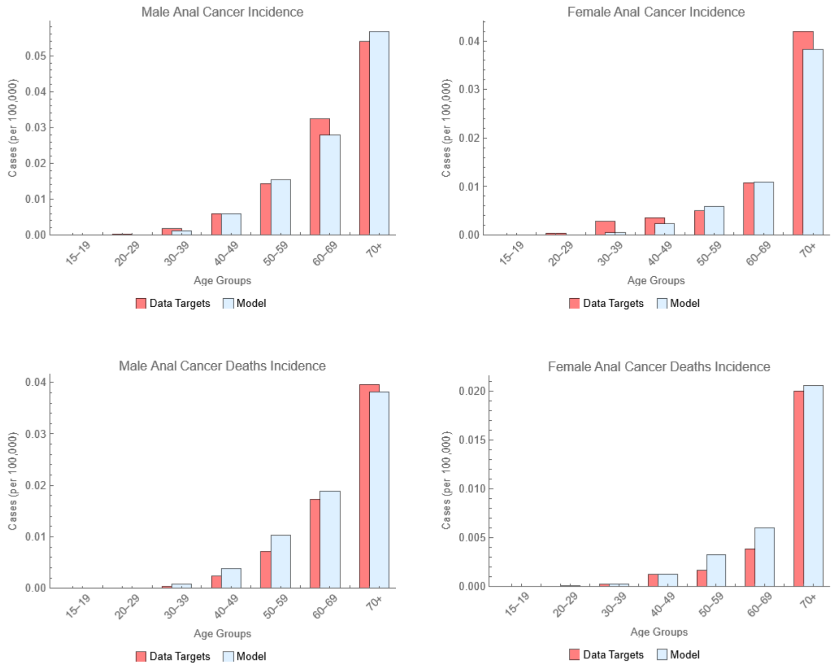


*Figure 23: Anal HPV 58 model fit to target data*

## E.6.3. Genital Warts Calibration

The residual function for the genital warts model calibration is

$$\sum_a \left(\frac{gwMale(a) - gwMale(a|\theta)}{Max(gwMale(a))}\right)^2 + \sum_a \left(\frac{gwFemale(a) - gwFemale(a|\theta)}{Max(gwFemale(a))}\right)^2$$

Figure 24 and Figure 25  show the resulting genital warts model fit to target data.

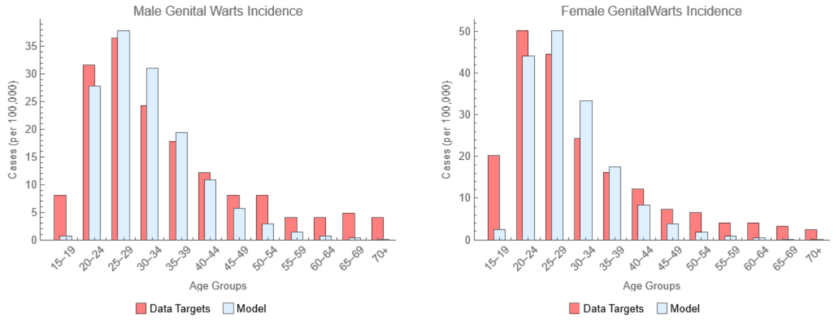


*Figure 24: Genital Warts HPV 6 model fit to target data*

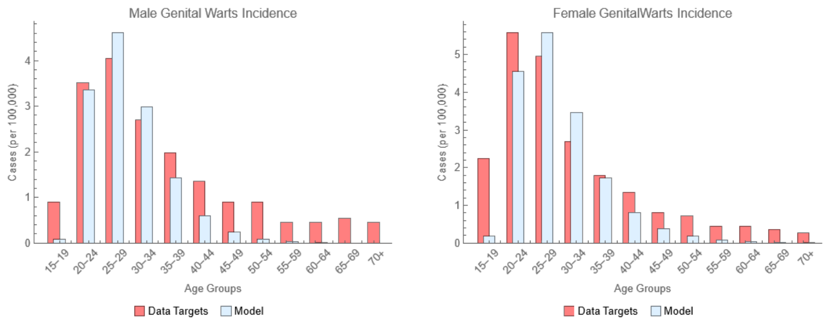


*Figure 25: Genital warts HPV 11 model fit to target data*

## E.6.4. Oropharyngeal Model Calibration

The oropharyngeal model residual function is

$$\sum_a \left(\frac{cancerMale(a) - cancerMale(a|\theta)}{Max(cancerMale(a))}\right)^2 + \sum_a \left(\frac{cancerFemale(a) - cancerFemale(a|\theta)}{Max(cancerFemale(a))}\right)^2 + \sum_a \left(\frac{cancerDeathsMale(a) - cancerDeathsMale(a|\theta)}{Max(cancerDeathsMale(a))}\right)^2 + \sum_a \left(\frac{cancerDeathsFemale(a) - cancerDeathsFemale(a|\theta)}{Max(cancerDeathsFemale(a))}\right)^2$$

For oropharyngeal cancer we only model HPV type 16, 18, 33, 45, and 58 since there is HPV 31 and 52 attributions for oropharyngeal cancer (see Table 40). Tables x-y show the resulting model fits to high-risk HPV types

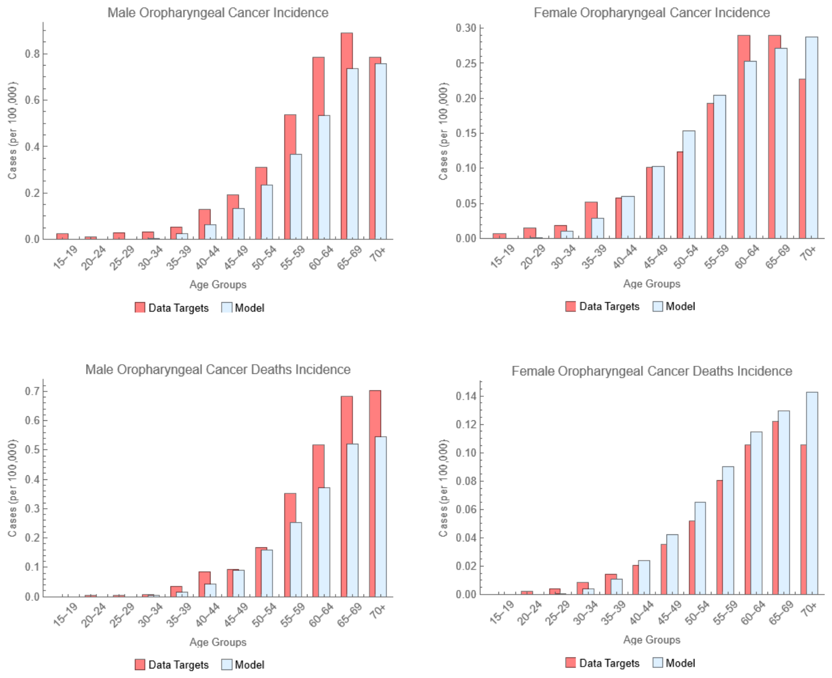


*Figure 26: Oropharyngeal HPV 16 model fit to target data*

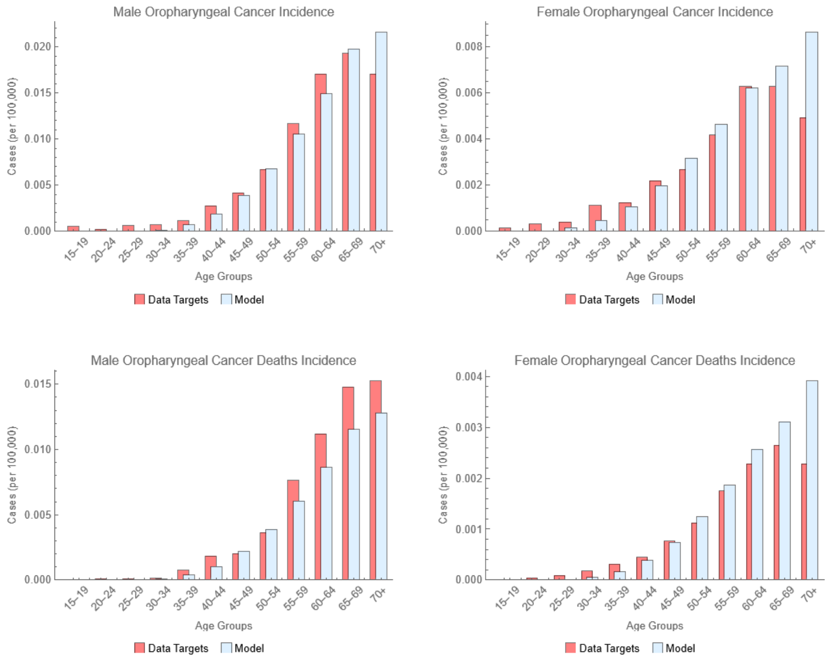


*Figure 27: Oropharyngeal HPV 18 model fit to target data*

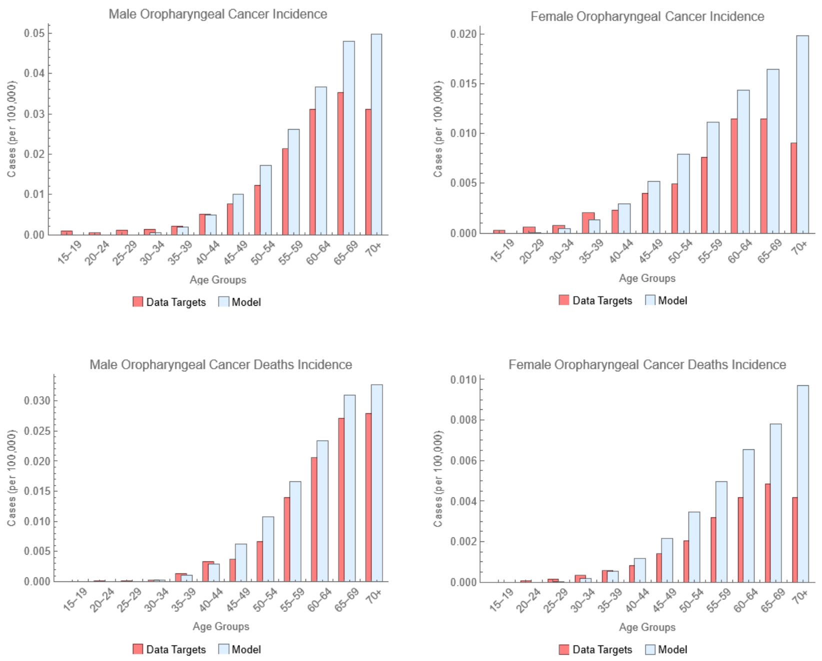


*Figure 28: Oropharyngeal HPV 33 model fit to target data*

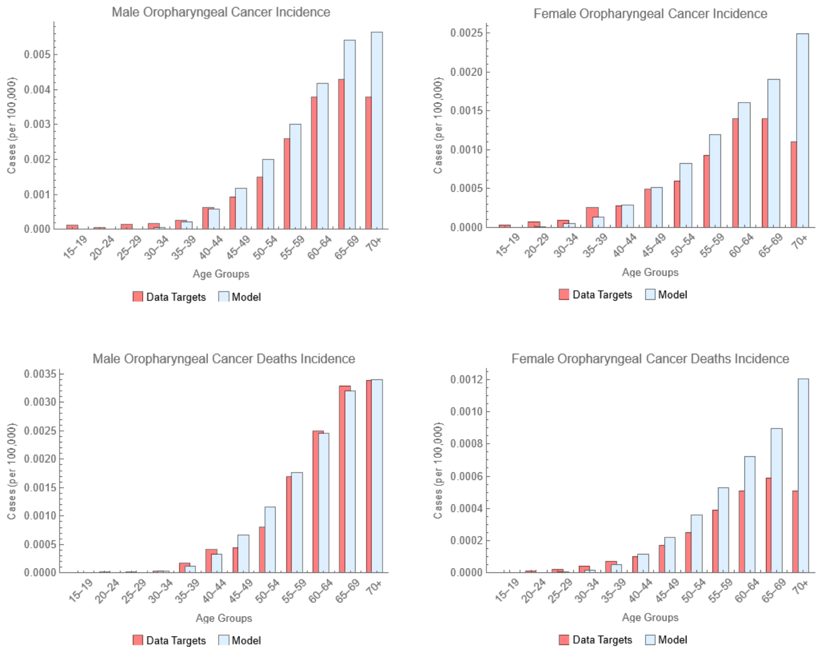


*Figure 29: Oropharyngeal HPV 45 model fit to target data*

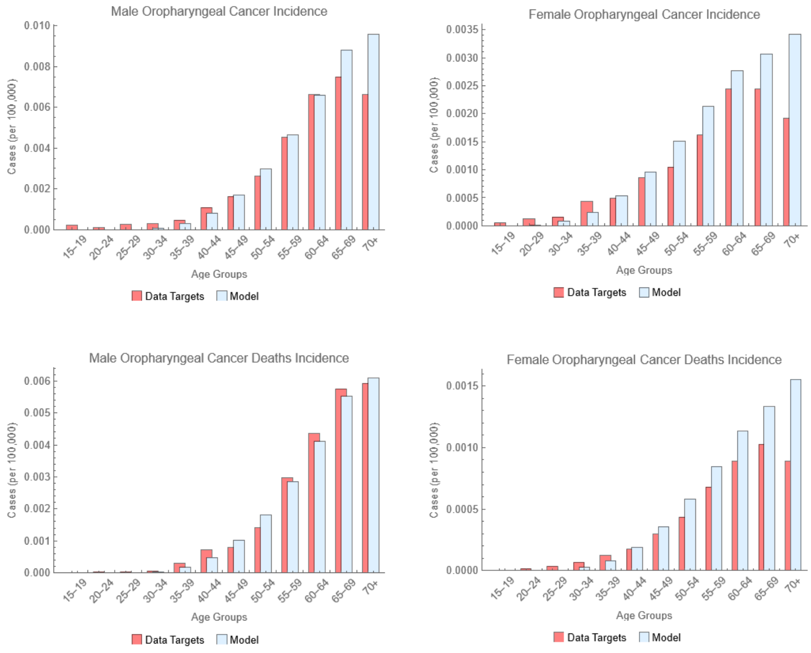


*Figure 30: Oropharyngeal HPV 58 model fit to target data*

## E.6.5. Penile Model Calibration

The penile model residual function is given by

$$\sum_{a}\left(\frac{cancer(a) - cancer(a|\theta)}{Max(cancer(a))}\right)^2 \sum_{a}\left(\frac{cancerDeaths(a) - cancerDeaths(a|\theta)}{Max(cancerDeaths(a))}\right)^2$$

The resulting model fits to target data are shown in Figures x-y.

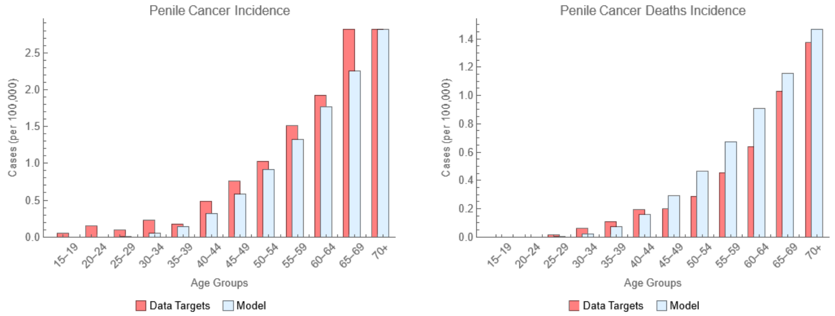


*Figure 31: Penile HPV 16 model fit to target data*

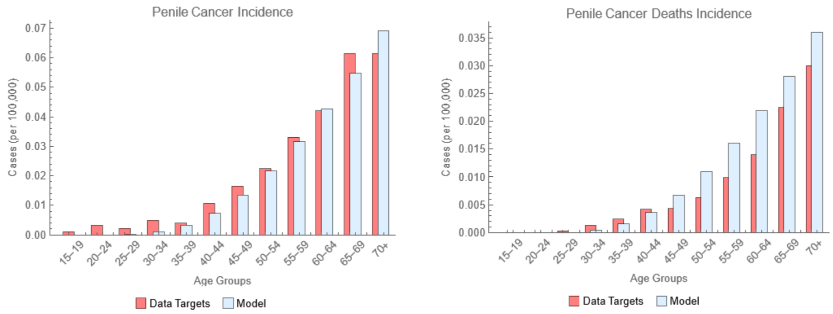


*Figure 32: Penile HPV 18 model fit to target data*

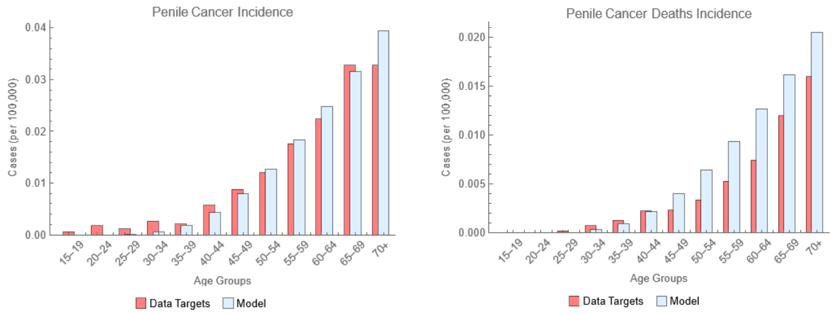


*Figure 33: Penile HPV 31 model fit to target data*

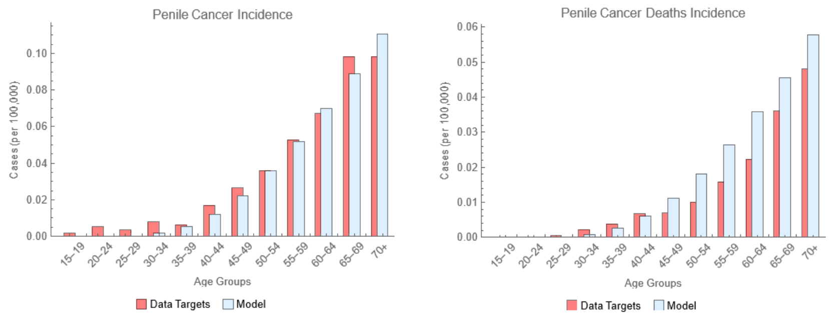


*Figure 34: Penile HPV 33 model fit to target data*

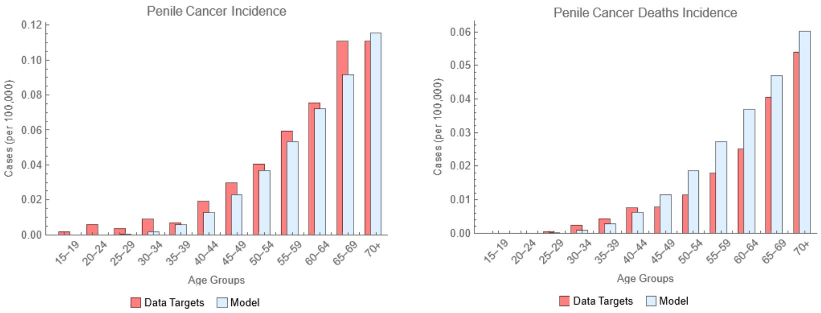


*Figure 35: Penile HPV 45 model fit to target data*

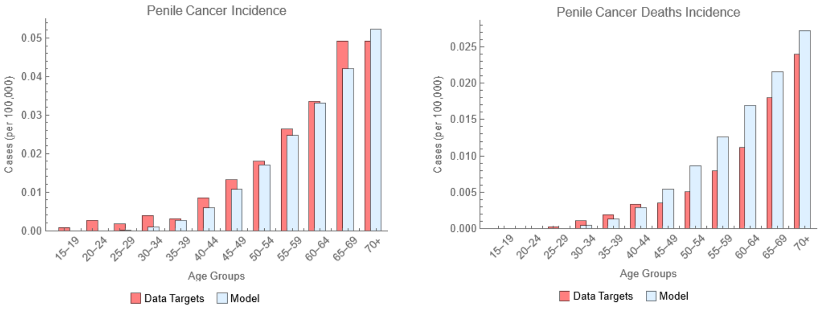


*Figure 36: Penile HPV 52 model fit to target data*

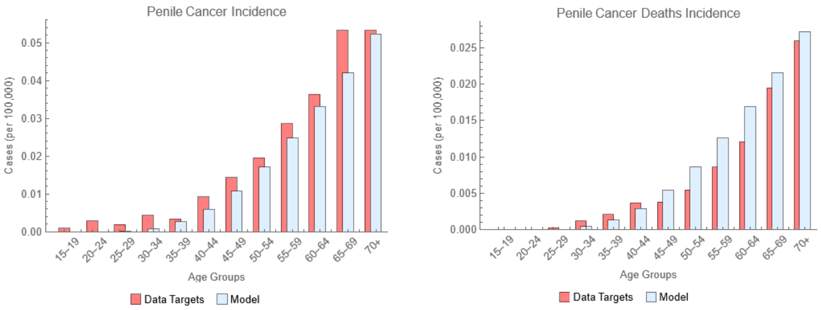


*Figure 37: Penile HPV 58 model fit to target data*

## E.6.6. Vaginal Model Calibration

The vaginal model residual function is given by

$$\sum_a \left(\frac{cancer(a) - cancer(a|\theta)}{Max(cancer(a))}\right)^2 \sum_a \left(\frac{cancerDeaths(a) - cancerDeaths(a|\theta)}{Max(cancerDeaths(a))}\right)^2$$

The resulting model fits to target data are shown in Figures x-y.

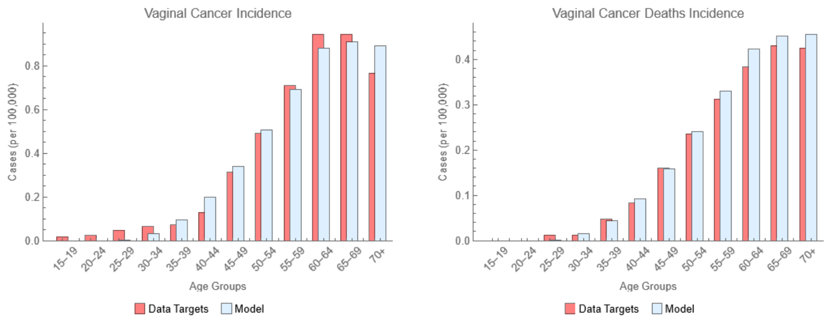


*Figure 38: Vaginal HPV 16 model fit to target data*

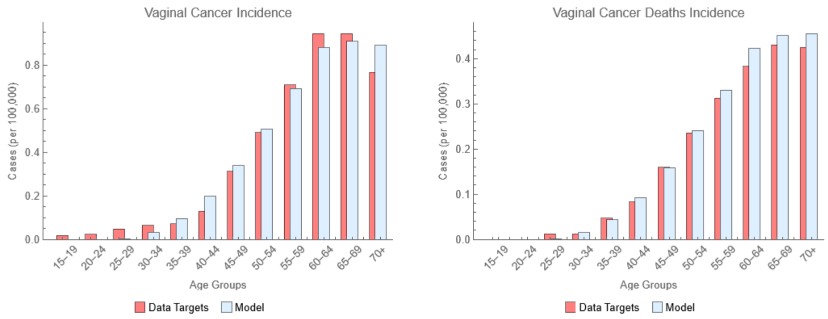


*Figure 39: Vaginal HPV 18 model fit to target data*

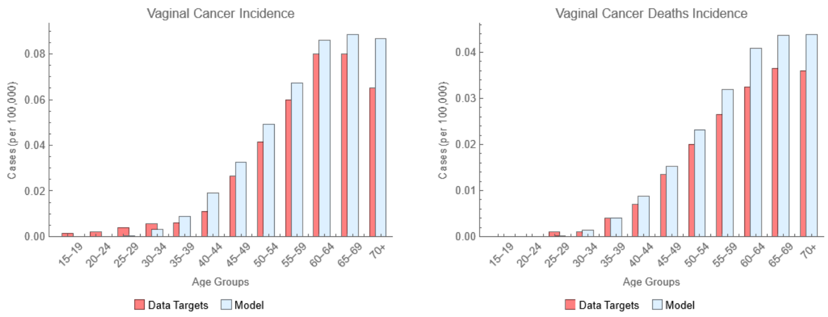


*Figure 40: Vaginal HPV 31 model fit to target data*

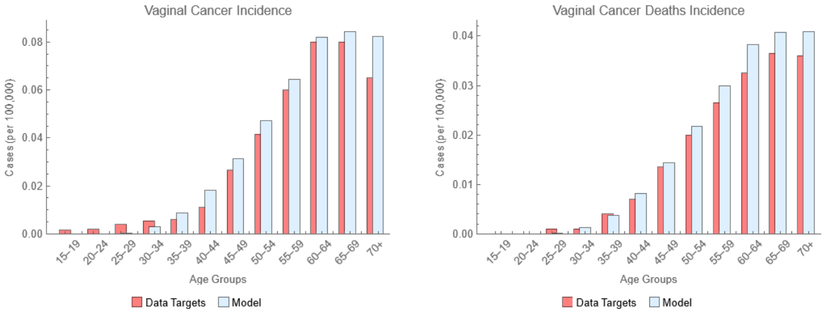


*Figure 41: Vaginal HPV 33 model fit to target data*

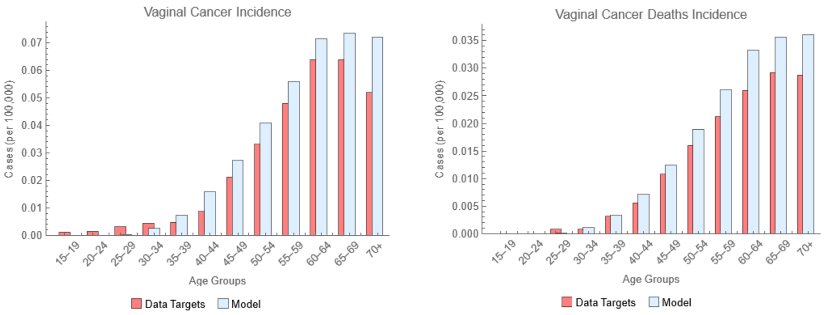


*Figure 42: Vaginal HPV 45 model fit to target data*

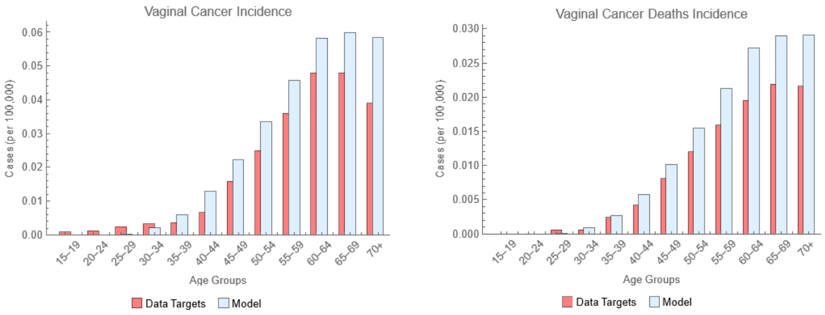


*Figure 43: Vaginal HPV 52 model fit to target data*

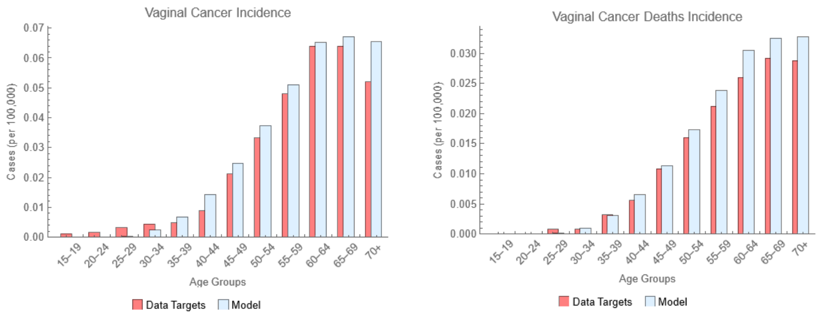


*Figure 44: Vaginal HPV 58 model fit to target data*

## E.6.7. Vulvar Model Calibration

The vulvar model residual function is given by

$$\sum_{a}\left(\frac{cancer(a)-cancer(a|\theta)}{Max(cancer(a))}\right)^{2}\sum_{a}\left(\frac{cancerDeaths(a)-cancerDeaths(a|\theta)}{Max(cancerDeaths(a))}\right)^{2}$$

The resulting model fits to target data are shown in Figures x-y.

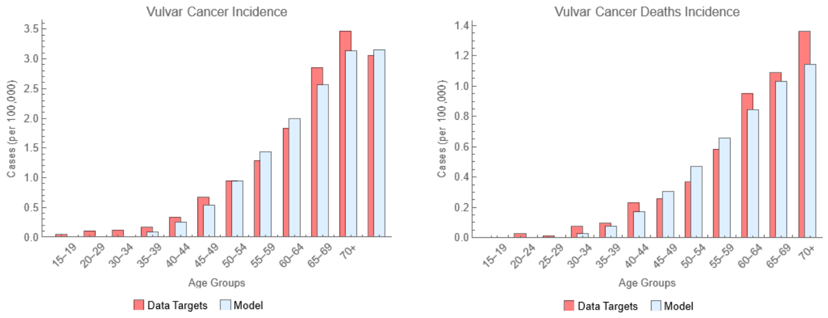


*Figure 45: Vulvar HPV 16 model fit to target data*

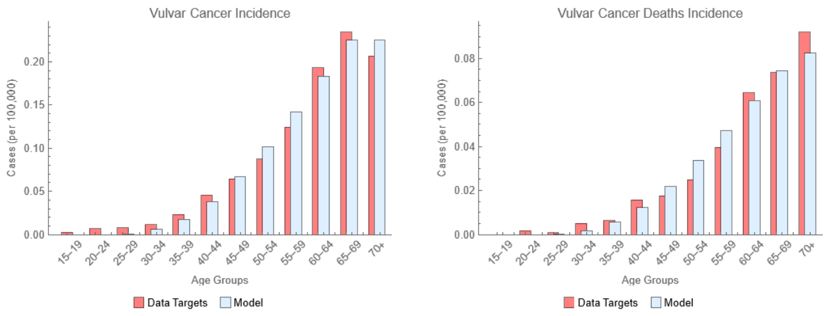


*Figure 46: Vulvar HPV 18 model fit to target data*

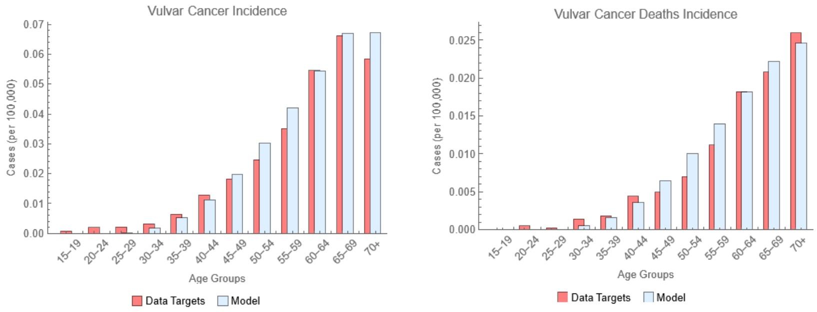


*Figure 47: Vulvar HPV 31 model fit to target data*

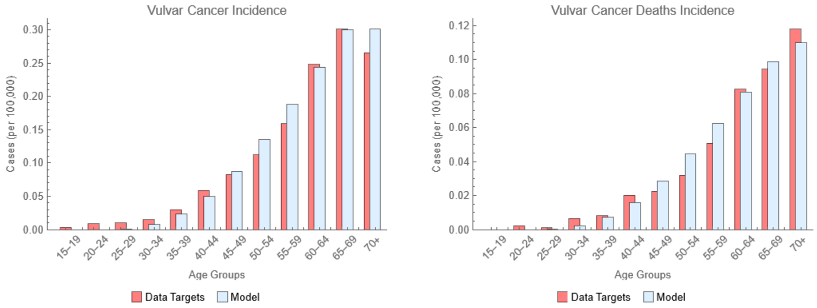


*Figure 48: Vulvar HPV 33 model fit to target data*

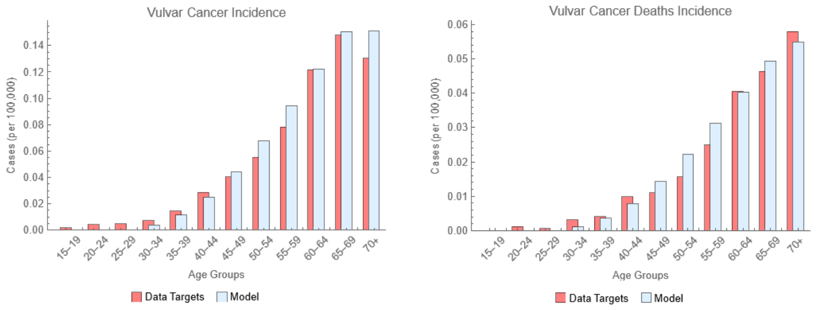


*Figure 49: Vulvar HPV 25 model fit to target data*

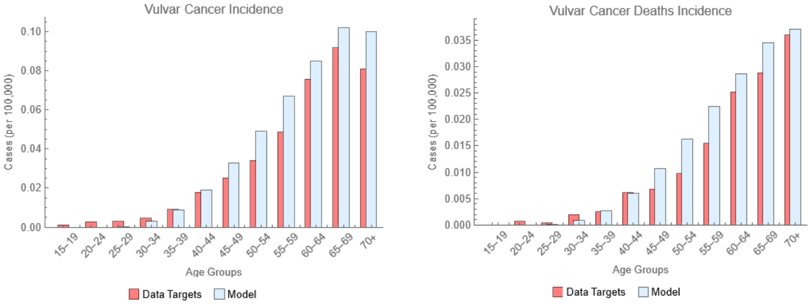


*Figure 50: Vulvar HPV 52 model fit to target data*

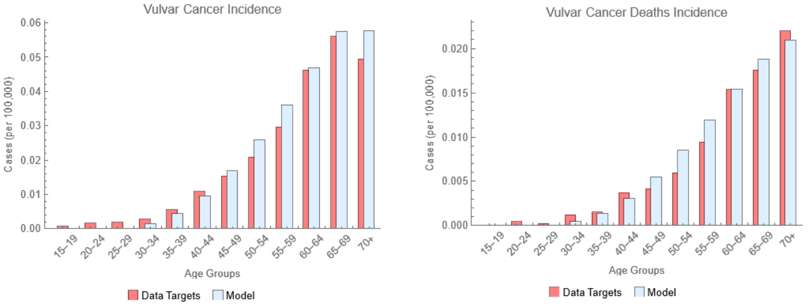


*Figure 51: Vulvar HPV 58 model fit to target data*

# Appendix F: Cost and Health Utility Inputs

## F.1. Treatment Costs

Treatment costs in the model are assumed to include all costs associated with an episode of the disease or cancer. These costs include diagnostic as well as treatment costs.

Cervical CIN and Cancer treatment costs are from Kosen[35] and shown in Table 51. Which was an Indonesia CE modeling study. All costs are based on local expert opinion and represent episode of care. Costs were inflated to USD 2021 using CPIs from Malaysian Ministry of Finance and the Central Bank of Malaysia.

*Table 51: Cervical disease and cancer related treatment costs*

| | | Cervical |
|---|---|---|
| Disease | CIN 1 | $95.50 |
| | CIN 2 | $193.00 |
| | CIN 3 | $868.20 |
| Cancer | Local | $4,862.10 |
| | Regional | $9,608.40 |

| | |
|---|---|
| Distant | $3,472.90 |

Vaginal, vulvar, penile, anal, and head and neck cancer costs are from Choi[36] and are shown in Table 52. Costs are from 2012 in South Korea and for any cancer (not broken down by stage) and represent cost per patient. The values were converted to MYR 2012 costs, inflated to MYR 2021 and converted to USD 2021 based on CPIs and exchange rates published the Malaysian Ministry of Finance and the Central Bank of Malaysia.

*Table 52: Treatment costs for all non-cervical cancers*

| **Cancer** | | **Local** | **Regional** | **Distant** |
|---|---|---|---|---|
| Vaginal | Female | $19,431.00 | $19,431.00 | $19,431.00 |
| Vulvar | Female | $13,510.00 | $13,510.00 | $13,510.00 |
| Penile | Male | $10,528.00 | $10,528.00 | $10,528.00 |
| Anal | Female | $17,093.00 | $17,093.00 | $17,093.00 |
| | Male | $16,077.00 | $16,077.00 | $16,077.00 |
| Oropharyngeal | Female | $17,536.00 | $17,536.00 | $17,536.00 |
| | Male | $24,024.00 | $24,024.00 | $24,024.00 |

Cost sensitivity was done as a multi-way sensitivity varying all treatment costs from -20% to +20%.

## F.2. Vaccine Price

Vaccine prices were derived from Setiawan et. al[37] CE analysis for Indonesia. Given the 4vHPV GAVI price of $4.60 per dose, we choose our base case 9vHPV to be approximately 3 times as expensive ($14), and assumed $6 for administration and other dose related costs, leading to a 9vHPV price of $20 per dose, including dose administration and all other related costs. For the low-price sensitivity analyses, we assume combined dose price and administration costs of $10. For the high-price sensitivity we assume a combined dose price and administration of $60.

## F.3. Health State Utilities

### F.3.1. Baseline utilities

Baseline utilities were provided from Szende[38] (https://pubmed.ncbi.nlm.nih.gov/29787044/). Self-reported EQ5-D utilities for Thailand were used. Data were for 18+. Assume that <18 is the same as 18 year-old utilities. Values are shown in Table 53.

*Table 53: General population average health state by age group and sex*

| **Age Groups** | **Male** | **Female** |
|---|---|---|

| | | |
|---|---|---|
| 0-17 | 0.855 | 0.785 |
| 18-24 | 0.855 | 0.785 |
| 25-34 | 0.786 | 0.784 |
| 35-44 | 0.803 | 0.75 |
| 45-54 | 0.717 | 0.717 |
| 55-64 | 0.733 | 0.66 |
| 65-74 | 0.669 | 0.67 |
| 75+ | 0.609 | 0.726 |

## F.3.2. Cervical disease

Cervical related health utilities are in Table 54. Cervical CIN utilities are taken from Termrungruanglert, et al.[39] The variation is obtained by assuming ±20% but not exceeding 1.0. Cervical cancer utilities are derived from Endarti et al.[40] where we mapped stage I to local cancer, stage II to regional cancer. The variations are the 95% CI. For distant cancer we use the stage II value since the study shows higher utility for stage IV than stage II, likely due to the very small sample size. In addition, we assumed that the survivor utility was equivalent to local cancer utility.

*Table 54: Health utilities for cervical disease and cancer*

| | | Utility | variation | Notes |
|---|---|---|---|---|
| Disease | CIN 1 | 0.91 | (0.728, 1) | Termrungruanglert[39] |
| | CIN 2 | 0.87 | (0.696, 1) | Termrungruanglert[39] |
| | CIN 3 | 0.87 | (0.696, 1) | Termrungruanglert[39] |
| | CIS | 0.87 | (0.696, 1) | Termrungruanglert[39] |
| Cancer | Local | 0.85 | (0.73, 0.97) | Endarti[40] |
| | Regional | 0.76 | (0.70, .83) | Endarti[40] |
| | Distant | 0.76 | (0.70, .83) | Assumed |
| | Survivor | 0.85 | (0.73, 0.97) | Assumed |

## F.3.3. Vaginal, Vulvar, Penile

Utilities for vaginal, vulvar, penile are in Table 55 and are from the mean values in Conway, et al.[41] The variation is the 95% CI. Utilities were not stage specific, so we assume the same value for all stages in the model. The cancer survivor utility is assumed to be 0.76 except for penile where the cancer utilities were higher than .76 and we assumed the same utility as for cancer with a minimum of -20% and maximum of 1.0. In all other cases the variation is ±20%.

*Table 55: Vaginal, Vulvar, and Penile cancer utilities*

| | Vaginal | Vulvar | Penile |
|---|---|---|---|

| | | | | |
|---|---|---|---|---|
| Cancer | Local | 0.59 (.53, .63) | 0.65 (.6, .7) | 0.79 (.74, .84) |
| | Regional | 0.59 (.53, .63) | 0.65 (.6, .7) | 0.79 (.74, .84) |
| | Distant | 0.59 (.53, .63) | 0.65 (.6, .7) | 0.79 (.74, .84) |
| | Survivor | 0.76 (0.61, .91) | 0.76 (0.61, .91) | 0.79 (.74, 1.0) |

### F.3.4. Anal

Anal cancer utilities are in Table 56 and were the mean values from Conway, et al.[41] The variation is the 95% CI. Utilities were not stage or sex specific, so we assume the same value for all stages and both sexes in the model. The cancer survivor utility is assumed to be 0.76. Variation is ±20%

*Table 56: Anal cancer utilities.*

| | | Male | Female |
|---|---|---|---|
| Cancer | Local | 0.570 (0.52, 0.62) | 0.570 (0.52, 0.62) |
| | Regional | 0.570 (0.52, 0.62) | 0.570 (0.52, 0.62) |
| | Distant | 0.570 (0.52, 0.62) | 0.570 (0.52, 0.62) |
| | Survivor | 0.76 (0.61, .91) | 0.76 (0.61, .91) |

### F.3.5. Oropharyngeal

Head and neck cancer utilities are in Table 57 and were from Conway, et al.[41] oropharyngeal cancer utilities. Utilities were not stage or sex specific, so we assume the same value for all stages and both sexes in the model. The cancer survivor utility is assumed to be 0.76.

*Table 57: Oropharyngeal cancer utilities*

| | | Male | Female |
|---|---|---|---|
| Cancer | Local | 0.580 (0.53, 0.63) | 0.580 (0.53, 0.63) |
| | Regional | 0.580 (0.53, 0.63) | 0.580 (0.53, 0.63) |
| | Distant | 0.580 (0.53, 0.63) | 0.580 (0.53, 0.63) |
| | Survivor | 0.76 (0.61, .91) | 0.76 (0.61, .91) |

### F.3.6. Genital Warts

Genital warts health state utilities are 0.91 for both males and females from Meyers et al.[42]

# Appendix G: Additional Results

## G.1. Cases Avoided

Figure 52 shows the non-cervical cancer cases avoided (as compared to no vaccination), by cancer type, and by vaccination strategy, over a 100-year time horizon, separated by coverage scenario. Panel A and panel B shows results for the base case and alternative distributions, respectively. Vulvar cancer accounts for the largest portion of non-cervical cancers followed by penile, male anal, vaginal, female anal, male H&N, and finally female H&N. For both distributions, penile, male anal, and vaginal account for similar proportions of the non-cervical cancers, while female anal, female and male H&N account for the smallest proportions.

In the alternative distribution, the GNV 1-dose program can have, on average,  better impact than the girls only 2-dose program in terms of avoiding non-cervical cancers but the has a lower lower-bound.

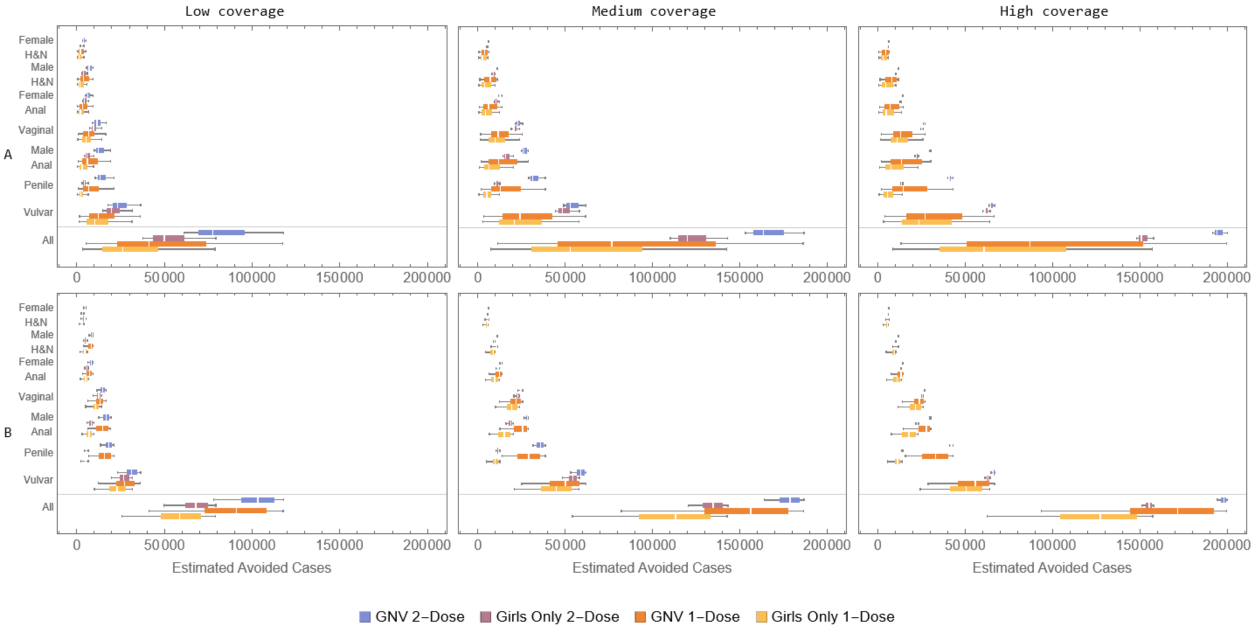


*Figure 52: Estimated non-cervical cancer cases avoided compared to no vaccine by coverage scenario, and by cancer type for the four different vaccination strategies, over a 100-year horizon for the (A) base case distribution and (B) alternative distributions. The boxes in the boxplots cover the 25% to 75% quartiles, the white vertical lines represent the median and whiskers represent minimum and maximum values in the distribution.*

## G.2. Cost Effectiveness Sensitivity

### G.2.1. Base Case Distribution

The one-way sensitivity of cost-effectiveness to dose-price, discount rate, treatment costs, and health state utilities is presented in Figure 53 through Figure 60  showing cost-effectiveness acceptability curves and cost-effectiveness frontier plots for expected values of costs and QALYs, and

Table 58 through Table 61 shows the cost-effectiveness of expected values tables.

Figure 54 shows the cost-effectiveness acceptability for the base-case distribution assuming a lower, base case, and higher per dose cost (including administration costs) of $10 USD, $20 USD, and $60 USD, respectively, and the three coverage scenarios. For the high dose price, with low coverage, the 2-dose GNV has nearly 100% probability of being cost-effective at 1 GDP WTP threshold, whereas in the medium or high coverage scenarios, the girls-only 2-dose has nearly 100% probability of being cost-effective at the 1 GDP WTP threshold. The no vaccine scenario has the highest probability for being cost-effective at very low WTP thresholds (less than about $100/QALY) for all high dose cost coverages. For the low dose cost, the 2-dose GNV program has nearly 100% probability of being cost-effective from WTP of about $200/QALY, $500 QALY, and $1000/QALY for low, medium, and high coverage scenarios, respectively; otherwise, the 2-dose girls-only scenarios are probably cost-effective. The 1-dose strategies have very low probability of being cost-effective in any of the coverage or dose cost variations. As either dose price or coverage increases, the range of WTP values for which girls-only 2-dose programs have a high probability of being cost-effective increases. For example, with low coverage and low dose price, girls-only 2-dose is most likely cost-effective for WTP less than about $100/QALY, while for high coverage and high dose price that range is from about $100/QALY to about $7000/QALY (nearly 2xGDP). Generally, as dose cost increases the less effective strategies have increased probability of being cost-effective.

Table 58 shows the ICERs of expected values of costs and QALYs for variations in per-dose cost. The 1-dose strategies are dominated in all the dose cost and coverage scenarios considered. The girls-only 2-dose strategies are cost saving (compared to no vaccine) except for the $60 per dose price scenario where this strategy has a $140/QALY to $186/QALY ICER corresponding to the low and high coverage scenarios, and the no vaccine scenario has the lowest cost. The GNV 2-dose strategy ICERs increase with dose cost from $169/QALY to $2,147/QALY, $512/QALY to $4,337/QALY and $1,081/QALY to $7,732/QALY, for the low, medium, and high coverage scenarios, respectively.

### G.2.1.1. Dose price variation

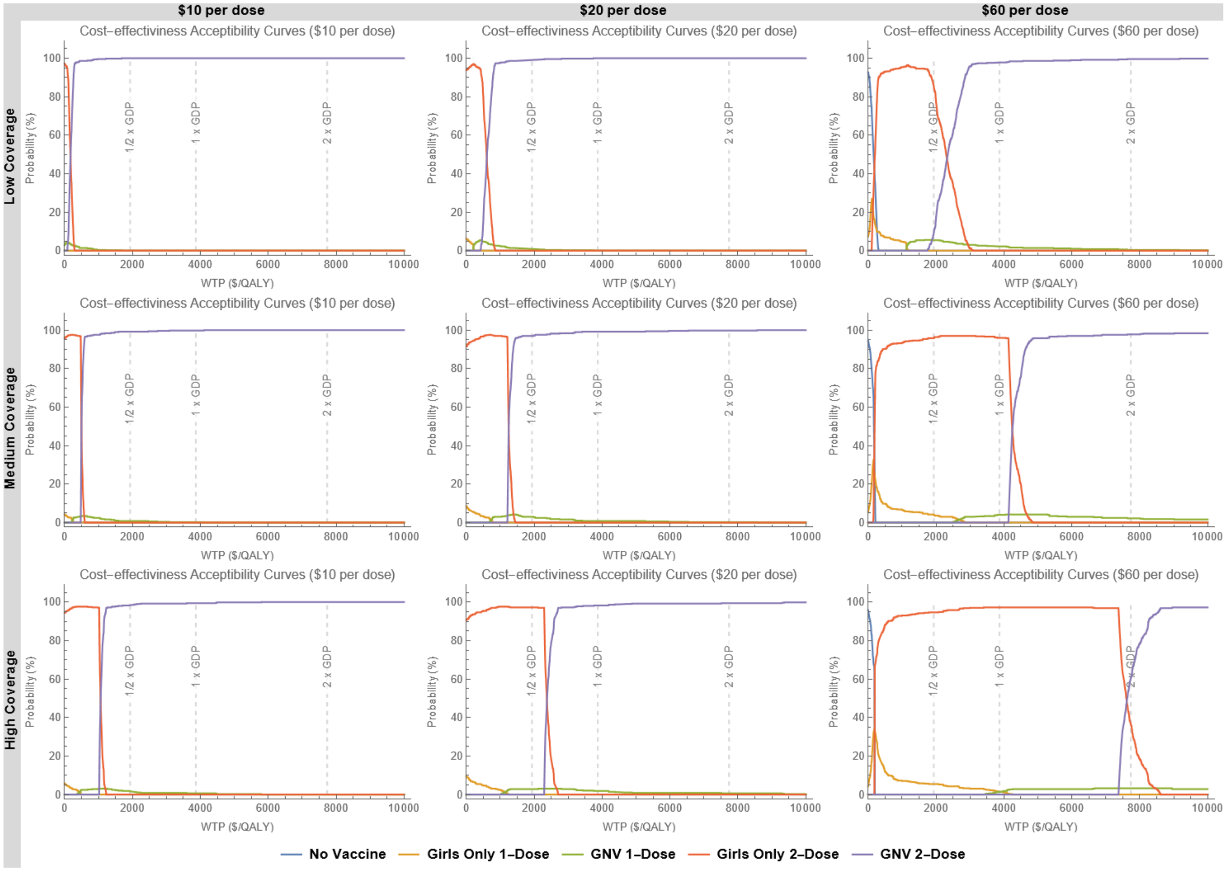


*Figure 53: Dose cost sensitivity of cost-effectiveness acceptability for each coverage scenario*

*Table 58: Dose price sensitivity of cost-effectiveness frontier for expected values of costs and QALYs for each coverage scenario. ICERs are rounded to nearest $.*

| | Strategy | $10 per Dose | | | $20 per Dose (base-case price) | | | $60 per Dose | | |
|---|---|---|---|---|---|---|---|---|---|---|
| | | Cost | QALYs | ICER (ΔC/ΔQ) | Cost | QALYs | ICER (ΔC/ΔQ) | Cost | QALYs | ICER (ΔC/ΔQ) |
| Low Coverage | No Vaccine | 57.53829 | 23.93433 | Dominated | 57.53829 | 23.93433 | Dominated | 57.53829 | 23.92296 | - |
| | Girls only 1-Dose | 55.79894 | 23.94532 | Dominated | 56.65939 | 23.94532 | Dominated | 60.10120 | 23.93446 | Dominated |
| | GNV 1-Dose | 56.23700 | 23.94728 | Dominated | 58.01421 | 23.94728 | Dominated | 65.12305 | 23.93653 | Dominated |
| | Girls only 2-Dose | 54.19803 | 23.95287 | - | 55.41127 | 23.95287 | - | 60.26422 | 23.94238 | 140 |
| | GNV 2-Dose | 54.71949 | 23.95595 | 169 | 57.22536 | 23.95595 | 589 | 67.24883 | 23.94564 | 2,147 |
| Medium Coverage | No Vaccine | 57.53829 | 23.93433 | Dominated | 57.53829 | 23.93433 | Dominated | 57.53829 | 23.93433 | - |
| | Girls only 1-Dose | 54.10187 | 23.95689 | Dominated | 55.98209 | 23.95689 | Dominated | 63.50298 | 23.95689 | Dominated |
| | GNV 1-Dose | 55.36125 | 23.95991 | Dominated | 59.24473 | 23.95991 | Dominated | 74.77865 | 23.95991 | Dominated |
| | Girls only 2-Dose | 49.40069 | 23.98017 | - | 52.54066 | 23.98017 | - | 65.10054 | 23.98017 | 165 |

| | | | | | | | | | | |
|---|---|---|---|---|---|---|---|---|---|---|
| | GNV 2-Dose | 51.64145 | 23.98455 | 512 | 58.12686 | 23.98455 | 1,277 | 84.06851 | 23.98455 | 4,337 |
| High Coverage | No Vaccine | 57.53829 | 23.93433 | Dominated | 57.53829 | 23.93433 | Dominated | 57.53829 | 23.93433 | - |
| | Girls only 1-Dose | 53.64443 | 23.96029 | Dominated | 55.85785 | 23.96029 | Dominated | 64.71152 | 23.96029 | Dominated |
| | GNV 1-Dose | 55.22672 | 23.96344 | Dominated | 59.79840 | 23.96344 | Dominated | 78.08511 | 23.96344 | Dominated |
| | Girls only 2-Dose | 47.44599 | 23.9926 | - | 51.62934 | 23.99260 | - | 68.36278 | 23.99260 | 186 |
| | GNV 2-Dose | 51.06664 | 23.99595 | 1,081 | 59.70711 | 23.99595 | 2,411 | 94.26899 | 23.99595 | 7,732 |

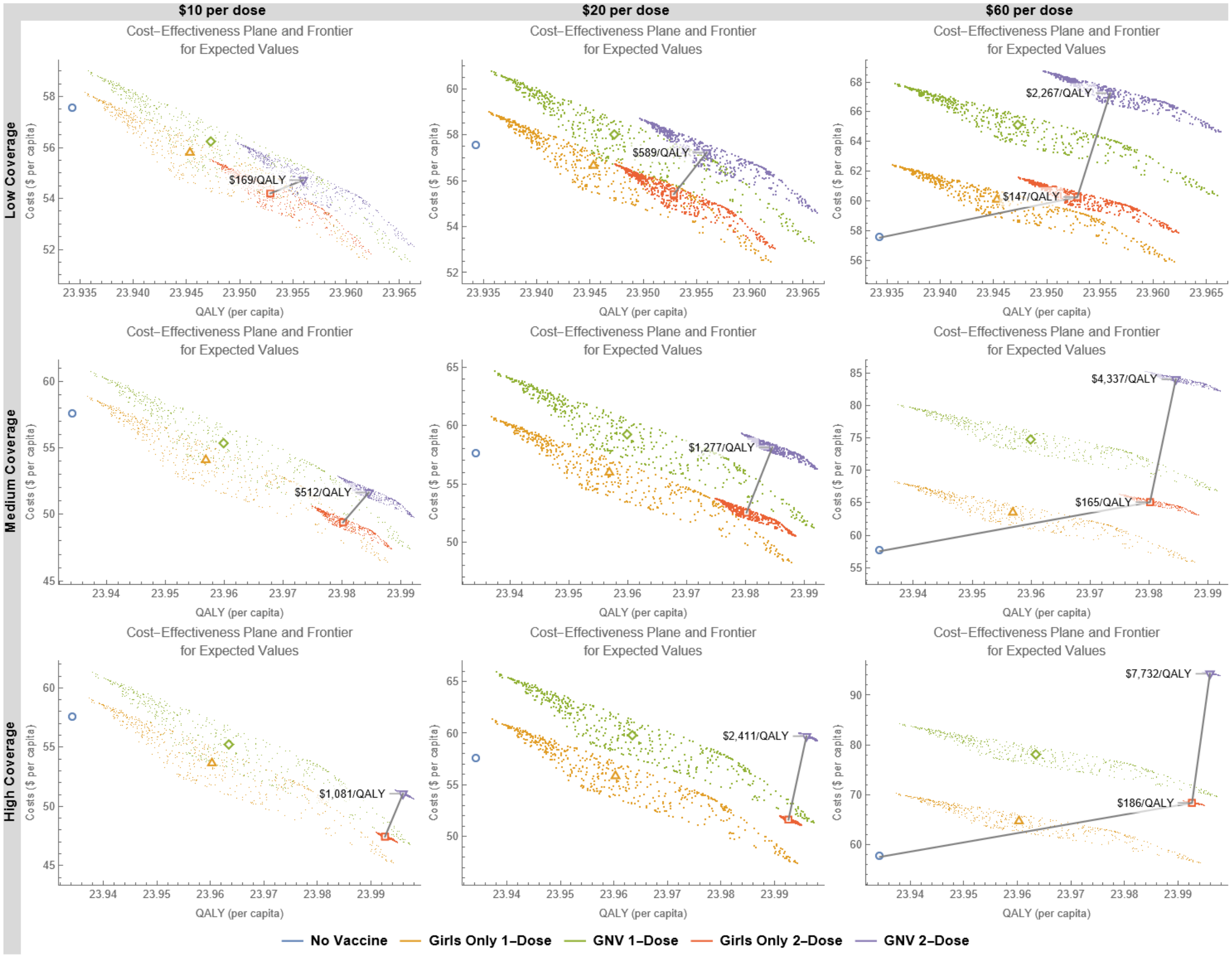


*Figure 54: Dose price sensitivity of cost-effectiveness plane showing expected value frontier*

### G.2.1.2. Discount variations

Figure 56 shows the cost-effectiveness acceptability curves for no discounting, 3% discounting (base case), and 5% discounting of QALYs and costs. For the no discounting case, for all coverage scenarios, both 1-dose programs had very low probability (<5%) of being cost-effective for WTP less than about

$1,200/QALY. The GNV 2-dose program is 100% likely to be cost-effective at 0.5 GDP threshold and above, and the girls-only 2-dose is 95% to 100% cost-effective at low WTP thresholds (~$0/QALY to ~$750/QALY). In the 5% discount rate scenario with low coverage, the probability of being cost-effective at the 0.5xGDP threshold was approximately 70% for GNV 2-dose, 25% for girls-only 2-dose, and 5% for GNV 1-dose. In the 5% discount with medium coverage, the probability of being cost-effective at the 1xGDP threshold was approximately 95% for GNV 2-dose and 5% for GNV 1-dose. The other vaccination strategies have 0% probability of being cost-effective above 1xGDP. For the 5% discount with high coverage, the probability of being cost-effective at 1xGDP was about 95% for girls-only 2-dose and 5% for GNV 1-dose. As with the dose-cost sensitivity, as discounting and coverage increase, the WTP range for which girls-only 2-dose is most likely cost-effective increases and, correspondingly, the WTP at which GNV 2-dose becomes most cost-effective increases.

The ICERs of expected values by discount and coverage are shown in Table 59. For all scenarios, the 1-dose strategies are dominated. For the 0% discounting, girls-only 2-dose is cost saving for all coverages. For 5% discounting, girls-only 2-dose remains cost saving for low coverage and had an ICER (relative to "no vaccine") of $9/QALY for medium coverage and $25/QALY for high coverage. The GNV 2-dose ICERs increase with discount from $80/QALY to $1,685/QALY, $330/QALY to $3,197/QALY, and $803/QALY to $5,437/QALY, for low, medium, and high coverage scenarios, respectively.

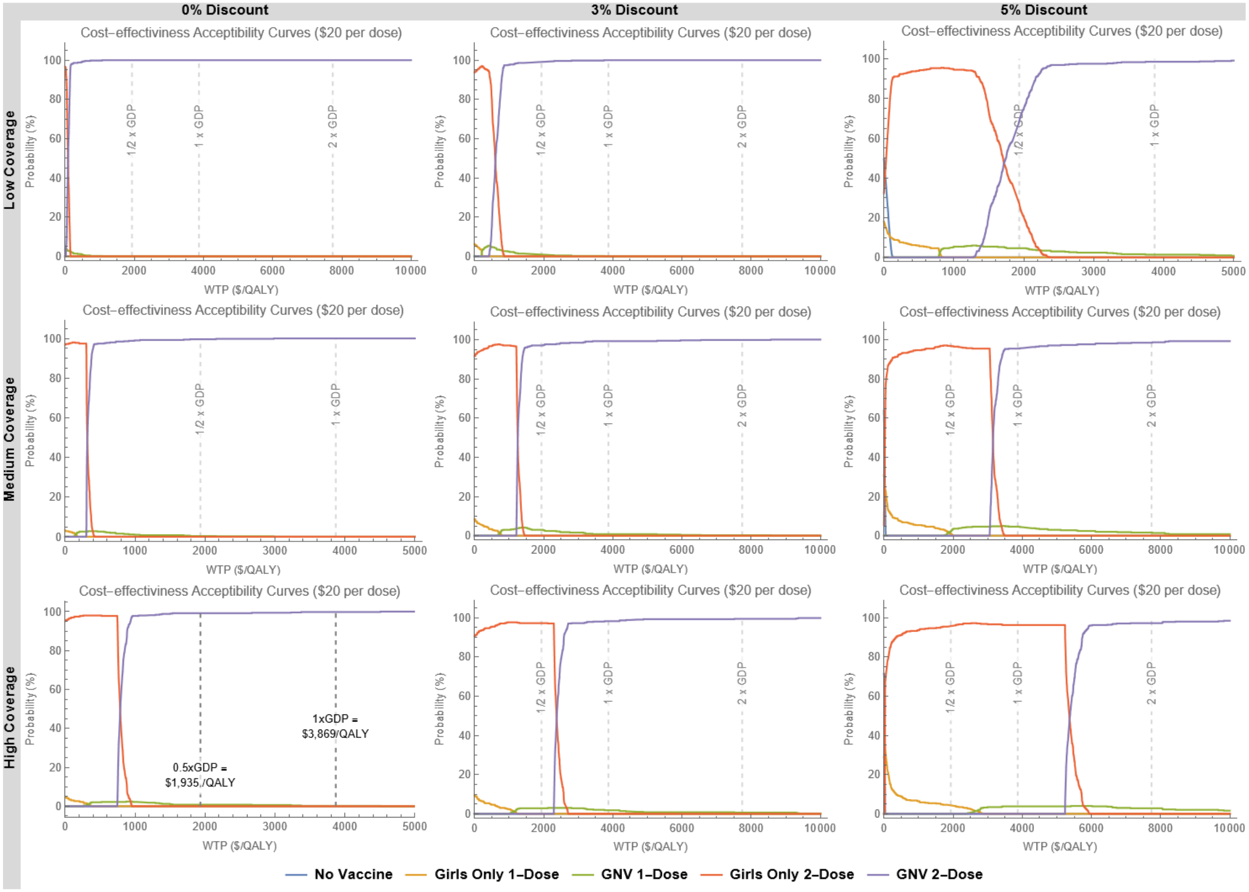


*Figure 55: Cost-effectiveness acceptability sensitivity to discount rate*

*Table 59: Discount sensitivity of cost-effectiveness frontier for expected values of costs and QALYs for each coverage scenario. ICERs are rounded to nearest $.*

| | Strategy | 0% Discounting | | | 3% Discounting (Base-case) | | | 5% Discounting | | |
|---|---|---|---|---|---|---|---|---|---|---|
| | | Cost (USD) | QALYs | ICER (ΔC/ΔQ) | Cost (USD) | QALYs | ICER (ΔC/ΔQ) | Cost (USD) | QALYs | ICER (ΔC/ΔQ) |
| Low Coverage | No Vaccine | 179.4116 | 74.63024 | Dominated | 57.53829 | 23.93433 | Dominated | 36.49245 | 15.17985 | Dominated |
| | Girls-Only 1-Dose | 168.4039 | 74.72105 | Dominated | 56.65939 | 23.94532 | Dominated | 36.66575 | 15.18309 | Dominated |
| | GNV 1-Dose | 170.5004 | 74.73733 | Dominated | 58.01421 | 23.94728 | Dominated | 37.73819 | 15.18366 | Dominated |
| | Girls-Only 2-Dose | 157.1493 | 74.78946 | - | 55.41127 | 23.95287 | - | 36.46047 | 15.18514 | - |
| | GNV 2-Dose | 159.2648 | 74.81598 | 80 | 57.22536 | 23.95595 | 589 | 37.94663 | 15.18602 | 1,685 |
| Medium Coverage | No Vaccine | 179.4116 | 74.6302 | Dominated | 57.53829 | 23.93433 | Dominated | 36.49245 | 15.17985 | - |
| | Girls-Only 1-Dose | 157.8736 | 74.8151 | Dominated | 55.98209 | 23.95689 | Dominated | 37.00368 | 15.18654 | Dominated |
| | GNV 1-Dose | 164.5298 | 74.8395 | Dominated | 59.24473 | 23.95991 | Dominated | 39.46153 | 15.18745 | Dominated |
| | Girls-Only 2-Dose | 125.0681 | 75.0240 | - | 52.54066 | 23.98017 | - | 36.61105 | 15.19294 | 9 |
| | GNV 2-Dose | 136.8820 | 75.0598 | 330 | 58.12686 | 23.98455 | 1,277 | 40.77619 | 15.19424 | 3,197 |
| High Coverage | No Vaccine | 179.4116 | 74.6302 | Dominated | 57.53829 | 23.93433 | Dominated | 36.49245 | 15.17985 | - |
| | Girls-Only 1-Dose | 155.1515 | 74.8419 | Dominated | 55.85785 | 23.96029 | Dominated | 37.15061 | 15.18758 | Dominated |
| | GNV 1-Dose | 163.6281 | 74.8670 | Dominated | 59.79840 | 23.96344 | Dominated | 40.08851 | 15.18854 | Dominated |
| | Girls-Only 2-Dose | 112.3672 | 75.1281 | - | 51.62934 | 23.99260 | - | 36.91830 | 15.19655 | 25 |
| | GNV 2-Dose | 132.5022 | 75.1532 | 803 | 59.70711 | 23.99595 | 2,411 | 42.70305 | 15.19762 | 5,437 |

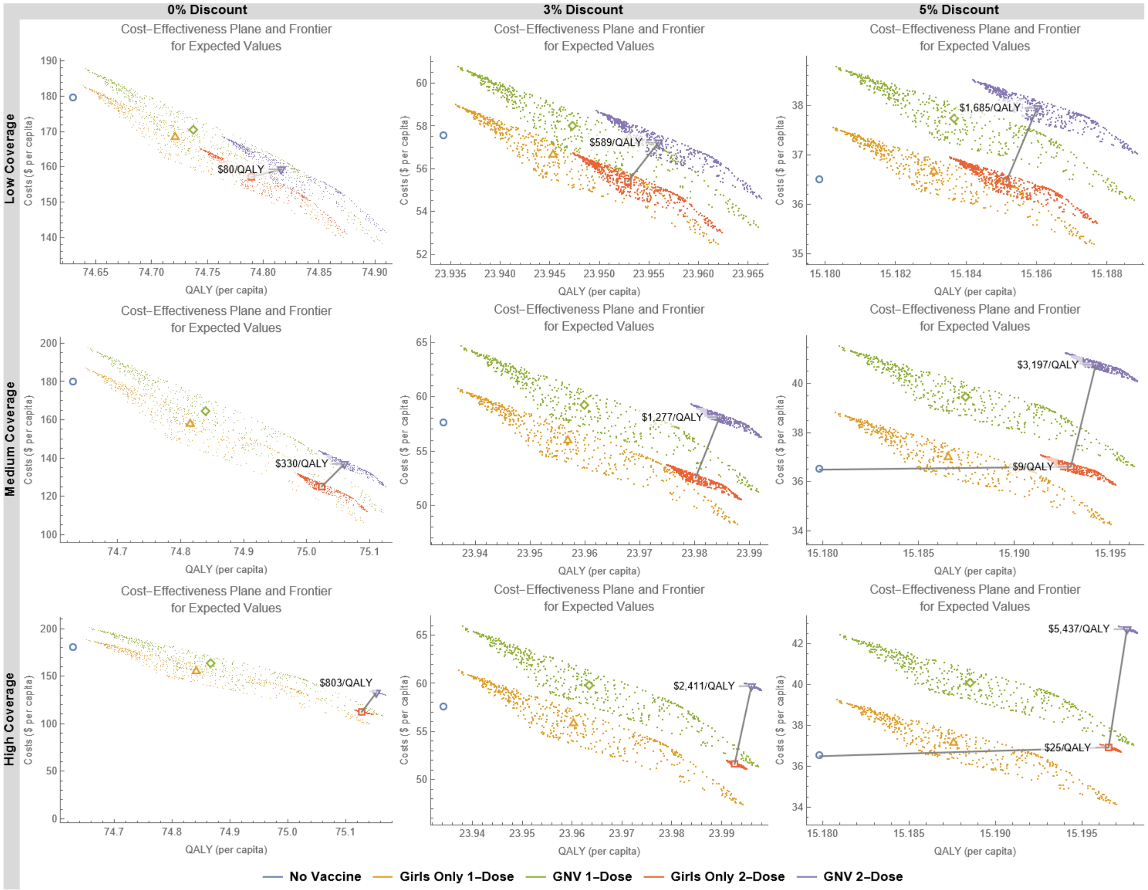


*Figure 56: Discount rate sensitivity of cost-effectiveness plane showing expected value frontier*

### G.2.1.3. Treatment cost variation

Figure 58 shows the cost-effectiveness acceptability curves for low treatment cost (all treatment costs reduced by 20%), base case treatment cost, and high treatment cost (all treatment costs increased by 20%). Within each coverage scenario, there is no significant difference in the cost-effectiveness acceptability curves under variations of treatment cost. The cost-effectiveness of expected values for variations in treatment cost is shown in Appendix G Table 59. For all coverage and treatment cost scenarios, 1-dose strategies are dominated, girls-only 2-dose strategy is cost-saving, and the ICER of GNV 2-dose strategy is not very sensitive within coverage scenarios (±$51/QALY compared to the base treatment cost).

The cost-effectiveness of expected values is not very sensitive to variations in treatment costs shown in Table 60. For all coverage scenarios, 1-dose strategies are dominated, girls only 2-dose is cost-saving, and the GNV 2-dose strategy is not very sensitive with ICER variations within coverage scenarios of ±$50/QALY

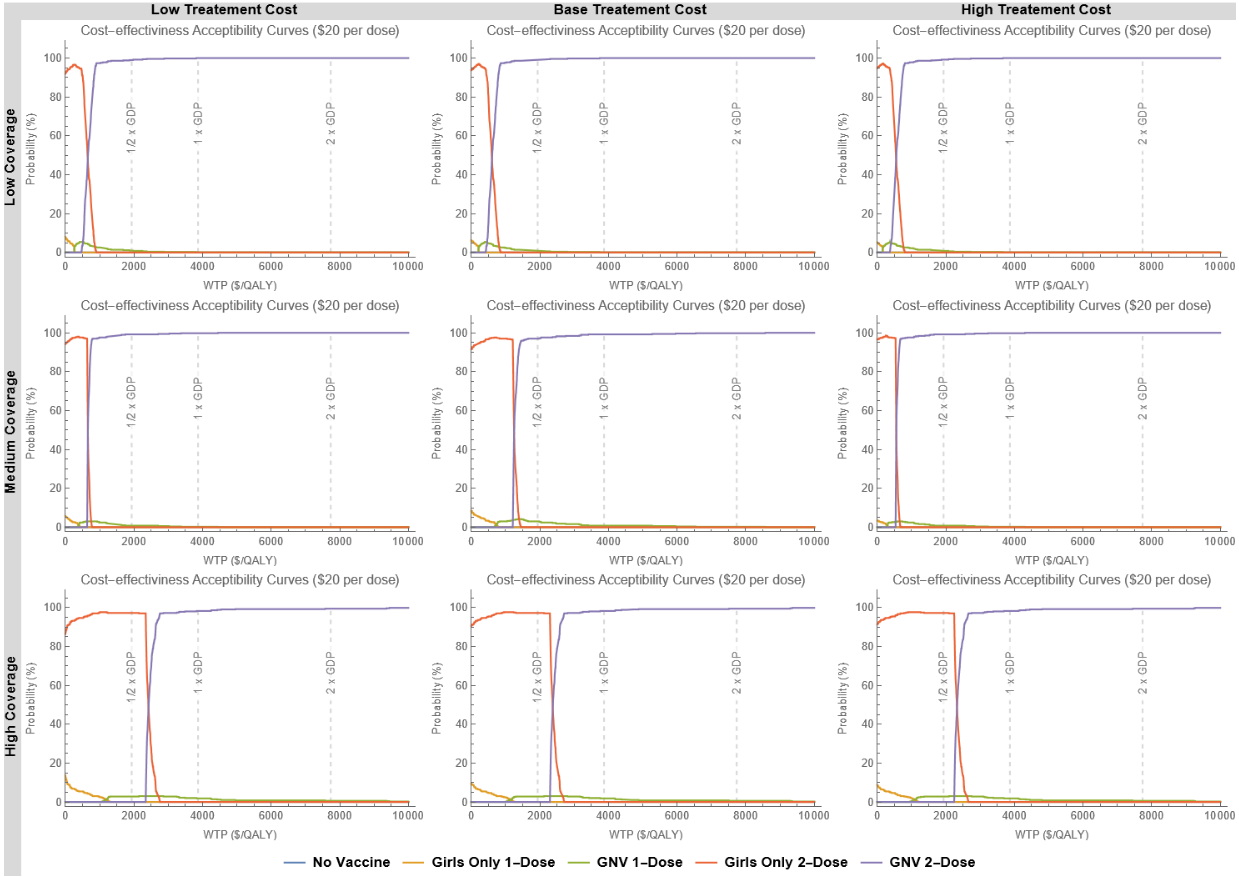


*Figure 57: Cost-effectiveness acceptability sensitivity to treatment cost*

*Table 60: Treatment cost variation sensitivity of cost-effectiveness frontier for expected values of costs and QALYs for each coverage scenario. ICERs are rounded to nearest $.*

| | Strategy | Low Treatment Costs | | | Base-case Treatment Costs | | | High Treatment Costs | | |
|---|---|---|---|---|---|---|---|---|---|---|
| | | Cost | QALYs | ICER (ΔC/ΔQ) | Cost | QALYs | ICER (ΔC/ΔQ) | Cost | QALYs | ICER (ΔC/ΔQ) |
| Low Coverage | No Vaccine | 46.03086 | 23.93433 | Dominated | 57.53829 | 23.93433 | Dominated | 69.04532 | 23.93433 | Dominated |
| | GO 1-Dose | 45.67191 | 23.94532 | Dominated | 56.65939 | 23.94532 | Dominated | 67.64651 | 23.94532 | Dominated |
| | GNV 1-Dose | 47.12247 | 23.94728 | Dominated | 58.01421 | 23.94728 | Dominated | 68.90559 | 23.94728 | Dominated |
| | GO 2-Dose | 44.81453 | 23.95287 | - | 55.41127 | 23.95287 | - | 66.00768 | 23.95287 | - |
| | GNV 2-Dose | 46.78285 | 23.95595 | 639 | 57.22536 | 23.95595 | 589 | 67.66755 | 23.95595 | 539 |
| Medium Coverage | No Vaccine | 46.03086 | 23.93433 | Dominated | 57.53829 | 23.93433 | Dominated | 69.04532 | 23.93433 | Dominated |
| | GO 1-Dose | 45.53798 | 23.95689 | Dominated | 55.98209 | 23.95689 | Dominated | 66.42589 | 23.95689 | Dominated |
| | GNV 1-Dose | 48.94939 | 23.95991 | Dominated | 59.24473 | 23.95991 | Dominated | 69.53976 | 23.95991 | Dominated |
| | GO 2-Dose | 43.28872 | 23.98017 | - | 52.54066 | 23.98017 | - | 61.79239 | 23.98017 | - |
| | GNV 2-Dose | 49.09585 | 23.98455 | 1,328 | 58.12686 | 23.98455 | 1,277 | 67.15766 | 23.98455 | 1,227 |
| High Coverage | No Vaccine | 46.03086 | 23.93433 | Dominated | 57.53829 | 23.93433 | Dominated | 69.04532 | 23.93433 | Dominated |
| | GO 1-Dose | 45.57186 | 23.96029 | Dominated | 55.85785 | 23.96029 | Dominated | 66.14353 | 23.96029 | Dominated |
| | GNV 1-Dose | 49.66760 | 23.96344 | Dominated | 59.79840 | 23.96344 | Dominated | 69.92890 | 23.96344 | Dominated |
| | GO 2-Dose | 42.97702 | 23.99260 | - | 51.62934 | 23.99260 | - | 60.28150 | 23.99260 | - |
| | GNV 2-Dose | 51.22207 | 23.99595 | 2,461 | 59.70711 | 23.99595 | 2,411 | 68.19197 | 23.99595 | 2,361 |

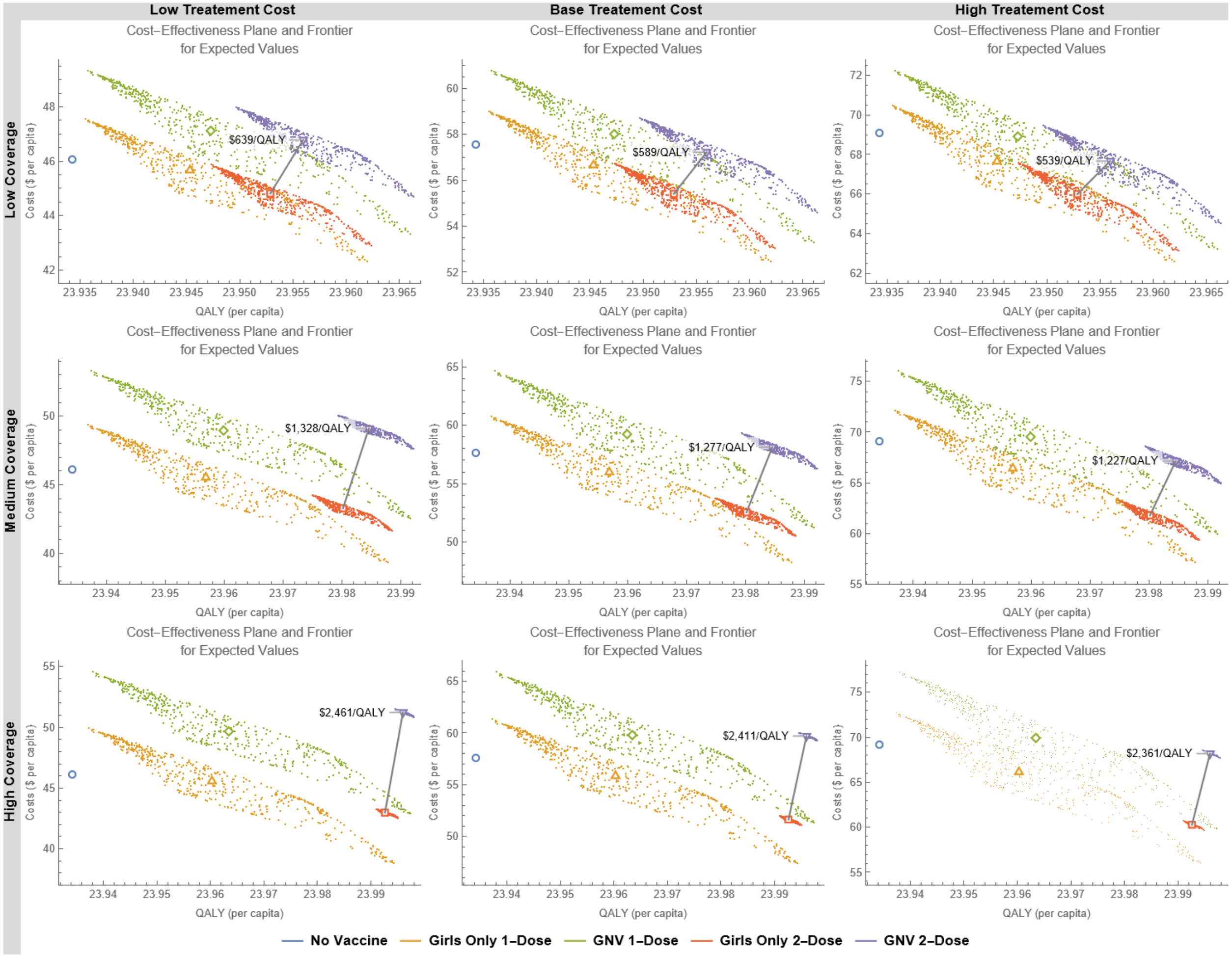


*Figure 58: Treatment cost sensitivity of cost-effectiveness plane showing expected value frontier*

### G.2.1.4. Health state utility variation

Figure 60 shows the cost-effectiveness acceptability curves for low (all health state utilities set to their lowest value), base case, and high (all health state utilities set to their highest value) health state utilities. Similar to treatment cost variations, within each coverage scenario there is no substantial difference in the cost-effectiveness acceptability curves for variations in health state utilities. The cost-effectiveness of expected values is shown in Appendix G Table 60. For all coverage scenarios, 1-dose strategies are dominated, girls-only 2-dose is cost-saving, and the GNV 2-dose strategy is not very sensitive to changes in health state utilities, with ICER ranging from $558 to $613, $1,200 to $1,340, and $2,256 to $2,543 for low, medium, and high coverage scenarios, respectively.

The cost-effectiveness of expected values is not very sensitive to variations in health state utilities shown in table 61. For all coverage scenarios, 1-dose strategies are dominated, girls only 2-dose is cost-saving, and the GNV 2-dose strategy is not very sensitive with ICER from $558 to $613, $1,200 to $1,340, $2,256 to $2,543 for low, medium, and high coverage scenarios, respectively.

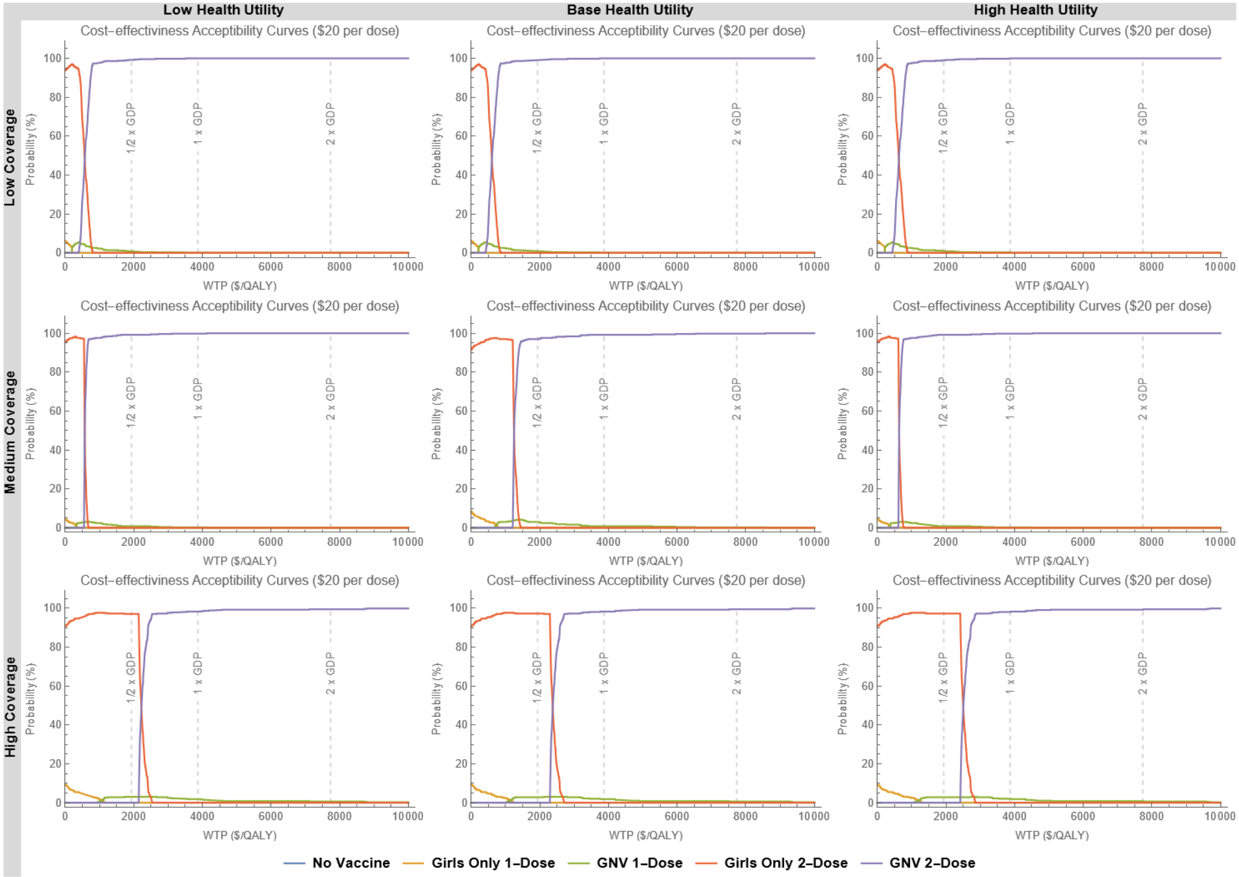


*Figure 59: Cost-effectiveness acceptability sensitivity to health state utilities*

*Table 61: Health utility variation sensitivity of cost-effectiveness frontier for expected values of costs and QALYs for each coverage scenario. ICERs are rounded to nearest $.*

| | Strategy | Low Utility | | | Base-case Utility | | | High Utility | | |
|---|---|---|---|---|---|---|---|---|---|---|
| | | Cost | QALYs | ICER (ΔC/ΔQ) | Cost | QALYs | ICER (ΔC/ΔQ) | Cost | QALYs | ICER (ΔC/ΔQ) |
| Low Coverage | No Vaccine | 57.53829 | 23.92296 | Dominated | 57.53829 | 23.93433 | Dominated | 57.53829 | 23.94314 | Dominated |
| | Girls-Only 1-Dose | 56.65939 | 23.93446 | Dominated | 56.65939 | 23.94532 | Dominated | 56.65939 | 23.95379 | Dominated |
| | GNV 1-Dose | 58.01421 | 23.93653 | Dominated | 58.01421 | 23.94728 | Dominated | 58.01421 | 23.95567 | Dominated |
| | Girls-Only 2-Dose | 55.41127 | 23.94238 | - | 55.41127 | 23.95287 | - | 55.41127 | 23.96110 | - |
| | GNV 2-Dose | 57.22536 | 23.94564 | 558 | 57.22536 | 23.95595 | 589 | 57.22536 | 23.96406 | 613 |
| Medium Coverage | No Vaccine | 57.53829 | 23.92296 | Dominated | 57.53829 | 23.93433 | Dominated | 57.53829 | 23.94314 | Dominated |
| | Girls-Only 1-Dose | 55.98209 | 23.94657 | Dominated | 55.98209 | 23.95689 | Dominated | 55.98209 | 23.96500 | Dominated |
| | GNV 1-Dose | 59.24473 | 23.94976 | Dominated | 59.24473 | 23.95991 | Dominated | 59.24473 | 23.96790 | Dominated |
| | Girls-Only 2-Dose | 52.54066 | 23.97100 | - | 52.54066 | 23.98017 | - | 52.54066 | 23.98755 | - |
| | GNV 2-Dose | 58.12686 | 23.97565 | 1,200 | 58.12686 | 23.98455 | 1,277 | 58.12686 | 23.99172 | 1,340 |
| High Coverage | No Vaccine | 57.53829 | 23.92296 | Dominated | 57.53829 | 23.93433 | Dominated | 57.53829 | 23.94314 | Dominated |
| | Girls-Only 1-Dose | 55.85785 | 23.95013 | Dominated | 55.85785 | 23.96029 | Dominated | 55.85785 | 23.96830 | Dominated |
| | GNV 1-Dose | 59.79840 | 23.95346 | Dominated | 59.79840 | 23.96344 | Dominated | 59.79840 | 23.97131 | Dominated |

| | | | | | | | | | | |
|---|---|---|---|---|---|---|---|---|---|---|
| | Girls-Only 2-Dose | 51.62934 | 23.98403 | - | 51.62934 | 23.99260 | - | 51.62934 | 23.99957 | - |
| | GNV 2-Dose | 59.70711 | 23.98761 | 2,256 | 59.70711 | 23.99595 | 2,411 | 59.70711 | 24.00275 | 2,543 |

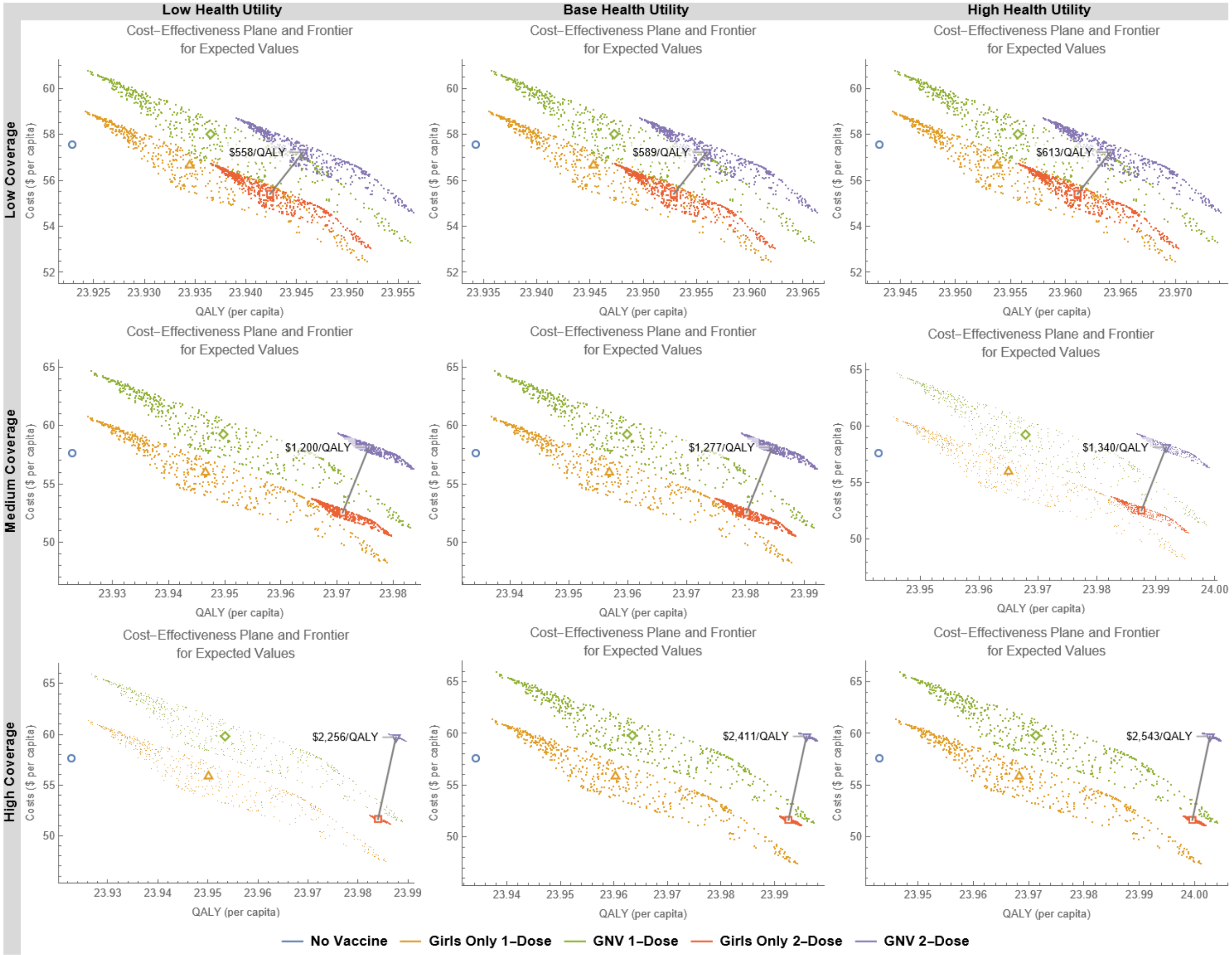


*Figure 60: Health state utility sensitivity of cost-effectiveness plane showing expected value frontier*

## G.2.2. Alternative Distribution

We also performed all the above sensitivity analyses using the alternative distributions (the ones that used both the KEN SHE and the IARC data). In summary, results in Figures 62-69 and Tables 62 to 65 indicate that, in general, the probability of 1-dose programs being cost-effective is higher in terms of acceptability curves, particularly for low WTP thresholds and for scenarios with high coverage, high dose price or high discount rate.

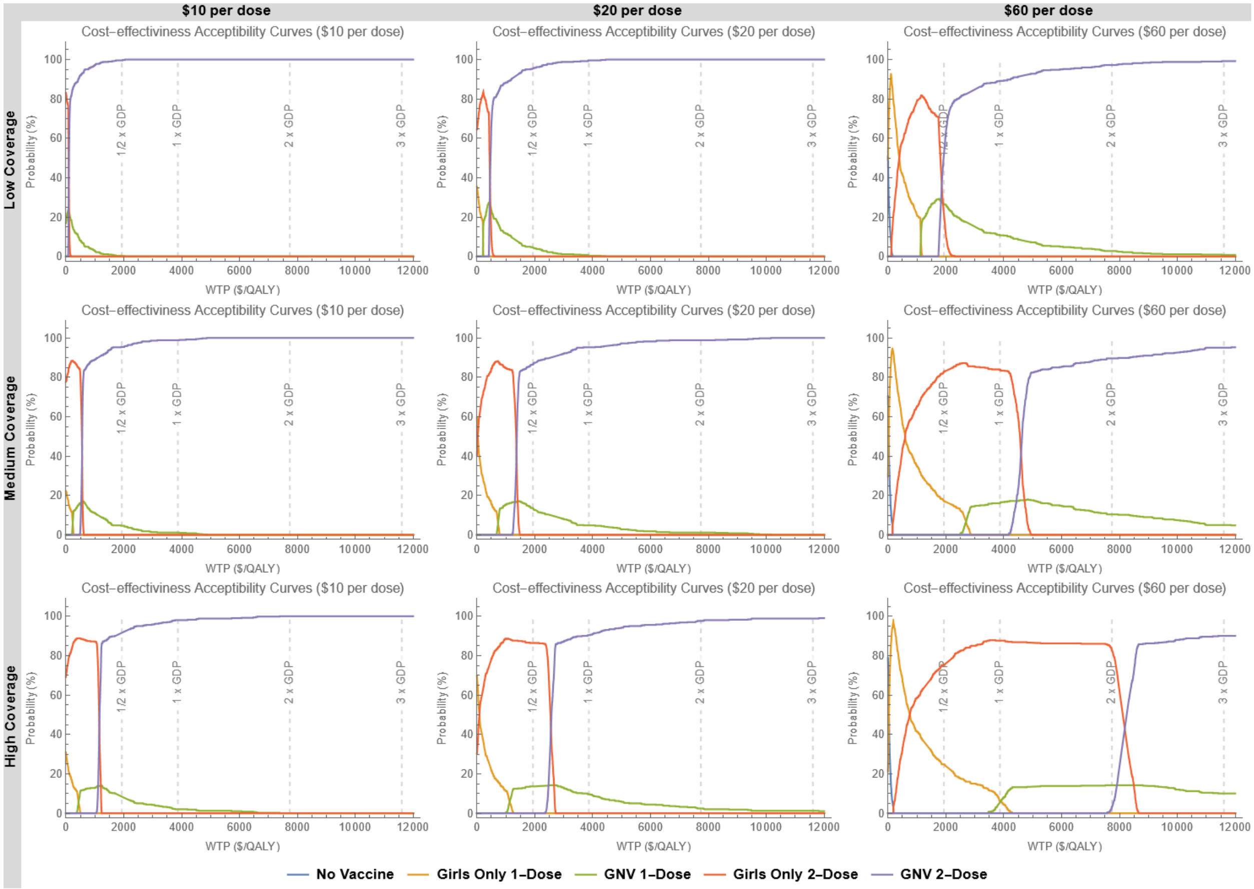


*Figure 61: Cost-effectiveness acceptability curves for alternative distribution for coverage with dose price variations*

*Table 62: Cost-effectiveness of expected values for alternative distribution with dose price variation*

| | Strategy | Low | | | Med | | | High | | |
|---|---|---|---|---|---|---|---|---|---|---|
| | | Cost | QALYs | ICER (ΔC/ΔQ) | Cost | QALYs | ICER (ΔC/ΔQ) | Cost | QALYs | ICER (ΔC/ΔQ) |
| Low Coverage | No Vaccine | 57.53829 | 23.93433 | Dominated | 57.53829 | 23.93433 | Dominated | 57.53829 | 23.93433 | - |
| | Girls only 1-Dose | 53.28514 | 23.95498 | Dominated | 54.14560 | 23.95498 | Dominated | 57.58741 | 23.95498 | 2 |
| | GNV 1-Dose | 53.33674 | 23.95841 | Dominated | 55.11395 | 23.95841 | Dominated | 62.22280 | 23.95841 | Dominated |
| | Girls only 2-Dose | 52.78531 | 23.95832 | - | 53.99855 | 23.95832 | - | 58.85150 | 23.95832 | 379 |
| | GNV 2-Dose | 53.13703 | 23.96205 | 94 | 55.64290 | 23.96205 | 441 | 65.66637 | 23.96205 | 1,828 |
| Medium C[illegible] | No Vaccine | 57.53829 | 23.93433 | Dominated | 57.53829 | 23.93433 | Dominated | 57.53829 | 23.93433 | Dominated |
| | Girls only 1-Dose | 49.18392 | 23.97571 | Dominated | 50.70203 | 23.97571 | - | 56.77448 | 23.97571 | - |
| | GNV 1-Dose | 50.00475 | 23.98013 | Dominated | 53.14027 | 23.98013 | Dominated | 65.68232 | 23.98013 | Dominated |

| | | | | | | | | | | |
|---|---|---|---|---|---|---|---|---|---|---|
| | Girls only 2-Dose | 48.12204 | 23.98508 | - | 50.99127 | 23.98508 | 31 | 62.46819 | 23.98508 | 608 |
| | GNV 2-Dose | 50.38687 | 23.98917 | 553 | 56.31299 | 23.98917 | 1,299 | 80.01747 | 23.98917 | 4,283 |
| High Coverage | No Vaccine | 57.53829 | 23.93433 | Dominated | 57.53829 | 23.93433 | Dominated | 57.53829 | 23.93433 | - |
| | Girls only 1-Dose | 48.19171 | 23.98151 | Dominated | 50.40513 | 23.98151 | - | 59.25880 | 23.98151 | 36 |
| | GNV 1-Dose | 49.48098 | 23.98574 | Dominated | 54.05266 | 23.98574 | Dominated | 72.33937 | 23.98574 | Dominated |
| | Girls only 2-Dose | 47.10402 | 23.99404 | - | 51.28738 | 23.99404 | 70 | 68.02082 | 23.99404 | 699 |
| | GNV 2-Dose | 50.77845 | 23.99719 | 1,167 | 59.41892 | 23.99719 | 2,583 | 93.98081 | 23.99719 | 8,246 |

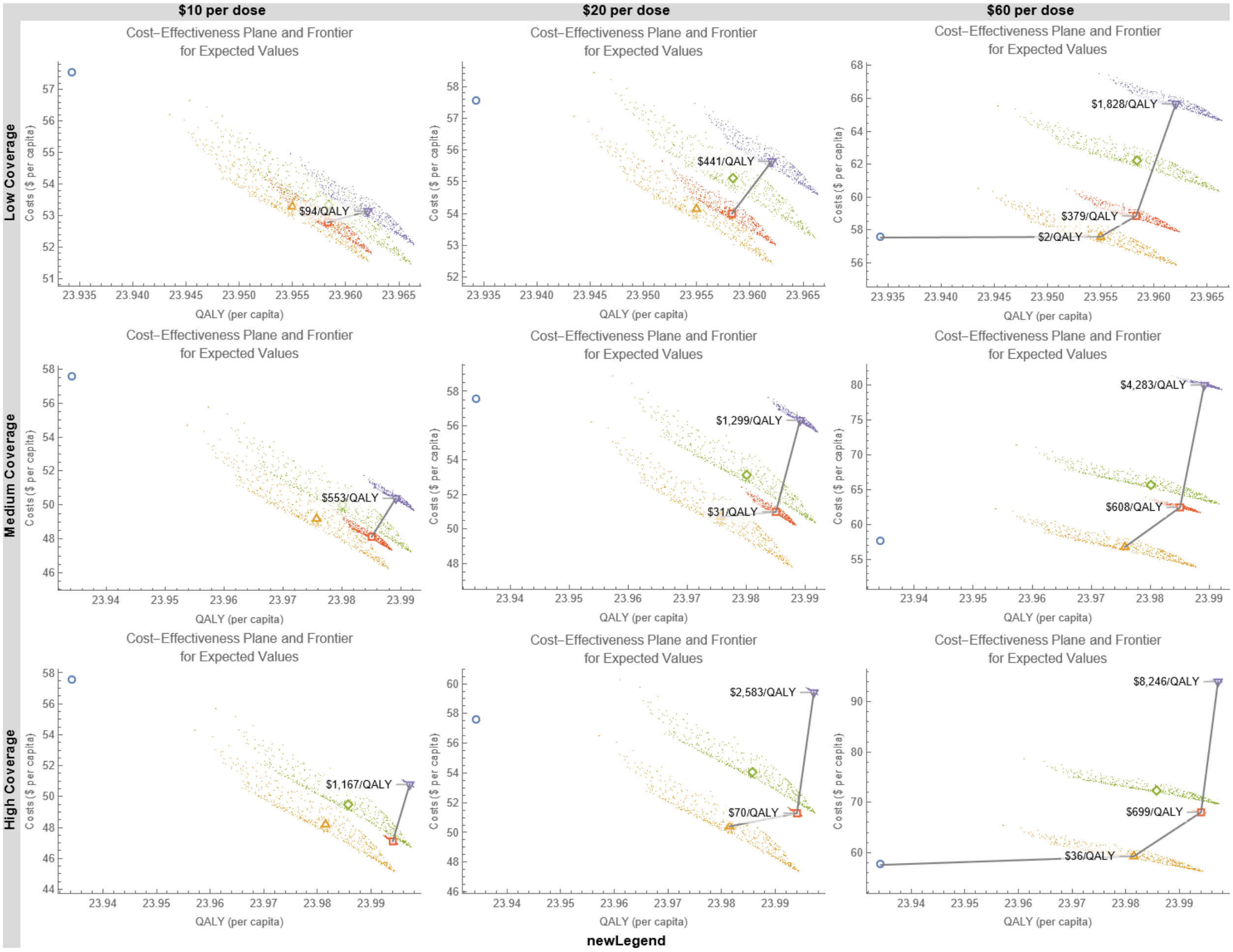


*Figure 62: Cost-effectiveness plane and frontier for alternative distribution with dose price variation*

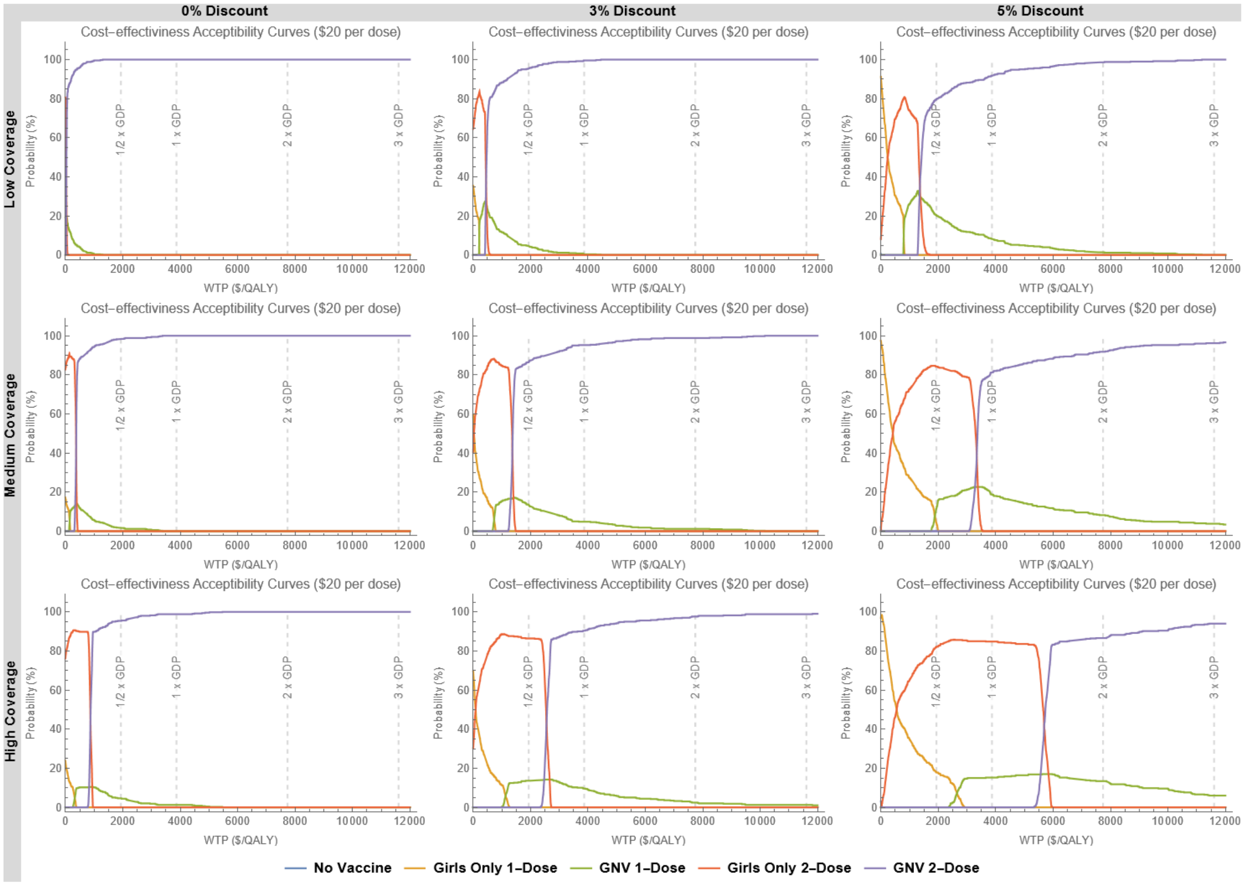


*Figure 63: Cost-effectiveness acceptability curves for alternative distribution for coverage with discount rate variation*

*Table 63: Cost-effectiveness of expected values for alternative distribution with discount variation*

| | Strategy | Low | | | Med | | | High | | |
|---|---|---|---|---|---|---|---|---|---|---|
| | | Cost | QALYs | ICER (ΔC/ΔQ | Cost | QALYs | ICER (ΔC/ΔQ) | Cost | QALYs | ICER (ΔC/ΔQ) |
| Low Coverage | No Vaccine | 179.4116 | 74.63024 | Dominated | 57.53829 | 23.93433 | Dominated | 36.49245 | 15.17985 | Dominated |
| | Girls only 1-Dose | 151.1810 | 74.80504 | Dominated | 54.14560 | 23.95498 | Dominated | 36.06333 | 15.18581 | - |
| | GNV 1-Dose | 149.5001 | 74.83426 | Dominated | 55.11395 | 23.95841 | Dominated | 37.33932 | 15.18679 | Dominated |
| | Girls only 2-Dose | 147.4523 | 74.83666 | - | 53.99855 | 23.95832 | - | 36.39004 | 15.18668 | 374 |
| | GNV 2-Dose | 147.7443 | 74.86879 | 9 | 55.64290 | 23.96205 | 441 | 38.29348 | 15.18775 | 1,784 |
| Medium Coverage | No Vaccine | 179.4116 | 74.63024 | Dominated | 57.53829 | 23.92296 | Dominated | 36.49245 | 15.17985 | Dominated |
| | Girls only 1-Dose | 126.9617 | 74.97747 | Dominated | 50.70203 | 23.96632 | - | 36.28615 | 15.19188 | - |
| | GNV 1-Dose | 131.1452 | 75.01361 | Dominated | 53.14027 | 23.97101 | Dominated | 39.75582 | 15.19320 | Dominated |
| | Girls only 2-Dose | 117.4795 | 75.06516 | - | 50.99127 | 23.97614 | 29 | 38.50738 | 15.19436 | 896 |

| | | | | | | | | | | |
|---|---|---|---|---|---|---|---|---|---|---|
| | GNV 2-Dose | 129.8713 | 75.09795 | 378 | 56.31299 | 23.98052 | 1,217 | 45.21217 | 15.19560 | 5,401 |
| High Coverage | No Vaccine | 179.4116 | 74.63024 | Dominated | 57.53829 | 23.93433 | Dominated | 36.49245 | 15.17985 | Dominated |
| | Girls only 1-Dose | 115.2212 | 75.02388 | Dominated | 50.40513 | 23.98151 | - | 35.12640 | 15.19363 | - |
| | GNV 1-Dose | 115.9965 | 75.05781 | Dominated | 54.05266 | 23.98574 | Dominated | 37.95652 | 15.19491 | Dominated |
| | Girls only 2-Dose | 100.1023 | 75.13961 | - | 51.28738 | 23.99404 | 70 | 36.78159 | 15.19699 | 493 |
| | GNV 2-Dose | 109.6008 | 75.16278 | 410 | 59.41892 | 23.99719 | 2,583 | 42.58457 | 15.19800 | 5,740 |

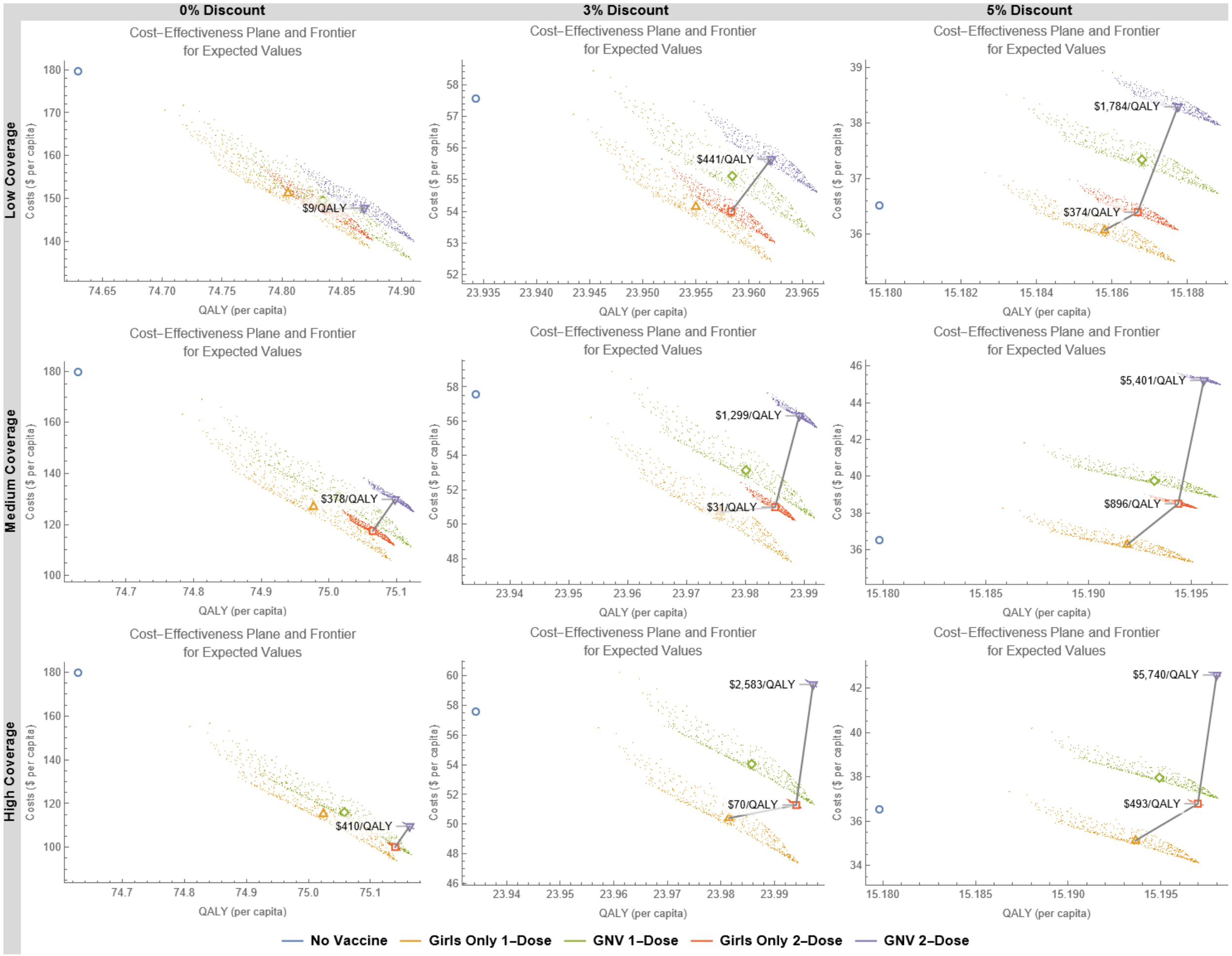


*Figure 64: Cost-effectiveness plane and frontier for alternative distribution with discount rate variation*

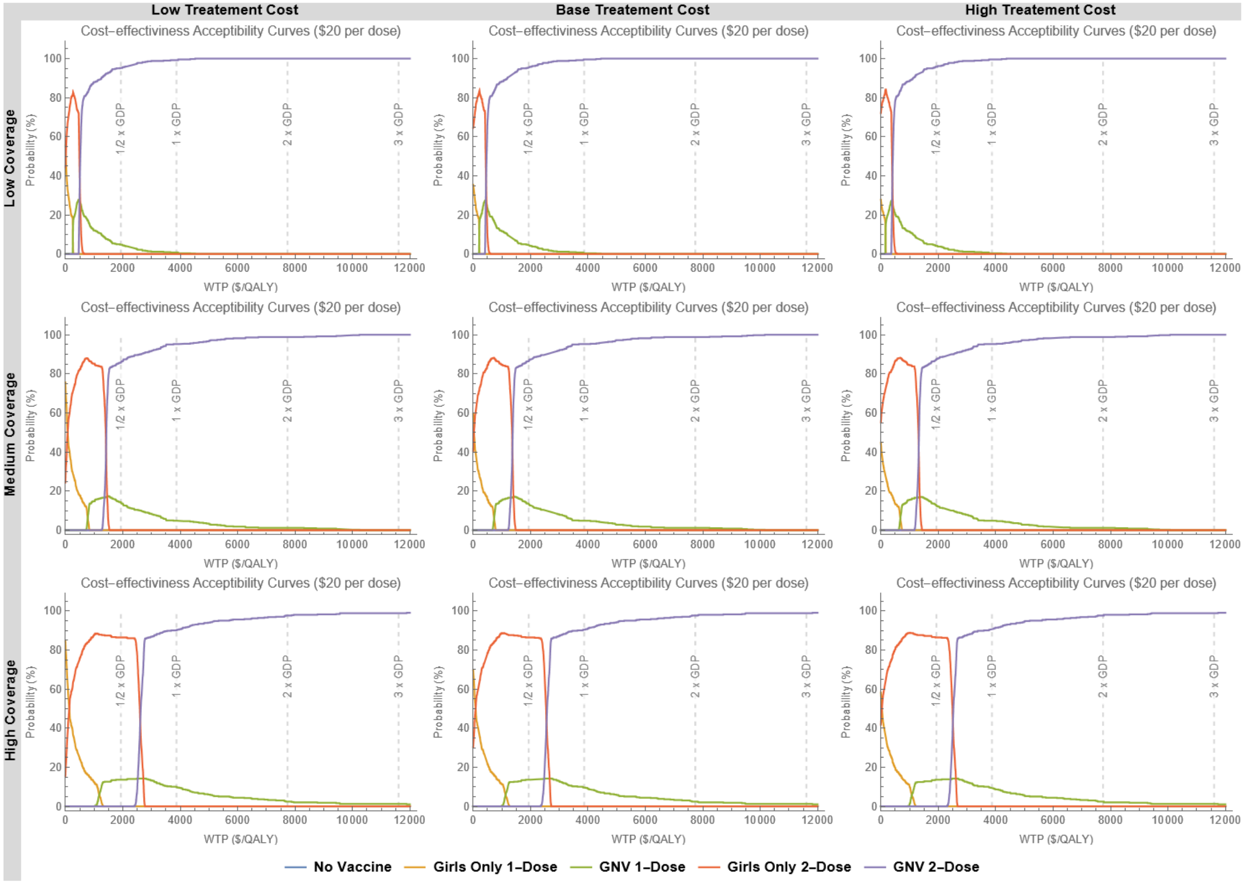


*Figure 65: Cost-effectiveness acceptability curves for alternative distribution for coverage with treatment cost variation*

*Table 64: Cost-effectiveness of expected values for alternative distribution with treatment cost variation*

| | Strategy | Low | | | Med | | | High | | |
|---|---|---|---|---|---|---|---|---|---|---|
| | | Cost | QALYs | ICER (ΔC/ΔQ) | Cost | QALYs | ICER (ΔC/ΔQ) | Cost | QALYs | ICER (ΔC/ΔQ) |
| Low Coverage | No Vaccine | 46.03086 | 23.93433 | Dominated | 57.53829 | 23.93433 | Dominated | 69.04532 | 23.93433 | Dominated |
| | Girls only 1-Dose | 43.66088 | 23.95498 | - | 54.14560 | 23.95498 | Dominated | 64.63000 | 23.95498 | Dominated |
| | GNV 1-Dose | 44.80226 | 23.95841 | Dominated | 55.11395 | 23.95841 | Dominated | 65.42534 | 23.95841 | Dominated |
| | Girls only 2-Dose | 43.68435 | 23.95832 | 7 | 53.99855 | 23.95832 | - | 64.31245 | 23.95832 | - |
| | GNV 2-Dose | 45.51688 | 23.96205 | 491 | 55.64290 | 23.96205 | 441 | 65.76863 | 23.96205 | 391 |
| Medium Coverage | No Vaccine | 46.03086 | 23.93433 | Dominated | 57.53829 | 23.93433 | Dominated | 69.04532 | 23.93433 | Dominated |
| | Girls only 1-Dose | 41.22984 | 23.97571 | - | 50.70203 | 23.97571 | - | 60.17400 | 23.97571 | Dominated |
| | GNV 1-Dose | 43.89213 | 23.98013 | Dominated | 53.14027 | 23.98013 | Dominated | 62.38818 | 23.98013 | Dominated |

| | | | | | | | | | | |
|---|---|---|---|---|---|---|---|---|---|---|
| | Girls only 2-Dose | 41.98634 | 23.98508 | 81 | 50.99127 | 23.98508 | 31 | 59.99601 | 23.98508 | - |
| | GNV 2-Dose | 47.51489 | 23.98917 | 1,349 | 56.31299 | 23.98917 | 1,299 | 65.11090 | 23.98917 | 1,248 |
| High Coverage | No Vaccine | 46.03086 | 23.93433 | Dominated | 57.53829 | 23.93433 | Dominated | 69.04532 | 23.93433 | Dominated |
| | Girls only 1-Dose | 41.20967 | 23.98151 | - | 50.40513 | 23.98151 | - | 59.60037 | 23.98151 | - |
| | GNV 1-Dose | 45.07099 | 23.98574 | Dominated | 54.05266 | 23.98574 | Dominated | 63.03411 | 23.98574 | Dominated |
| | Girls only 2-Dose | 42.70345 | 23.99404 | 119 | 51.28738 | 23.99404 | 70 | 59.87115 | 23.99404 | 22 |
| | GNV 2-Dose | 50.99152 | 23.99719 | 2,633 | 59.41892 | 23.99719 | 2,583 | 67.84616 | 23.99719 | 2,533 |

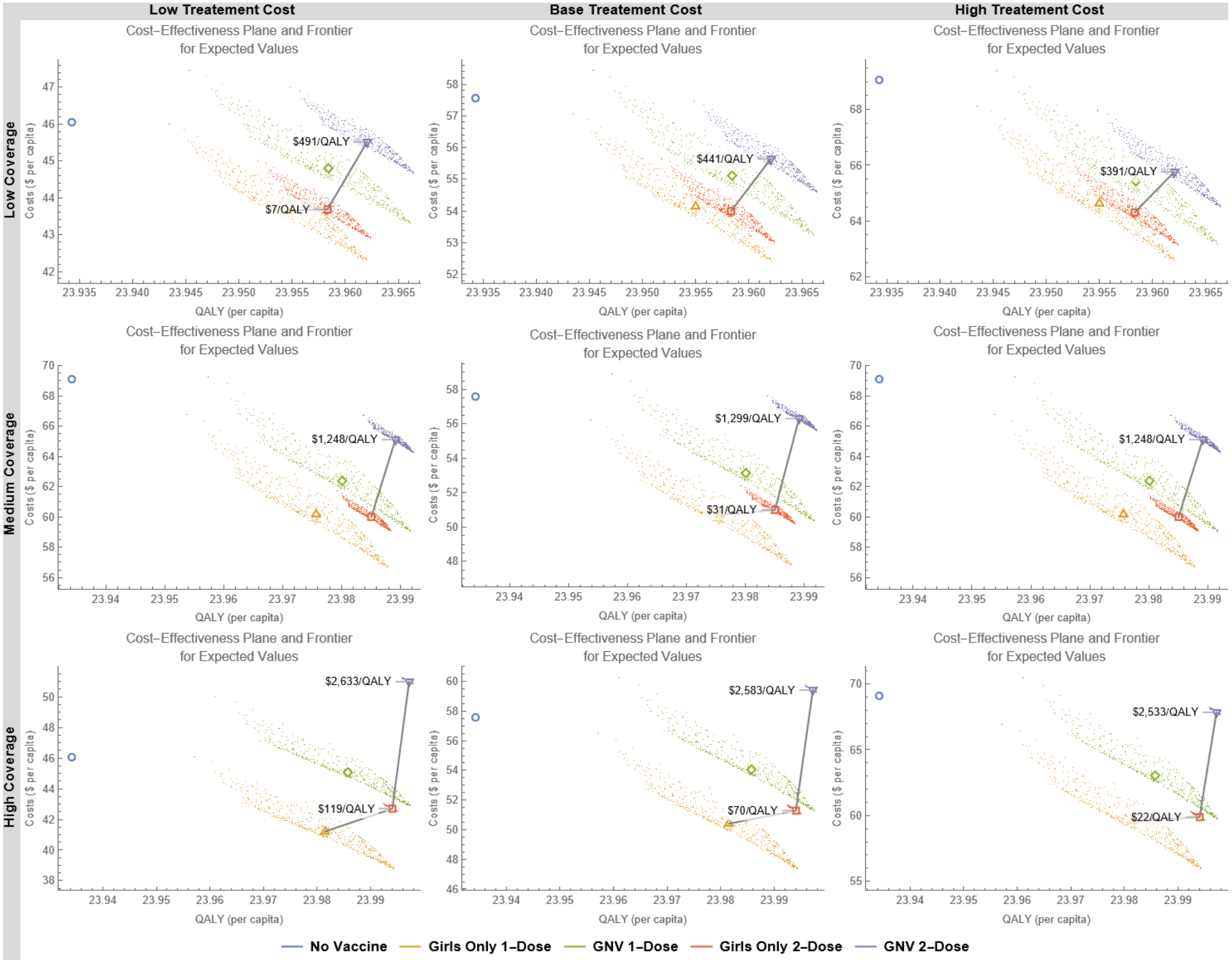


*Figure 66: Cost-effectiveness plane and frontier for alternative distribution with treatment cost variation*

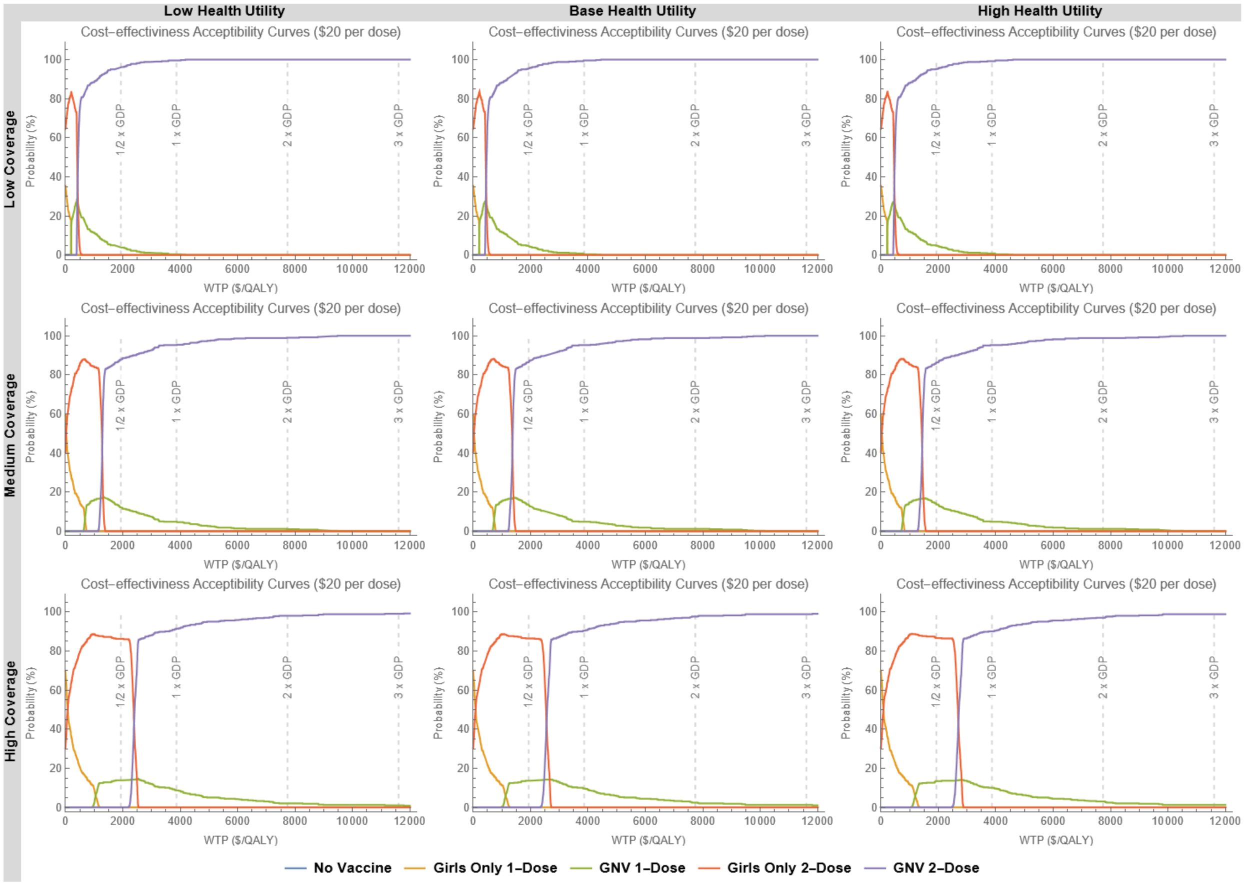


*Figure 67: Cost-effectiveness acceptability curves for alternative distribution for coverage with health state utility variation*

*Table 65: Cost-effectiveness of expected values for alternative distribution with health state utility variation*

| | Strategy | Low | | | Med | | | High | | |
|---|---|---|---|---|---|---|---|---|---|---|
| | | Cost | QALYs | ICER (ΔC/ΔQ) | Cost | QALYs | ICER (ΔC/ΔQ) | Cost | QALYs | ICER (ΔC/ΔQ) |
| Low Coverage | No Vaccine | 57.53829 | 23.92296 | Dominated | 57.53829 | 23.93433 | Dominated | 57.53829 | 23.94314 | Dominated |
| | Girls only 1-Dose | 54.14560 | 23.94460 | Dominated | 54.14560 | 23.95498 | Dominated | 54.14560 | 23.96315 | Dominated |
| | GNV 1-Dose | 55.11395 | 23.94822 | Dominated | 55.11395 | 23.95841 | Dominated | 55.11395 | 23.96644 | Dominated |
| | Girls only 2-Dose | 53.99855 | 23.94810 | - | 53.99855 | 23.95832 | - | 53.99855 | 23.96638 | - |
| | GNV 2-Dose | 55.64290 | 23.95204 | 417 | 55.64290 | 23.96205 | 441 | 55.64290 | 23.96996 | 459 |
| Medium Coverage | No Vaccine | 57.53829 | 23.92296 | Dominated | 57.53829 | 23.93433 | Dominated | 57.53829 | 23.94314 | Dominated |
| | Girls only 1-Dose | 50.70203 | 23.96632 | - | 50.70203 | 23.97571 | - | 50.70203 | 23.98324 | - |
| | GNV 1-Dose | 53.14027 | 23.97101 | Dominated | 53.14027 | 23.98013 | Dominated | 53.14027 | 23.98746 | Dominated |

| | | | | | | | | | | |
|---|---|---|---|---|---|---|---|---|---|---|
| | Girls only 2-Dose | 50.99127 | 23.97614 | 29 | 50.99127 | 23.98508 | 31 | 50.99127 | 23.99230 | 32 |
| | GNV 2-Dose | 56.31299 | 23.98052 | 1,217 | 56.31299 | 23.98917 | 1,299 | 56.31299 | 23.99619 | 1,366 |
| High Coverage | No Vaccine | 57.53829 | 23.92296 | Dominated | 57.53829 | 23.93433 | Dominated | 57.53829 | 23.94314 | Dominated |
| | Girls only 1-Dose | 50.40513 | 23.97239 | - | 50.40513 | 23.98151 | - | 50.40513 | 23.98885 | - |
| | GNV 1-Dose | 54.05266 | 23.97689 | Dominated | 54.05266 | 23.98574 | Dominated | 54.05266 | 23.99289 | Dominated |
| | Girls only 2-Dose | 51.28738 | 23.98554 | 67 | 51.28738 | 23.99404 | 70 | 51.28738 | 24.00097 | 73 |
| | GNV 2-Dose | 59.41892 | 23.98891 | 2,417 | 59.41892 | 23.99719 | 2,583 | 59.41892 | 24.00395 | 2,725 |

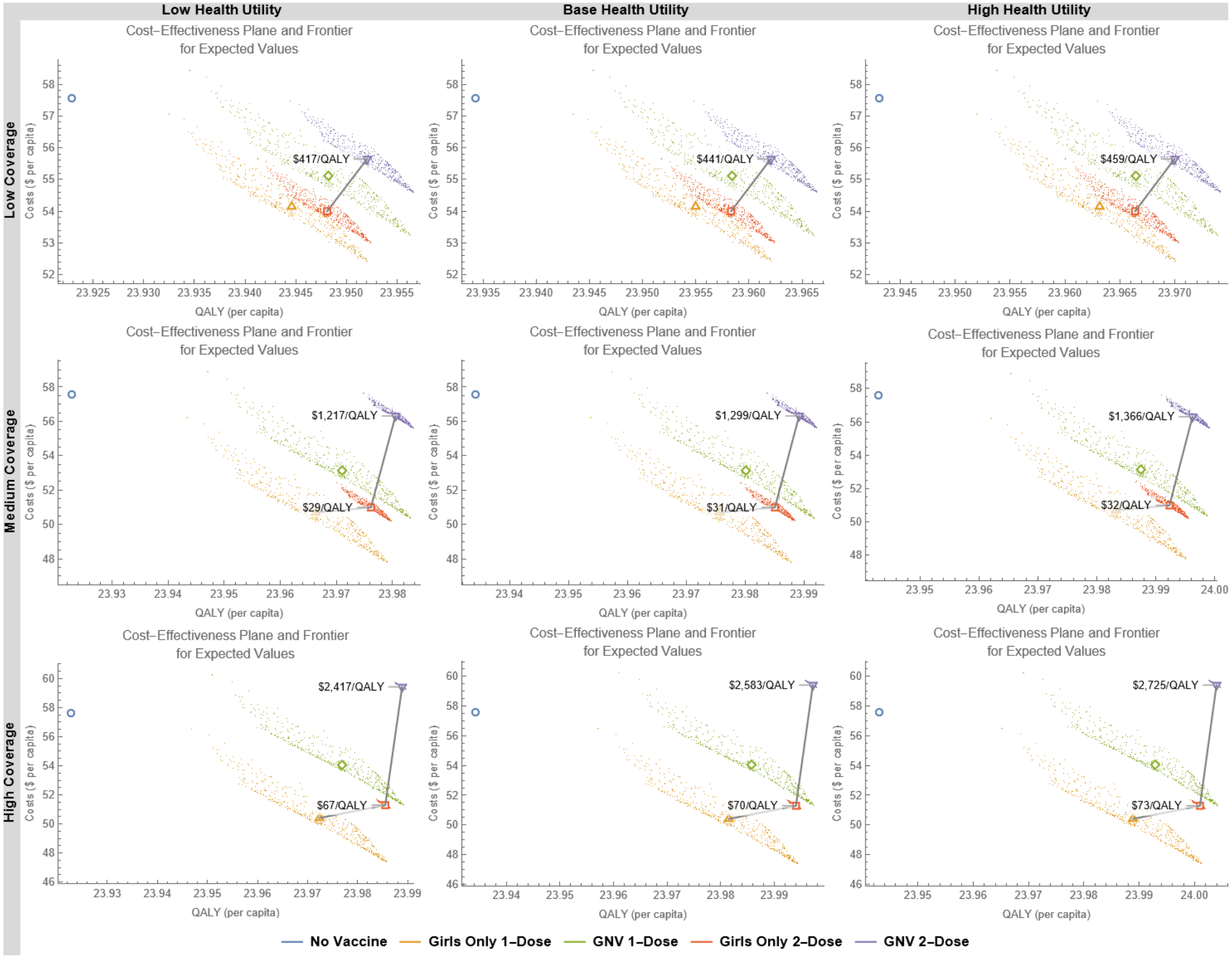


*Figure 68: Cost-effectiveness plane and frontier for alternative distribution with health state utility variation*

## G.3. Cervical cancer elimination

### G.3.1. Base Case Distribution

We assessed probability of and time to HPV-related cervical cancer disease elimination, defined as either <10 cervical cancer cases per 100,000 or <4 cervical cancer cases per 100,000. Table 66 presents the probability of the mean cervical cancer incidence reaching disease elimination within 100 years, and the time to reach it (mean, median, lower and upper across simulations), for each of the four different vaccination strategies, and by coverage scenario. In this table, a value of NA implies the value either doesn't exist (elimination is never reached for at least some samples, even after 100 years) or the value is greater than 100 years.

Consider the <10 cases per 100,000 threshold (left panel). For high coverage, GNV 2-dose and girls-only 2-dose have 100% probability of reaching elimination in 58 and 59 years, respectively, while both 1-dose scenarios have a low (16 to 19.2%) probability of reaching elimination. For medium coverage, the GNV and girls-only2-dose have a very high (99.8-100%) probability of reaching elimination after 69 and 77 years, respectively, while both 1-dose scenarios have a low (8.6 to 12.6%) probability of reaching elimination. The low coverage scenarios have no probability of reaching elimination.

Consider the <4 cases per 100,000 threshold (right panel). For medium coverage, all scenarios have low probability of reaching elimination (0.2 to 13.4%). For high coverage, GNV 2-dose and girls-only 2-dose have 100% probability of reaching elimination in 73 and 77 years, respectively, while both 1-dose scenarios have a low (4.4 to 6.4%) probability of reaching elimination.

*Table 66: Probability of and time to reaching diseases elimination (defined as either <10 cases of cervical, cancer per 100,000 or <4 cases of cervical, cancer per 100,000) by coverage scenario, for each of the four different vaccination strategies.*

| | | Elimination at 10 cases/100,000 | | | | | Elimination at 4 cases/100,000 | | | | |
|---|---|---|---|---|---|---|---|---|---|---|---|
| | | Probability of reaching elimination (%) | Time to elimination (years) | | | | Probability of reaching elimination (%) | Time to elimination (years) | | | |
| | | | Mean | Median | Lower 95%CI | Upper 95%CI | | Mean | Median | Lower 95%CI | Upper 95%CI |
| Low Coverage | Girls Only 1-Dose | 0 | NA | NA | NA | NA | 0 | NA | NA | NA | NA |
| | GNV 1-Dose | 0 | NA | NA | NA | NA | 0 | NA | NA | NA | NA |
| | Girls Only 2-Dose | 0 | NA | NA | NA | NA | 0 | NA | NA | NA | NA |
| | GNV 2-Dose | 0 | NA | NA | NA | NA | 0 | NA | NA | NA | NA |
| Medium Coverag | Girls Only 1-Dose | 8.6 | NA | NA | 65 | NA | 0.2 | NA | NA | NA | NA |

| | | | | | | | | | | | |
|---|---|---|---|---|---|---|---|---|---|---|---|
| | GNV 1-Dose | 12.6 | NA | NA | 62 | NA | 2 | NA | NA | NA | NA |
| | Girls Only 2-Dose | 99.8 | 77 | 77 | 62 | 94 | 2.4 | NA | NA | NA | NA |
| | GNV 2-Dose | 100 | 69 | 70 | 60 | 76 | 13.4 | NA | NA | 82 | NA |
| High Coverage | Girls Only 1-Dose | 16 | NA | NA | 59 | NA | 4.4 | NA | NA | 81 | NA |
| | GNV 1-Dose | 19.2 | NA | NA | 58 | NA | 6.4 | NA | NA | 74 | NA |
| | Girls Only 2-Dose | 100 | 59 | 59 | 57 | 60 | 100 | 77 | 78 | 72 | 80 |
| | GNV 2-Dose | 100 | 58 | 58 | 56 | 58 | 100 | 73 | 74 | 70 | 75 |

Figures 70, 71, and 72 show the cervical cancer incidence and ranges over time for the low, medium and high coverage scenarios, respectively.

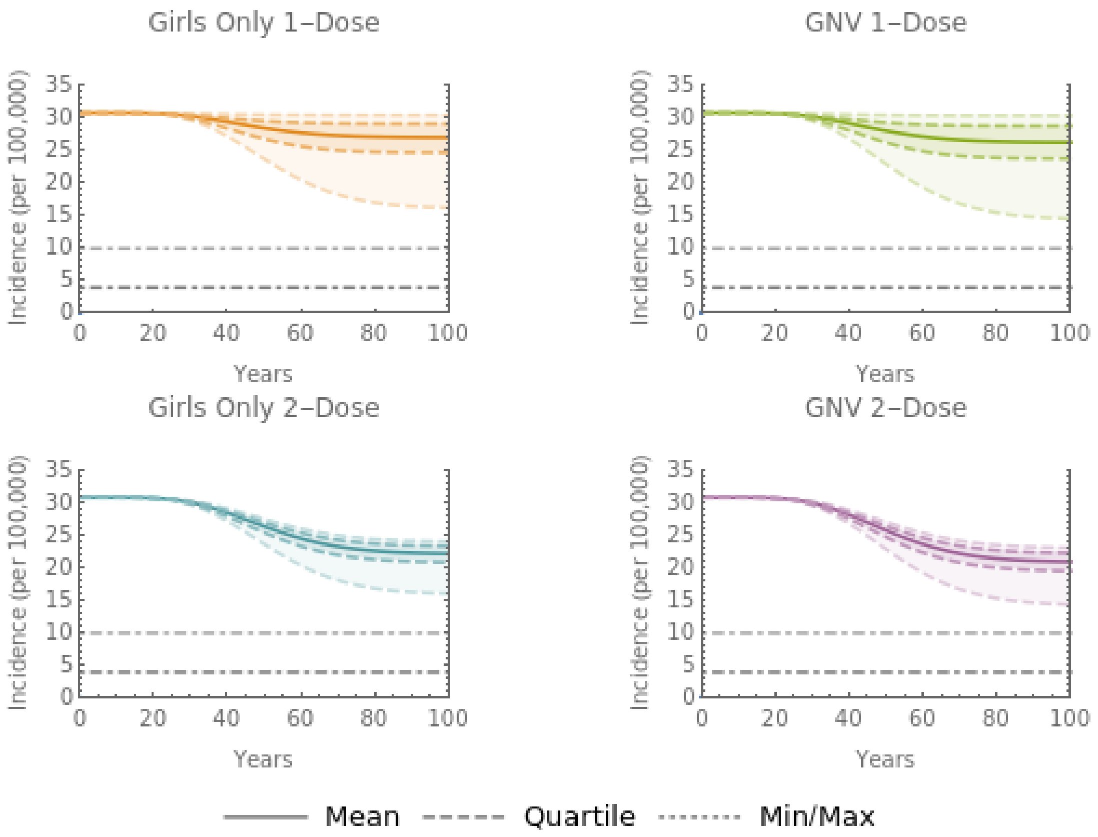


*Figure 70: Median cervical cancer incidence (all HPV types) and range of variation over time for the low coverage scenario. The darker shaded areas are the middle quartiles and the lighter shaded areas extend to the min and max. Dash Dotted lines are 4 and 10 cases per 100,000 elimination thresholds.*

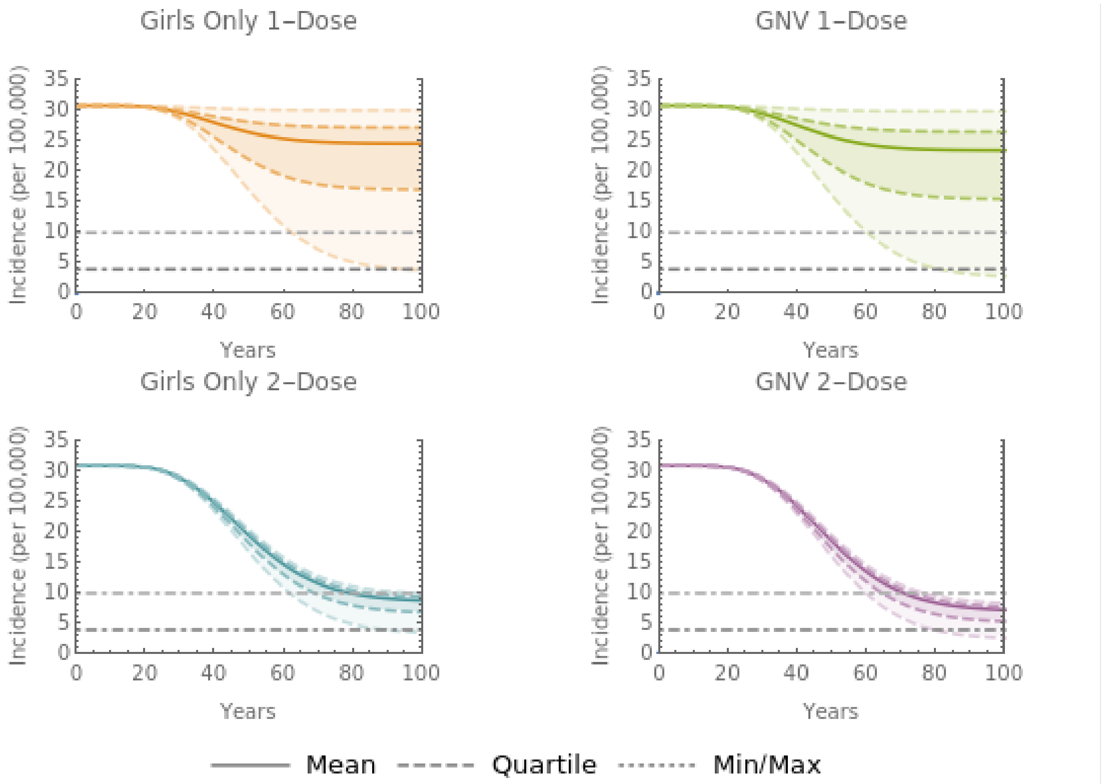


*Figure 71: Median cervical cancer incidence (all HPV types) and range of variation over time for the medium coverage scenario. The darker shaded areas are the middle quartiles and the lighter shaded areas extend to the minimum and maximum of the incidence. Dash Dotted lines are 4 and 10 cases per 100,000 elimination thresholds.*

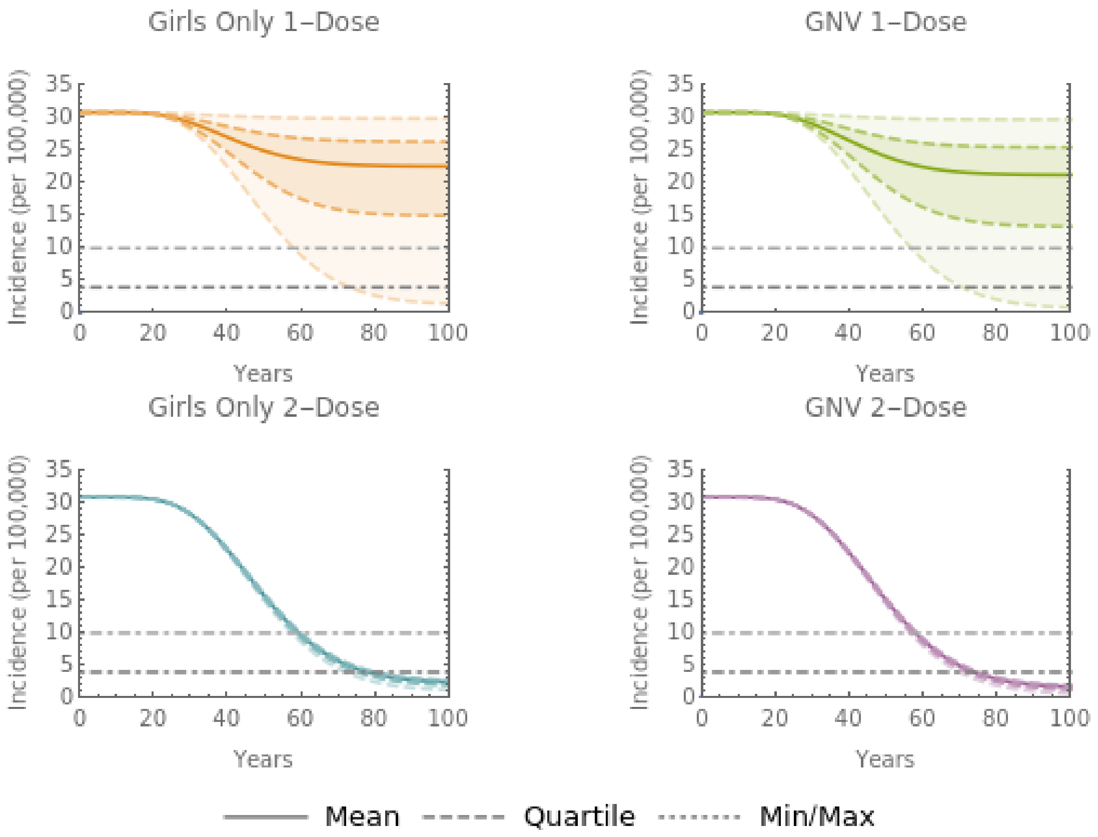


*Figure 72: Median cervical cancer incidence (all HPV types) and range of variation over time for the high coverage scenario. The darker shaded areas are the middle quartiles and the lighter shaded areas extend to the min and max. Dash Dotted lines are 4 and 10 cases per 100,000 elimination thresholds.*

## G.3.2. Alternative Distribution

Table 67 presents the probability of the mean cervical cancer incidence reaching disease elimination (defined as either <10 or <4 cases of cervical cancer per 100,000) within 100 years, and the time to reach it (mean, median, lower and upper across simulations), for each of the four different vaccination strategies and by coverage scenarios under the alternative distributions. For high coverage, GNV 2-dose and girls-only 2-dose programs behave very similarly to the base case distribution for both thresholds since these scenarios are not strongly influenced by the 1-dose properties. However, for the 1-dose programs, the probability of reaching the <10 per 100,000 threshold increases from between 16% to 19.2% with mean incidence not reaching the threshold to between 51.8% and 60.6% and with the mean incidence reaching the threshold between 82 and 87 years. For medium coverage the 2-dose programs are again very similar to the base distribution results, but the probability of 1-dose programs reaching elimination increases from between 8.6% and 12.6% to between 38.6% and 47.4% (although the mean incidence still does not reach the threshold). Low coverage scenarios have no probability of reaching the

threshold. For the <4 per 100,000 threshold and high coverage, the 1-dose programs' probability of reaching the threshold increases from between 4.4% and 6.4% to between 17.6% and 28.8% (although mean incidence does not reach the threshold). For medium coverage, both the girls-only 2-dose and GNV 2-dose increase to 9.2% (from 2.4%) and 37.8% (from 13.4%), while 1-dose the girls-only and GNV 1-dose increase to 1% (from 0.2%) and 8.2% (from 2%). The low coverage scenarios have no probability of reaching elimination.

*Table 67: Probability of and time to reaching diseases elimination (defined as either <10 cases of cervical, cancer per 100,000 or <4 cases of cervical, cancer per 100,000) by coverage scenario, for each of the four different vaccination strategies under the alternative distribution*

| | | Elimination at 10 cases/100,000 | | | | | Elimination at 4 cases/100,000 | | | | |
|---|---|---|---|---|---|---|---|---|---|---|---|
| | | Probability of reaching elimination (%) | Time to elimination (years) | | | | Probability of reaching elimination (%) | Time to elimination (years) | | | |
| | | | Mean | Median | Lower 95%CI | Upper 95%CI | | Mean | Median | Lower 95%CI | Upper 95%CI |
| Low Coverage | Girls Only 1-Dose | 0 | NA | NA | NA | NA | 0 | NA | NA | NA | NA |
| | GNV 1-Dose | 0 | NA | NA | NA | NA | 0 | NA | NA | NA | NA |
| | Girls Only 2-Dose | 0 | NA | NA | NA | NA | 0 | NA | NA | NA | NA |
| | GNV 2-Dose | 0 | NA | NA | NA | NA | 0 | NA | NA | NA | NA |
| Medium Coverage | Girls Only 1-Dose | 38.6 | NA | NA | 63 | NA | 1 | NA | NA | NA | NA |
| | GNV 1-Dose | 47.4 | NA | NA | 61 | NA | 8.2 | NA | NA | 84 | NA |
| | Girls Only 2-Dose | 100 | 66 | 66 | 62 | 74 | 9.2 | NA | NA | 92 | NA |
| | GNV 2-Dose | 100 | 63 | 63 | 60 | 68 | 37.8 | NA | NA | 80 | NA |
| High Coverage | Girls Only 1-Dose | 51.8 | 87 | 91 | 58 | NA | 17.6 | NA | NA | 74 | NA |
| | GNV 1-Dose | 60.6 | 82 | 70 | 57 | NA | 28.8 | NA | NA | 71 | NA |
| | Girls Only 2-Dose | 100 | 58 | 58 | 57 | 59 | 100 | 74 | 74 | 72 | 77 |
| | GNV 2-Dose | 100 | 57 | 57 | 56 | 57 | 100 | 71 | 71 | 70 | 73 |

Figures 73, 74, and 75 show the cervical cancer incidence and ranges over time for the low, medium and high coverage scenarios, respectively.

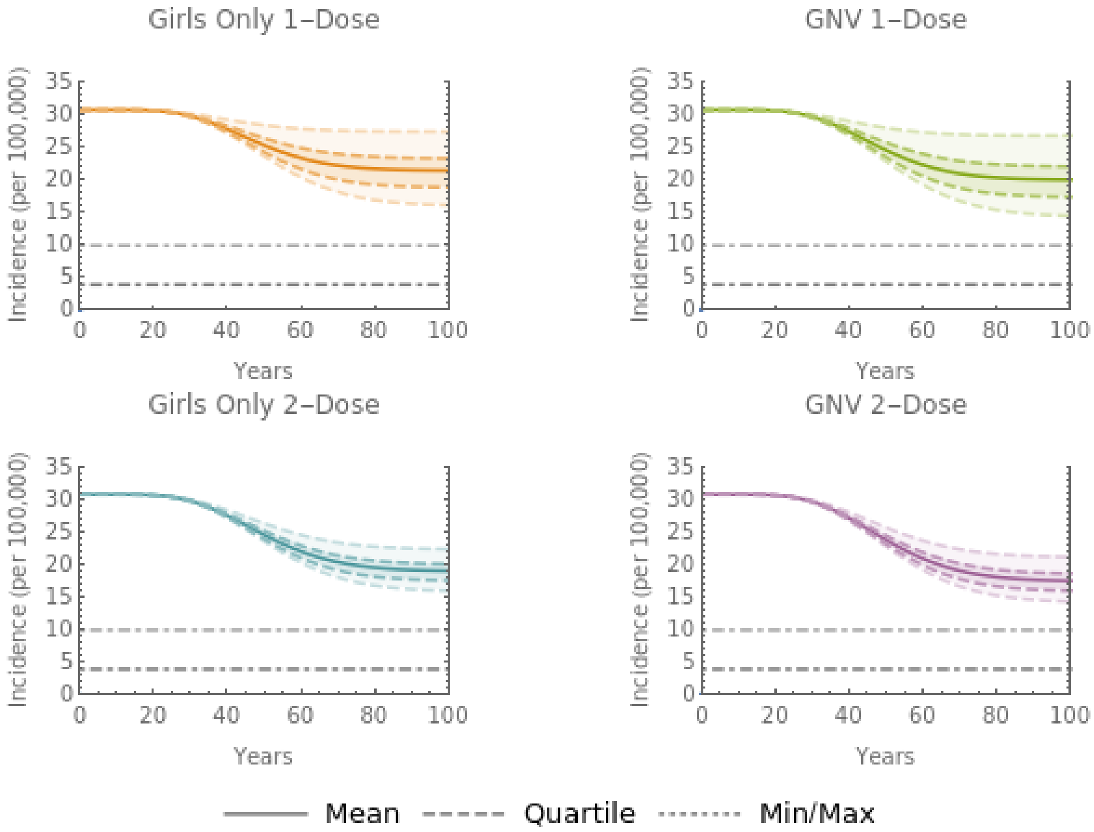


*Figure 3: Median cervical cancer incidence (all HPV types) and range of variation over time for the low coverage scenario, alternative distribution. The darker shaded areas are the middle quartiles, and the lighter shaded areas extend to the min and max. Dash Dotted lines are 4 and 10 cases per 100,000 elimination thresholds.*

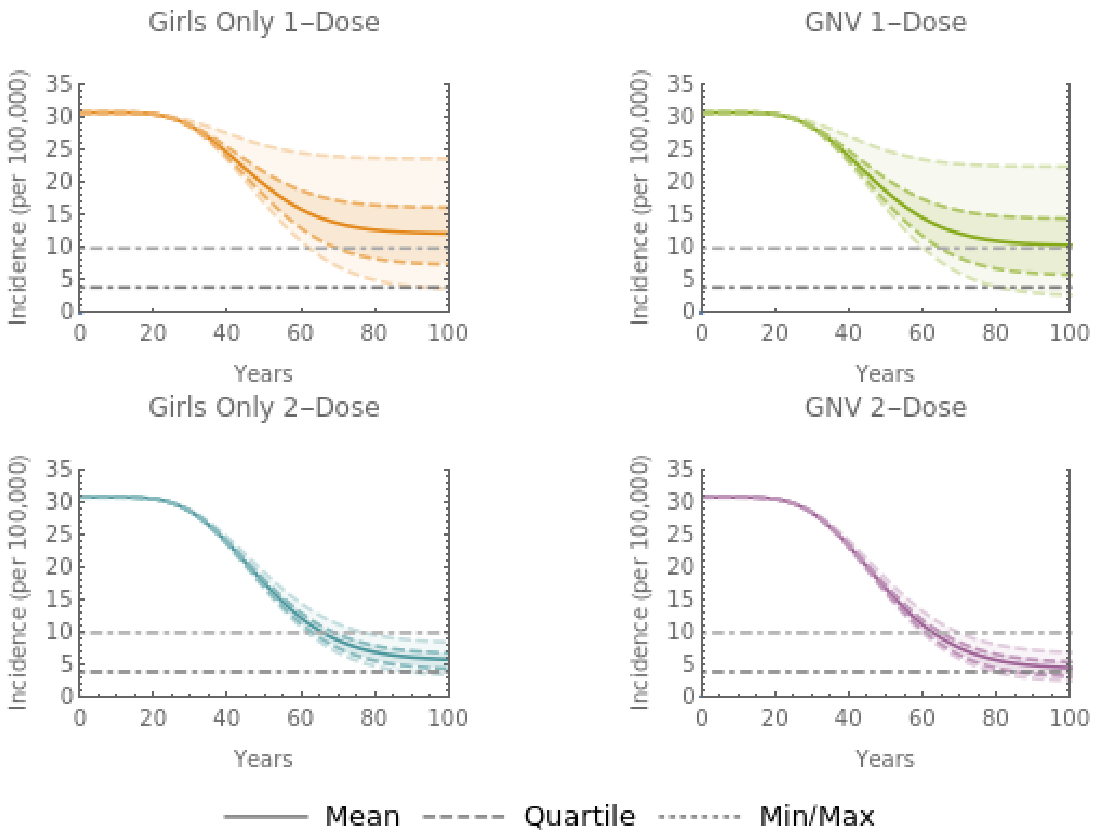


*Figure 74: Median cervical cancer incidence (all HPV types) and range of variation over time for the medium coverage scenario, alternative distribution. The darker shaded areas are the middle quartiles, and the lighter shaded areas extend to the min and max . Dash Dotted lines are 4 and 10 cases per 100,000 elimination thresholds.*

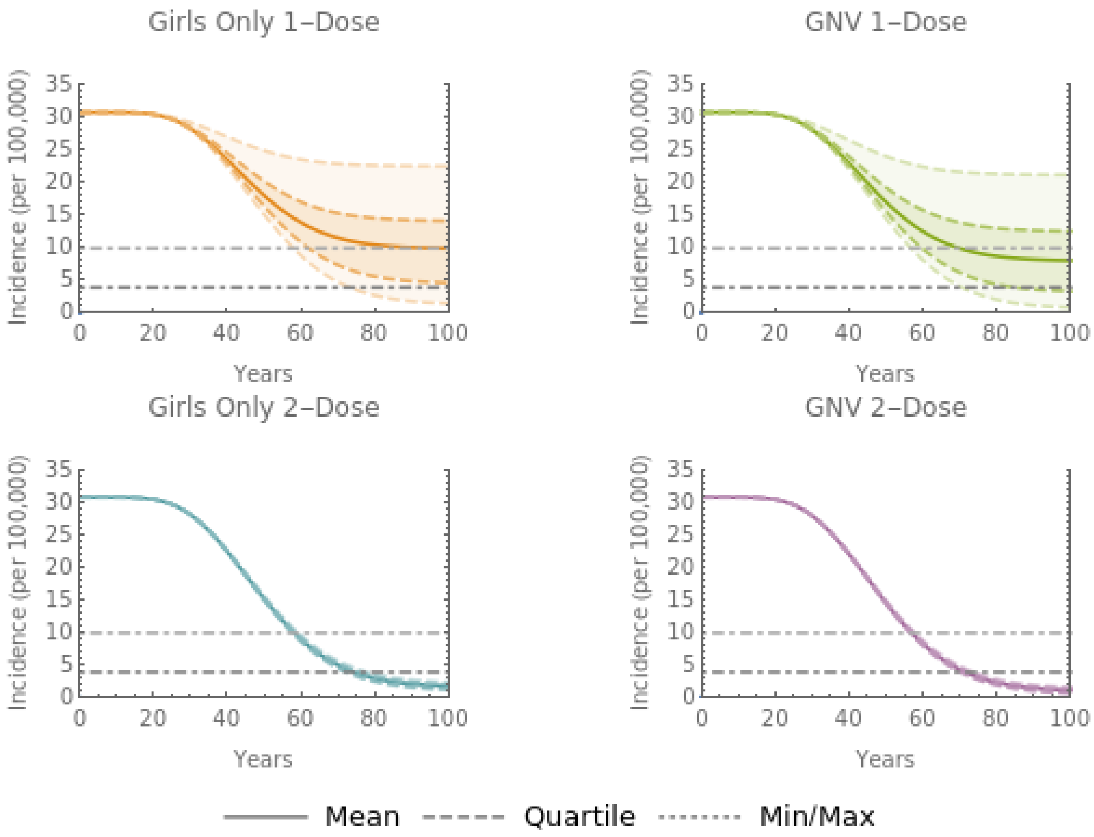


*Figure 75: Median cervical cancer incidence (all HPV types) and range of variation over time for the high coverage scenario, alternative distribution. The darker shaded areas are the middle quartiles, and the lighter shaded areas extend to the min and max. Dash Dotted lines are 4 and 10 cases per 100,000 elimination thresholds.*

# References


[1] Human papillomavirus vaccines. WHO position paper. Wkly Epidemiol Rec. 2022 Dec 16;section;97(50):pp.645-672

[2] Burger EA, Campos NG, Sy S, Regan C, Kim JJ. Health and economic benefits of single-dose HPV vaccination in a Gavi-eligible country. Vaccine. 2018;36(32 Pt A):4823-9

[3] Burger EA, Laprise JF, Sy S, Regan MC, Prem K, Jit M, Brisson M, Kim JJ. Now or later: Health impacts of delaying single-dose HPV vaccine implementation in a high-burden setting. Int J Cancer. 2022 May 5. doi: 10.1002/ijc.34054. Epub ahead of print. PMID: 35512109

[4] Drolet M, Laprise JF, Martin D, Jit M, Benard E, Gingras G, et al. Optimal human papillomavirus vaccination strategies to prevent cervical cancer in low-income and middle-income countries in the context of limited resources: a mathematical modelling analysis. Lancet Infect Dis. 2021

[5] Man I, Georges D, de Carvalho TM, Ray Saraswati L, Bhandari P, Kataria I, Siddiqui M, Muwonge R, Lucas E, Berkhof J, Sankaranarayanan R, Bogaards JA, Basu P, Baussano I. Evidence-based impact projections of single-dose human papillomavirus vaccination in India: a modelling study. Lancet Oncol. 2022 Nov;23(11):1419-1429. doi: 10.1016/S1470-2045(22)00543-5. Epub 2022 Sep 26. PMID: 36174583; PMCID: PMC9622421.

[6] Prem K, Choi YH, Benard E, et al. Global impact and cost-effectiveness of one-dose versus two-dose human papillomavirus vaccination schedules: a comparative modelling analysis. BMC Med. Aug 28 2023;21(1):313. doi:10.1186/s12916-023-02988-3

[7] Barnabas, R.V., et al., Efficacy of Single-Dose Human Papillomavirus Vaccination among Young African Women. NEJM Evidence, 2022. 1(5): p. EVIDoa2100056.

[8] Basu, P., et al., Vaccine efficacy against persistent human papillomavirus (HPV) 16/18 infection at 10 years after one, two, and three doses of quadrivalent HPV vaccine in girls in India: a multicentre, prospective, cohort study. Lancet Oncol, 2021. 22(11): p. 1518-1529.

[9] Rock, K.S., et al., Quantitative evaluation of the strategy to eliminate human African trypanosomiasis in the Democratic Republic of Congo. Parasit Vectors, 2015. 8: p. 532.

[10] Gamerman, D. and H.F. Lopes, Markov chain Monte Carlo: stochastic simulation for Bayesian inference. 2006: CRC press.

[11] Wolfram Research, I., Mathematica, Version 13.1. 2022: Champaign, IL.

[12] Myers, E.R., et al., Mathematical model for the natural history of human papillomavirus infection and cervical carcinogenesis. Am.J.Epidemiol., 2000. 151(12): p. 1158-1171.

[13] Sanders, G.D. and A.V. Taira, Cost-effectiveness of a potential vaccine for human papillomavirus. Emerg.Infect.Dis., 2003. 9(1): p. 37-48.

[14] Elbasha, E.H. and E.J. Dasbach, Impact of vaccinating boys and men against HPV in the United States. Vaccine, 2010. 28(42): p. 6858-67.

[15] De Aloysio, D., et al., Intramuscular interferon-beta treatment of cervical intraepithelial neoplasia II associated with human papillomavirus infection. Acta Obstet Gynecol Scand, 1994. 73(5): p. 420-4.

[16] Insinga, R.P., et al., Progression and regression of incident cervical HPV 6, 11, 16 and 18 infections in young women. Infect Agent Cancer, 2007. 2: p. 15.

[17] Daniels, V., et al., Public health impact and cost-effectiveness of catch-up 9-valent HPV vaccination of individuals through age 45 years in the United States. Hum Vaccin Immunother, 2021: p. 1-9.

[18] Flannelly, G., et al., A study of treatment failures following large loop excision of the transformation zone for the treatment of cervical intraepithelial neoplasia. Br J Obstet Gynaecol, 1997. 104(6): p. 718-22.

[19] Ries, L., et al., SEER Cancer Statistics Review, 1975-2002,. 2005, National Cancer Institute: Bethesda, MD.

[20] Alemany, L., et al., Large contribution of human papillomavirus in vaginal neoplastic lesions: a worldwide study in 597 samples. Eur J Cancer, 2014. 50(16): p. 2846-54.

[21] Serrano, B., et al., Human papillomavirus genotype attribution for HPVs 6, 11, 16, 18, 31, 33, 45, 52 and 58 in female anogenital lesions. Eur J Cancer, 2015. 51(13): p. 1732-41.

[22] Joura EA, Giuliano AR, Iversen OE, et al. A 9-valent HPV vaccine against infection and intraepithelial neoplasia in women. The New England journal of medicine. Feb 19 2015;372(8):711-23. doi:10.1056/NEJMoa1405044

[23] Palefsky JM, Giuliano AR, Goldstone S, et al. HPV vaccine against anal HPV infection and anal intraepithelial neoplasia. The New England journal of medicine. Oct 27 2011;365(17):1576-85. doi:10.1056/NEJMoa1010971

[24] Giuliano AR, Palefsky JM, Goldstone S, et al. Efficacy of quadrivalent HPV vaccine against HPV Infection and disease in males. N Engl J Med. Feb 2011;364(5):401-11. Doi: 10.1056/NEJMoa0909537.

[25] World Population Prospects 2020. 2020 [cited 2020; Available from: https://population.un.org/wpp/Download/Standard/Population/.

[26] Encuesta de salud y hábitos sexuales 2003. 2004: Spain.

[27] Techasrivichien, T., et al., Changes in Sexual Behavior and Attitudes Across Generations and Gender Among a Population-Based Probability Sample From an Urbanizing Province in Thailand. Arch Sex Behav, 2016. 45(2): p. 367-82.

[28] de Sanjose, S., et al., Human papillomavirus genotype attribution in invasive cervical cancer: a retrospective cross-sectional worldwide study. Lancet Oncology, 2010. 11(11): p. 1048-1056.

[29] Zhang, L., et al., Human papillomavirus infections among women with cervical lesions and cervical cancer in Eastern China: genotype-specific prevalence and attribution. BMC Infect Dis, 2017. 17(1): p. 107.

[30] Alemany, L., et al., Human papillomavirus DNA prevalence and type distribution in anal carcinomas worldwide. Int J Cancer, 2015. 136(1): p. 98-107.

[31] Alemany, L., et al., Role of Human Papillomavirus in Penile Carcinomas Worldwide. Eur Urol, 2016. 69(5): p. 953-61.

[32] Castellsague, X., et al., HPV Involvement in Head and Neck Cancers: Comprehensive Assessment of Biomarkers in 3680 Patients. J Natl Cancer Inst, 2016. 108(6): p. djv403.

[33] Vet, J.N., et al., Single-visit approach of cervical cancer screening: see and treat in Indonesia. Br J Cancer, 2012. 107(5): p. 772-7.

[34] Wei, F., et al., Incidence of anogenital warts in Liuzhou, south China: a comparison of data from a prospective study and from the national surveillance system. Emerg Microbes Infect, 2017. 6(12): p. e113.

[35] Kosen, S., et al., The Cost-Effectiveness of Quadrivalent Human Papillomavirus Vaccination in Indonesia. Asian Pac J Cancer Prev, 2017. 18(7): p. 2011-2017.

[36] Choi I, Lee D, Son KB, Bae S. Incidence, cost and gender differences of oropharyngeal and noncervical anogenital cancers in South Korea. BMC Public Health. 2020 Jun 29;20(1):1035. doi: 10.1186/s12889-020-09161-y

[37] Setiawan, D., et al., Cervical cancer prevention in Indonesia: An updated clinical impact, cost-effectiveness and budget impact analysis. PLoS One, 2020. 15(3): p. e0230359.

[38] Self-Reported Population Health: An International Perspective based on EQ-5D, ed. A. Szende, B. Janssen, and J. Cabases. 2014, Dordrecht (NL): Springer.

[39] Termrungruanglert, W., et al., The epidemiologic and economic impact of a quadrivalent human papillomavirus vaccine in Thailand. PLoS One, 2021. 16(2): p. e0245894.

[40] Endarti, D., et al., Evaluation of Health-Related Quality of Life among Patients with Cervical Cancer in Indonesia. Asian Pac J Cancer Prev, 2015. 16(8): p. 3345-50.

[41] Conway, E.L., et al., Quality of life valuations of HPV-associated cancer health states by the general population. Sex Transm Infect, 2012. 88(7): p. 517-21.

[42] Myers, E.R., S. Green, and I. Lipkus. Patient preferences for health states related to HPV infection: visual analog scales vs time trade-off elicitation. in Proceedings of the 21st international papillomavirus conference. 2004. Mexico.
