# Supplementary material for: Modeling the health and economic implications of adopting a 1-dose 9-valent human papillomavirus vaccination program in adolescents in low/middle-income countries: An analysis of Indonesia
Source: PLoS One. 2024 Nov 21;19(11):e0310591. doi: 10.1371/journal.pone.0310591 (PMC11581242; doi:10.1371/journal.pone.0310591)
Supplement: Attachment — Submitted filename: Response to Reviewers.docx [file pone.0310591.s002.docx]

**Response to reviewers**

**Title: “Modeling the health and economic implications of adopting a 1-dose 9-valent human papillomavirus vaccination program in adolescents in low/middle-income countries: an analysis of Indonesia”**

*Reviewer comments are in black, and responses are in blue.*

***--- EDITOR ---***

1. Please ensure that your manuscript meets PLOS ONE's style requirements, including those for file naming. The PLOS ONE style templates can be found at
   [https://journals.plos.org/plosone/s/file?id=wjVg/PLOSOne_formatting_sample_main_body.pdf](https://nam10.safelinks.protection.outlook.com/?url=https%3A%2F%2Fjournals.plos.org%2Fplosone%2Fs%2Ffile%3Fid%3DwjVg%2FPLOSOne_formatting_sample_main_body.pdf&data=05%7C02%7CMolly.Gingrich%40analysisgroup.com%7C45d4dd0fdc6a4ab24ed208dca737a63f%7C2be644011c664caea08292f4c2fca067%7C1%7C0%7C638569104717494780%7CUnknown%7CTWFpbGZsb3d8eyJWIjoiMC4wLjAwMDAiLCJQIjoiV2luMzIiLCJBTiI6Ik1haWwiLCJXVCI6Mn0%3D%7C0%7C%7C%7C&sdata=lojx6ngLJbpSCgWmeM17rL3%2B8jGWxLEavPR4BLZtdzY%3D&reserved=0) and 
   [https://journals.plos.org/plosone/s/file?id=ba62/PLOSOne_formatting_sample_title_authors_affiliations.pdf](https://nam10.safelinks.protection.outlook.com/?url=https%3A%2F%2Fjournals.plos.org%2Fplosone%2Fs%2Ffile%3Fid%3Dba62%2FPLOSOne_formatting_sample_title_authors_affiliations.pdf&data=05%7C02%7CMolly.Gingrich%40analysisgroup.com%7C45d4dd0fdc6a4ab24ed208dca737a63f%7C2be644011c664caea08292f4c2fca067%7C1%7C0%7C638569104717501171%7CUnknown%7CTWFpbGZsb3d8eyJWIjoiMC4wLjAwMDAiLCJQIjoiV2luMzIiLCJBTiI6Ik1haWwiLCJXVCI6Mn0%3D%7C0%7C%7C%7C&sdata=0L%2BtMLZVfmDszFsw%2BnOjopDe2JflVF6eKt%2BIXejSC2c%3D&reserved=0)

Thank you for noting and for providing the style templates. We have updated the manuscript accordingly to meet the PLOS ONE style requirements.

2. Thank you for stating the following in the Competing Interests section:
   "I have read the journal's policy and the authors of this manuscript have the following competing interests:
   Vince Daniels and Kunal Saxena are employees of Merck Sharp & Dohme LLC, a subsidiary of Merck & Co., Inc., Rahway, NJ, USA and shareholders in Merck & Co., Inc., Rahway, NJ, USA.
   Oscar Patterson-Lomba and Andres Gomez-Lievano are employees of Analysis Group, Inc., a consulting company that has provided paid consulting services to Merck & Co., Inc, which funded the development and conduct of this study and manuscript.
   Jarir At Thobari, Nancy Durand, and Evan Myers have received consultancy fees from Merck & Co., Inc."

Please confirm that this does not alter your adherence to all PLOS ONE policies on sharing data and materials, by including the following statement: ""This does not alter our adherence to  PLOS ONE policies on sharing data and materials.” (as detailed online in our guide for authors [http://journals.plos.org/plosone/s/competing-interests](https://nam10.safelinks.protection.outlook.com/?url=http%3A%2F%2Fjournals.plos.org%2Fplosone%2Fs%2Fcompeting-interests&data=05%7C02%7CMolly.Gingrich%40analysisgroup.com%7C45d4dd0fdc6a4ab24ed208dca737a63f%7C2be644011c664caea08292f4c2fca067%7C1%7C0%7C638569104717507622%7CUnknown%7CTWFpbGZsb3d8eyJWIjoiMC4wLjAwMDAiLCJQIjoiV2luMzIiLCJBTiI6Ik1haWwiLCJXVCI6Mn0%3D%7C0%7C%7C%7C&sdata=0aWty1QkjQq2URhWOw5%2BOWX8UcJpDB09Tpm1rIHzs4o%3D&reserved=0)).  If there are restrictions on sharing of data and/or materials, please state these. Please note that we cannot proceed with consideration of your article until this information has been declared.

Please include your updated Competing Interests statement in your cover letter; we will change the online submission form on your behalf.

Thank you for noting this required. We have added the statement, “*This does not alter our adherence to PLOS ONE policies on sharing data and materials*” to the Competing Interests section of our cover letter and included the revised version with our manuscript resubmission.

3. Please include captions for your Supporting Information files at the end of your manuscript, and update any in-text citations to match accordingly. Please see our Supporting Information guidelines for more information: [http://journals.plos.org/plosone/s/supporting-information](https://nam10.safelinks.protection.outlook.com/?url=http%3A%2F%2Fjournals.plos.org%2Fplosone%2Fs%2Fsupporting-information&data=05%7C02%7CMolly.Gingrich%40analysisgroup.com%7C45d4dd0fdc6a4ab24ed208dca737a63f%7C2be644011c664caea08292f4c2fca067%7C1%7C0%7C638569104717514016%7CUnknown%7CTWFpbGZsb3d8eyJWIjoiMC4wLjAwMDAiLCJQIjoiV2luMzIiLCJBTiI6Ik1haWwiLCJXVCI6Mn0%3D%7C0%7C%7C%7C&sdata=8DQakKpdD%2BVFoKdl1DPqe8bsC1ePP5Y%2B7WWPlSvXB4I%3D&reserved=0).

Thank you for informing us of this requirement. We have included the caption “S1 Appendix” as a caption for our Supporting Information file at the end of the manuscript.

***--- REVIEWER 1 ---***

Thank you for the opportunity to review this manuscript. The approach and overall quality of the manuscript is good. However, I have the following comments on the structure, findings and conclusions of this work:

1. First, from the outset, I would like to make a general statement. The whole discussion around one-dose HPV vaccination strategies is based on logistical and sustainability considerations, especially with experience from low and middle-income countries that have recently introduced the vaccine. Without adequate coverage, even a three-dose vaccination dose program would not achieve the desired outcomes. In the 90:70:90 framework, while what "fully-immunized" may change as more evidence becomes available, the 90% coverage threshold may not.

Thank you for your comment. We agree that the desired coverage outcomes are not likely to change regardless of the dosing regimen. It is also true that the dosing regimen that constitutes “fully-immunized” may also change. Our analysis considers both of these situations. We have included a high coverage, 89% initiation rate, for both the single-dose and multi-dose programs. In addition, our distribution of single-dose effectiveness parameters, includes, albeit at low probability, 1 dose having equivalent effectiveness to 2 doses. In these extreme cases, as we have shown (see extreme values in cases avoided), a single-dose program may be either economically more effective or have the same health impact as a 2-dose program. Our primary focus is on the uncertainty of those outcomes based on the uncertainty of the single-dose effectiveness, given the KEN SHE and the India IARC data.

2. Cost of vaccines: the authors state that their vaccine costs inputs were ranging from USD 10-60, yet the current estimates of the gardasil-9 vaccine for instance is around USD 287. Why not use the most realistic vaccine prices, for a country that would be seeking guidance from a paper like this on what policy to adopt? Unless I have missed it, I haven't seen any other different vaccine cost in the model inputs section of the methods. What was the vaccine cost assumption based on?

Thank you for raising this point. Indeed, the vaccine cost of gardasil-9 is USD 287, but that is the US list price. However, as far as we know, there are very few National Immunization Programs (NIPs) in the world that pay the list price, especially LMICs (vaccine dose pricing is typically heavily negotiated as part of NIPs or through procurement channels like PAHO or GAVI, and is based, ultimately, on volume discounting, value, affordability, and other considerations, depending on the context).

We assumed a vaccine base price of $20 USD per dose, including delivery and administration costs, that is intended to represent a potential GAVI-supported price. We explored low ($10) and high ($60) per-dose costs in sensitivity analyses to reflect the current uncertainty in price. Based on the WHO’s Interim 2024 MI4A Vaccine Purchase Database^[1]^, the per-dose price for the 4-valent vaccine in the LMIC setting ranges from $0.81 (GAVI-supported) to $14.40 (non-GAVI-supported), with an average of $5.68 per dose. Since there are no 9-valent vaccine price data for LMICs in this database, vaccine prices were derived from the Setiawan et. al. CE analysis for Indonesia^[2]^, the most robust data available at the time of analysis. We assumed a price for the 9-valent vaccine (including administration costs) of approximately $20 for the base case, with a savings of $10 for the low range (i.e., $10), and a large premium of $40 for high range (i.e., $60). Therefore, the upper range of vaccine price we considered is higher than the price LMICs are expected or observed to pay. We have added the following additional language in the Methods on lines 177 to 179 (underline indicates additions):

“Cost and other economic inputs are detailed in Appendix F. Briefly, vaccine prices were derived from the Setiawan et. al. CE analysis for Indonesia [60] and were in line with WHO-provided vaccine purchase data for GAVI-supported countries [61]. We assumed a vaccine price of $20 United States dollars (USD) per dose including delivery and administration costs that is intended to represent a potential GAVI price.”

3. I think the authors need to have considered additional scenarios for the comparative analyses. For instance, comparing low coverage with a two-dose strategy vs high coverage with a one-dose strategy. A cursory look at figure 1 actually shows that a 1-dose strategy with high coverage actually outperforms a two-dose strategy with low coverage in cervical cancer cases verted. This is the true dilemma many LMICs face. If this work is to be seen as balanced and impartial, the authors should evaluate and discuss this in detail.

Thank you for your comment. It is true that *if* high coverage (i.e., a high initiation rate) is achieved for a 1-dose program, it can avoid more cases when compared to a 2-dose program where only a low initiation rate is achieved. However, there is little evidence that by virtue of choosing a 1-dose program over a 2-dose program, higher initiation rates will be achieved. Therefore, we do not believe it is reasonable, and, in fact, may be misleading, to directly compare these scenarios to each other, which would be implying that initiation rates would increase from 35% to 89% if the country merely opted for a single-dose program instead of a 2-dose program. Variation of this magnitude would require substantially different implementation strategies, regardless of whether it was a single-dose or 2-dose program. For example, having a school-based program is known to lead to potentially high initiation rates compared with non-school-based programs^[3]^. However, to our knowledge, there is no evidence that would imply that the initiation rate for a 2-dose school-based program would be different from that of a single-dose school-based program, nor that a school-based 1-dose program would become non-school-based if a 2-dose program were to be implemented. Identifying, specifying, and costing realistic scenarios that would generate such extreme differences in coverage is a substantial research project that we believe is out of scope for the current analysis.

Still, we had already noted the following in the Discussion on lines 529 to 535: “*all else equal, the 1st dose compliance rates may be marginally larger in a single-dose program as compared to a 2-dose program, although there is no clear empirical evidence suggesting that initiation rates will be larger in settings with a vaccination program with fewer recommended vaccine doses (for example, the change in recommendations from 3 to 2 doses of HPV vaccine in 2016 in the USA did not seem to have a significant effect on the national trends of initiation rates in the subsequent years)*”. There seems to be an expectation that a 1-dose program will help to boost coverage; however, the evidence available so far does not indicate that the implementation of 1-dose programs or the reduction of doses in multi-dose programs leads to a significant increase in coverage rates. For example, in August 2022, the Joint Committee on Vaccination and Immunisation (JCVI) advised^[4]^ a change in the HPV vaccination schedule from 2 doses to 1 dose in the routine adolescent program in the UK. This was followed by a WHO recommendation^[5]^ in December 2022 for a 1- or 2-dose schedule for girls aged 9-14 years. Based on the vaccination coverage data reported by the WHO^[6]^, there has been little to no change in initiation rates in females nor males in the UK since these recommendations were made, with initiation rates (i.e., first dose coverage) remaining at 68% in 2022 and 2023 for females, and increasing slightly from 62% to 64% for males. In addition, Australia moved to a 1-dose program^[7]^ in February of 2023. The WHO coverage data shows that in Australia initiation rates *decreased* in 2023 compared to 2022 (from 77% to 71% in females, and from 72% to 65% in males). A snapshot of these data in the WHO tool is shown below:

We have added the following additional language in the Discussion on lines 554 to 566 to note that we think this comparison is not realistic at this point and thus not informative (underline indicates additions; ~~strikethrough~~ indicates deletions):

“all else equal, the 1st dose compliance rates may be marginally larger in a single-dose program as compared to a 2-dose program, although there is limited ~~no clear~~ empirical evidence suggesting that initiation rates will be larger in settings with a vaccination program with fewer recommended vaccine doses. ~~(f~~ For example, the change in HPV vaccine recommendation~~s~~ from 3 doses to 2 doses ~~of HPV vaccine~~ in 2016 in the USA, and 2 doses to 1 dose in 2022 in the United Kingdom (UK), did not seem to have a significant effect on the national trends of initiation rates in the subsequent years [70],[71],[72], [73]. Moreover, the introduction of a 1-dose HPV program in 2023 in Australia was associated with a decrease in vaccination initiation that year [74],[75]. Given that the difference in initiation rates between a 1- and 2-dose program depends on multiple factors and may be highly variable across countries and settings, until further evidence is available, any direct comparison between “1-dose with high coverage” and “2-dose with low coverage” scenarios would be based on unrealistic expectations and could result in misleading conclusions.

4. In lines 402-412, the authors clearly state that while the two-dose strategy is cost-saving in all coverage scenarios, in high dose scenario, the 1-dose strategy becomes cost-saving but the 2-dose one has very low ICER. Since this represents the nearest picture to the true situation in low and middle-income countries of different contexts, this should be discussed and made part of the conclusion.

Thanks for your comments. However, we respectfully disagree with the Reviewer that high coverage is the likely situation in LMIC settings. For coverage assumptions, we relied on the recent analysis of WHO and UNICEF vaccination coverage data in Bruni et al. 2021^[8]^. They observed that among LMIC countries with vaccination programs, the median and mean initiation rates (first dose coverage) are about 80% and 68%, respectively. This corresponds most closely to our “medium coverage” scenario. Because a wide range of initiation rates are observed across LMICs, we considered it important to explore the range of observed coverage scenarios by grouping them into high, medium, and low coverage/completion scenarios. The approach is described in the Methods section. All three coverage scenarios are included in the Results, and all are discussed with equal weight in the Discussion. Policymakers can decide which scenario best represents their situation.

Regarding the specific point raised by the Reviewer, we did note in the manuscript that under the alternative distribution scenario, for medium and high coverage scenarios, the girls-only 1-dose program is cost-saving, while the girls-only 2-dose program has a very low ICER, with the GNV 2-dose ICERs still being well below the 1xGDP willingness to pay (WTP) threshold. We emphasized this observation at the end of the Results section on lines 443 to 445 when we noted that “*results indicate that, in general, the probability of 1-dose programs being cost-effective is higher in terms of acceptability curves, particularly for low WTP thresholds and for scenarios with high coverage, high dose price, or high discount rate*.” Thus, we agree, and have noted in the results section that a 1-dose girls-only strategy can be cost-saving in the high coverage, alternative distribution scenario. However, it is also true that even in this scenario, the population will achieve more health benefits from 2-dose strategies for relatively little additional cost. In other words, if the WTP threshold is very low (close to 0), then the 1-dose girls-only program is the best strategy since it is cost-saving. However, in most settings, the WTP threshold is not close to 0 and may be close to 1xGDP. In those cases, the best economic choice is the strategy with the highest health benefits that has an ICER below the WTP threshold. Based on our results, the girls-only 2-dose and the GNV 2-dose programs bring more health benefits than the 1-dose strategy, and both have ICERs well below the 1xGDP WTP threshold. We have added the following additional language in the Discussion on lines 498 to 504 highlighting this point (underline indicates additions):

“Under the alternative distribution scenario, for medium and high coverage, the 1-dose girls-only strategy can be cost-saving, and thus is the economically preferred strategy when the WTP threshold is close to 0 (less than about $70/QALY). However, in most settings, the WTP threshold is often higher and as high as 1xGDP, in which case the economically optimal strategy is the one with an ICER below the chosen WTP threshold. Our results indicate that both the girls-only 2-dose and GNV 2-dose strategies have ICERs well below the 1xGDP WTP threshold and thus provide more population health benefits for relatively little additional cost.”

5. One argument for adoption of 1-dose strategies is that financial resources saved can be invested in other public health programs like screening and treatment, which would have impact on incidence and mortality of cervical cancer earlier than vaccination. What would be the budget impact of adopting a one-dose strategy as opposed to a two-dose strategy, especially for no-Gavi supported countries or those transitioning out of support? Even though the model adapted in this study was not able to do this additional scenarios, it would add value to cover this in the discussion section, since that's what decision-makers really need to know.

Thank you for bringing up this important point. How much would be saved in a real-world single-dose program is difficult to estimate as it depends on many factors that we have not or cannot examine in our model. For example, we make the assumption that the dose price in a single-dose program would be the same as the dose price in a multi-dose program. This would not likely be the case as NIP dose prices are negotiated (certainly in non-Gavi/PAHO settings) and are dependent on many factors, including value-based and/or volume-based discounts, among other possible considerations, in negotiations. In addition, there is a wide range of possible uses that a particular country may put this money towards, many of which may not be directly related to HPV. However, as you have noted, screening and treatment programs are a possible use for these funds. Even in this specific use case, estimating the costs and impact (on HPV and related diseases) is very dependent on a detailed understanding of the barriers to implementing a screening and treatment program in the particular LMIC country setting. In their recent systematic review examining barriers to cervical cancer screening in Africa, Mantula, et al. conclude that “barriers to cervical cancer screening. . . are multifaceted . . .” including socio-cultural, awareness, accessibility, lack of training, lack of equipment and facilities for testing, analysis, and treatment, staff, and health practitioner shortages^[9]^. Most of these issues will require substantial investments that would not likely be compensated by the annual cost reductions resulting from administering 1 dose instead of 2 doses of the HPV vaccine, and may take many decades to successfully implement. Studying the economic and health impact of such aspirational programs and what could be done to enable successful implementation is an important area of research that is out of the scope for this analysis.

We have made the following additions related to this comment to the study limitations paragraphs in the Discussion section (underline indicates additions; ~~strikethrough~~ indicates deletions):

Lines 546 to 550:“Third, we assumed that the per-dose price for a single-dose program would be the same as a multi-dose program. This is unlikely to be true outside of the GAVI context as actual NIP dose pricing is the result of negotiation that may result in lower volume discounting for few doses as well as other factors that affect dose pricing negotiations.”

Lines 552 to 553:“However, there are substantial barriers to implementing these programs in LMICs [66],[67],[68].”

Lines 566 to 571: “We also did not model the idea of replacing the 2-dose girls-only program with a 1-dose program plus cervical screening and treatment because, as noted above, the introduction of a new screening and treatment program ~~is unlikely in LMIC setting~~s has substantial barriers that may take several years to implement, and will likely require substantially more funding to implement and maintain than the funds that may be saved in vaccine costs in a 1-dose instead of 2-dose HPV vaccine program.”

Screening

6. The decision to adopt the KENSHE results into the model is a good one. Are the authors willing to update their model as more data from the trail (follow-up is continuing) become available?

Thank you for your comment. Yes, future studies will include updating our model inputs once more longer-term data from KEN SHE and other trials (e.g., ESCUDDO and the planned Merck trial^[10]^) become available. We have added a note explaining this in the Conclusions on lines 585 to 587 (underline indicates additions):

“Due to the rapidly evolving evidence on this topic, health economic models need to be updated as additional trial results become available.”

We have also added a note regarding ongoing studies for 1-dose regimens to the Introduction on lines 86 to 92 (underline indicates additions):

“More evidence on 1-dose HPV vaccination will become available in the coming years from ongoing clinical trials that were designed to study the efficacy of the single-dose schedule, including long-term results from the KEN SHE trial, two ongoing randomized, controlled trials, ESCUDDO (NCT03675256) and PRISMA (NCT05237947), and two recently announced randomized, controlled trials (one in females ages 16-26 years old, and another in males ages 16-26 years old) [38].”

7. Because of number 4 above, I find the conclusion rather shallow or even misleading.

Thank you for this critique. To reflect point 4 above, we have noted in the conclusions that under high coverage, 1-dose strategies may be cost-saving options, but 2-dose strategies will still be a preferred strategy due to the prevention of more cancer cases and the ICER being under the applicable WTP threshold. We have also highlighted that rapidly evolving evidence on this topic will require updates to cost-effectiveness model estimates. The following text has been added to the Conclusions on lines 583 to 587 (underline indicates additions):

“While 1-dose strategies can be cost-saving in high coverage scenarios, 2-dose strategies may still be preferred due to the ability to provide greater health benefits while maintaining cost-effectiveness. Due to the rapidly evolving evidence on this topic, cost-effectiveness models should be updated as additional trial results become available.”

***--- REVIEWER 2 ---***

Thank you for the chance to review this interesting paper. I believe these results will be beneficial to the field moving forward. I have a few comments that I believe will make the paper stronger.

1. Clearer description of why the KEN-SHE data were used in conjunction with data from IARC India earlier in the methods section. This is mentioned, but could be clearer.

Thank you for your suggestion. While we could have used IARC data only, this would have ignored the more robust data from the KEN SHE RCT. Moreover, using KEN SHE data, in combination with the IARC data, resulted in higher estimates for 1-dose durability of protection in the short-term (during the first 10 years) compared to an estimation approach using only the IARC data. We have clarified this in the Methods by adding the following text on lines 216 to 223 (underline indicates additions; ~~strikethrough~~ indicates deletions):

“This was considered the alternative distribution because the vaccine effectiveness evidence from India IARC is arguably less robust than the efficacy data from KEN SHE due to limitations of the ~~India~~ IARC study (e.g., non-randomized design which can lead to confounding and bias [62]). Moreover, using the KEN SHE data, in combination with the IARC data, resulted in higher estimates for 1-dose durability of protection in the short-term (during the first 10 years) compared to an estimation approach using only the IARC data. A detailed description of these trial data and the methods used to generate distributions can be found in Appendix B.”

2. While results of the study indicate cost-effectiveness for a 2-dose vaccination program, the discussion would benefit from some comments about how realistic this would be for a single-payer system, and barriers that may impact real-world cost-effectiveness of such a vaccination program.

We acknowledge that in LMICs, having to administer the 2^nd^ dose (especially in high coverage scenarios) could make the 2-dose program more expensive for the government’s budget, particularly in the short-term (e.g., in the first 5-10 years), despite being cost-effective in the long-term (e.g., over a 100-year period). In addition to the simple budgetary cost of the second dose, there are potential logistical and financial barriers to getting the second dose administered. These are some of the considerations that impact the true cost of the 2-dose implementation that policymakers need to consider. However, there are also factors, particularly assumptions around 1-dose effectiveness that introduce a potentially substantial risk when introducing a 1-dose program compared to a 2-dose program. Our analyses do inform the cost-effectiveness aspect of this decision, but not the affordability aspect. Further research is needed to shine light on the affordability aspects of the different dosing schedules of HPV vaccination on a country-by-country basis. We have added the following additional language in the Discussion highlighting this important point on lines 505 to 510 (underline indicates additions):

“While this work is focused on the potential health and long-term economic risk of a single-dose program given the uncertainty in 1-dose effectiveness, in the real world, policy makers often consider many other factors that could influence this decision, which are not included in this analysis, such as budgetary constraints, logistical barriers, equity considerations, negotiated per-dose price (which may be higher for 1-dose) among other considerations, that will influence the ultimate choice of program and its health and economic success.”

3. The primary limitation of the model is that it assumes that the degree of protection is equal for all disease areas, both sexes, HPV types, and endpoints. This is a major limitation that requires some additional comments. Is there literature that provides context about how 1-dose vs. 2-dose regimens might have varying impact on these characteristics? What guardrails would be valuable for implementing this type of program in similar LMICs given this limitation? How can this limitation be better addressed in future research?

Indeed, this is an important limitation that we proactively acknowledged. However, it is a limitation of the data, not the model itself. To address this limitation, further clinical trials are required, and several, as discussed in the Introduction and Conclusion sections, are currently in progress or starting, including ESCUDO, PRISMA, and a Merck-sponsored single dose trial. However, at this point in time, the best available data on single-dose effectiveness against female persistent infection in the cervix comes from (KEN SHE) for the 9vHPV vaccine or the India IARC observational study for the 2vHPV vaccine. We then make the natural, and usual, assumptions that:

- The durability will be the same for all sites, end-points, and sexes
- Effectiveness of 1-dose is reduced by the same factor for all sites, endpoints, and sexes

When data specific to other sites, end-points, and sexes, become available, we will incorporate that into our future modeling. We have added a note regarding ongoing studies for 1-dose programs in the Introduction on lines 86 to 92 (underline indicates additions):

“More evidence on 1-dose HPV vaccination will become available in the coming years from ongoing clinical trials that were designed to study the efficacy of the single-dose schedule, including long-term results from the KEN SHE trial, two ongoing randomized, controlled trials, ESCUDDO (NCT03675256) and PRISMA (NCT05237947), and two recently announced randomized, controlled trials (one in females ages 16-26 years old, and another in males ages 16-26 years old)[38].”

We have also noted that cost-effectiveness models should be updated as clinical trial results become available in the Conclusion on lines 585 to 587 (underline indicates additions):

“Due to the rapidly evolving evidence on this topic, health economic models need to be updated as additional trial results become available.”

4. The authors did not model the introduction of cervical cancer screening programs, which is a reasonable decision as it is out of scope for the topic being investigated. As such, it is my opinion that the statement about LMICs being “unable to introduce robust and organized screening programs”, should be removed as it minimizes the sociocultural, political, and health system barriers to organized screening in LMICs which also influence vaccination uptake, and discounts the value of the grassroots efforts aimed at increasing awareness about cervical cancer and the importance of screening.

Thank you for the suggestion. We have revised the language in the limitations section of the Discussion on lines 550 to 553 as follows (underline indicates additions; ~~strikethrough~~ indicates deletions):

“We did not consider the introduction of cervical screening and treatment programs, either as a means for monitoring the 1-dose program, or for accelerating the time to elimination. However, ~~which is a reasonable omission given that LMICs currently have been, in general, unable to introduce robust and organized~~ there are substantial barriers to ~~screening~~ implementing these programs in LMICs [66],[67],[68].~~, and which is reflective of the low screening rates in Indonesia [69].~~”

1. <https://www.who.int/teams/immunization-vaccines-and-biologicals/vaccine-access/mi4a/mi4a-vaccine-purchase-data>
2. Setiawan, D., et al., Cervical cancer prevention in Indonesia: An updated clinical impact, cost-effectiveness and budget impact analysis. PLoS One, 2020. 15(3): p. e0230359.
3. Bruni L, Saura-Lázaro A, Montoliu A, Brotons M, Alemany L, Diallo MS, Afsar OZ, LaMontagne DS, Mosina L, Contreras M, Velandia-González M, Pastore R, Gacic-Dobo M, Bloem P. HPV vaccination introduction worldwide and WHO and UNICEF estimates of national HPV immunization coverage 2010-2019. Prev Med. 2021 Mar;144:106399. doi: 10.1016/j.ypmed.2020.106399. Epub 2020 Dec 31. Erratum in: Prev Med. 2022 Feb;155:106925. PMID: 33388322.
4. <https://www.gov.uk/government/publications/single-dose-of-hpv-vaccine-jcvi-concluding-advice/jcvi-statement-on-a-one-dose-schedule-for-the-routine-hpv-immunisation-programme>
5. <https://www.who.int/news/item/20-12-2022-WHO-updates-recommendations-on-HPV-vaccination-schedule>
6. <https://immunizationdata.who.int/global/wiise-detail-page/human-papillomavirus-(hpv)-vaccination-coverage>
7. <https://www.health.gov.au/ministers/the-hon-mark-butler-mp/media/change-to-single-dose-hpv-vaccine>
8. Bruni L, Saura-Lázaro A, Montoliu A, Brotons M, Alemany L, Diallo MS, Afsar OZ, LaMontagne DS, Mosina L, Contreras M, Velandia-González M, Pastore R, Gacic-Dobo M, Bloem P. HPV vaccination introduction worldwide and WHO and UNICEF estimates of national HPV immunization coverage 2010-2019. Prev Med. 2021 Mar;144:106399. doi: 10.1016/j.ypmed.2020.106399. Epub 2020 Dec 31. Erratum in: Prev Med. 2022 Feb;155:106925. PMID: 33388322.
9. Mantula, F., Toefy, Y. & Sewram, V. Barriers to cervical cancer screening in Africa: a systematic review. *BMC Public Health* **24**, 525 (2024). https://doi.org/10.1186/s12889-024-17842-1
10. <https://www.merck.com/news/merck-announces-plans-to-conduct-clinical-trials-of-a-novel-investigational-multi-valent-human-papillomavirus-hpv-vaccine-and-single-dose-regimen-for-gardasil-9/>
